# Description of 47 new species of the New Caledonian endemic caddisfly genus *Agmina* Ward & Schefter (Trichoptera, Ecnomidae)

**DOI:** 10.3897/zookeys.956.51592

**Published:** 2020-08-06

**Authors:** Marianne Espeland, Tin Sjöberg, Kjell Arne Johanson

**Affiliations:** 1 Arthropoda Department, Zoological Research Museum Alexander Koenig, Bonn, Germany Zoological Research Museum Alexander Koenig Bonn Germany; 2 Zoology Department, Swedish Museum of Natural History, Box 50007, 10405 Stockholm, Sweden Swedish Museum of Natural History Stockholm Sweden

**Keywords:** caddisflies, diversity, New Caledonia, new species, rivers, streams

## Abstract

New Caledonia has a rich Trichoptera fauna with over 200 known species, most of them endemic. The total diversity has been estimated as high as 300 to 600 species. The endemic genus *Agmina* Ward & Schefter (Ecnomidae, Trichoptera) includes 28 described species. Based on male genitalia morphology and previously published molecular data another 47 new species in the genus are described, namely *Agmina tuberosa***sp. nov.**, *A. semiovale***sp. nov.**, *A. rocheta***sp. nov.**, *A. tenuisa***sp. nov.**, *A. multidentata***sp. nov.**, *A. cornuta***sp. nov.**, *A. sagittata***sp. nov.**, *A. circulata***sp. nov.**, *A. digitata***sp. nov.**, *A. longispina***sp. nov.**, *A. magnahamata***sp. nov.**, *A. longicordata***sp. nov.**, *A. campanula***sp. nov.**, *A. semicampanula***sp. nov.**, *A. cunicula***sp. nov.**, *A. cerritula***sp. nov.**, *A. monstrosa***sp. nov.**, *A. rectangulata***sp. nov.**, *A. chela***sp. nov.**, *A. piscaria***sp. nov.**, *A. amplexa***sp. nov.**, *A. caraffa*, **sp. nov.**, *A. rostrata***sp. nov.**, *A. dathioensis***sp. nov.**, *A. rougensis***sp. nov.**, *A. viklundi***sp. nov.**, *A. lata***sp. nov.**, *A. falx***sp. nov.**, *A. guttula***sp. nov.**, *A. amieuensis***sp. nov.**, *A. spina***sp. nov.**, *A. complexa***sp. nov.**, *A. dognyensis***sp. nov.**, *A. mana***sp. nov.**, *A. anterohamata***sp. nov.**, *A. curvatacua***sp. nov.**, *A. recurvata***sp. nov.**, *A. taoensis***sp. nov.**, *A. triangulata***sp. nov.**, *A. bleuensis***sp. nov.**, *A. touhoensis***sp. nov.**, *A. wardi***sp. nov.**, *A. parallela***sp. nov.**, *A. christinae***sp. nov.**, *A. brevis***sp. nov.**, *A. ninguana***sp. nov.**, and *A. scopula***sp. nov.** Additionally, new records are provide for the species *A. acula* Ward, 2003, *A. artarima* Ward & Schefter, 2000, *A. berada* Ward & Schefter, 2000, *A. bimaculata* Ward & Schefter, 2000, *A. cheirella* Ward, 2003, *A. comata* Ward, 2003, *A. diriwi* Ward & Schefter, 2000, *A. hamata* Ward & Schefter, 2000, *A. hastata* Ward & Schefter, 2000, *A. hirta* Ward & Schefter, 2000, *A. jepiva* Ward & Schefter, 2000, *A. joycei* Ward & Schefter, 2000, *A. kapiwa* Ward & Schefter, 2000, *A. kara* Ward & Schefter, 2000, *A. mariae* Ward & Schefter, 2000, *A. nodosa* Ward, 2003, *A. panda* Ward & Schefter, 2000, *A. padi* Ward & Schefter, 2000, *A. parie* Ward & Schefter, 2000, *A. rhara* Ward & Schefter, 2000, *A. urugi* Ward & Schefter, 2000, and *A. vuegi* Ward & Schefter, 2000. With a total of 75 described species *Agmina* is one of the largest animal radiations in New Caledonia. Nothing is known about the early stages of any of the species in this genus.

## Introduction

New Caledonia in the southwest Pacific is the smallest of the original biodiversity hotspots (Myers et al. 2000) and has a rich both terrestrial and freshwater insect fauna (e.g., Balke et al. 2007; Espeland and Johanson 2010a, b; Papadopoulou et al. 2013; Gibbs and Lees 2014). The islands have been shown to be especially rich in caddisflies (Trichoptera) with almost 250 described species and an estimated total of 300 to 600 species (Espeland and Johanson 2010a; Johanson and Wells 2019). The caddisfly genus *Agmina* Ward & Schefter, 2000 (Ecnomidae) currently contains 28 species all endemic to New Caledonia. Approximately 40% of these species were described based on single specimens and it has been predicted that the total number of species is probably much higher (Ward and Schefter 2000; Ward 2003; Johanson and Wells 2019; Wells et al. 2019). Support for this comes from a phylogenetic study showing that there are at least 47 undescribed species in the genus, making it one of the largest known animal radiations on New Caledonia (Espeland and Johanson 2010a), now possibly surpassed only by eumolpine leaf beetles (Papadopoulou et al. 2013). The study by Espeland and Johanson (2010a) indicated that *Agmina* split from its closest relatives around 36 mya just after the island reappeared from being submerged for much of the Palaeocene and first half of the Eocene (e.g., Paris 1981; Aichison et al. 1998; Crawford et al. 2003; Pelletier 2007; Cluzel et al. 2012). The earliest radiation was dated to the early Miocene. More than half the species appear to be adapted to the nutrient poor, but toxic, nickel-rich ultramafic substrate currently found on approximately one third of the island (Espeland and Johanson 2010a), which generally has been shown to have a poor aquatic macroinvertebrate diversity compared to other substrates (Mary 2002).

Here we describe 47 new species in the genus *Agmina* increasing the number of known species by 270%, to 75 species. Several of the new species are based on singletons shown to be good species in the molecular phylogeny of Espeland and Johanson (2010a). With this report, 286 species of caddisflies are now known from New Caledonia.

## Materials and methods

The material used in this study was collected in Malaise traps and light traps on the New Caledonian Grande Terre during three expeditions between 2001 and 2006. The Malaise traps were set in place for approx. two weeks at a time, the light traps were operated overnight only. All material was sampled directly into 80% alcohol and transported to the laboratory at the Swedish Museum of Natural History (**NHRS**) for sorting and determination. The determinations were carried out using the information in Ward and Schefter (2000) and Ward (2003). Material of all *Agmina* species in the samples was included in a phylogenetic analysis (Espeland and Johanson 2010a) which resulted in a tree with seven monophyletic clades including 22 previously described species and 47 undescribed species. The seven clades are used below to group the species according to the phylogenetic position rather than morphological similarities, and diagnosis of the species groups are therefore not included. Extraction of DNA from the specimens was done from the individual abdomens, which were macerated during that process. The abdomens were dehydrated in absolute alcohol and temporarily mounted in Euparal on a microscope slide before examination and drawing. All drawings were produced in pencil on plain white A4 paper sheets using a drawing tube mounted on a Leitz Ortholux II. After the drawings were completed the abdomens were returned to the alcohol vial with the rest of the animal. Each pencil illustration was digitised in a scanner at low resolution and thereafter used as a background layer in Adobe Photoshop 8.0. The illustrations were completed after being re-drawn on a new layer using a Wacom drawing pad before the background layer was deleted. The nomenclature applied to the genitalic morphology follows that of Johanson (2017). Specimens in this study are deposited in the following repositories:

**MNHN**Muséum national d’Histoire naturelle, Paris, France;

**NHRS**Swedish Museum of Natural History, Stockholm, Sweden.

## Results

### Descriptions

Phylum: Arthropoda von Siebold, 1848

Class: Insecta Linnaeus, 1758

Order: Trichoptera Kirby, 1813

Superfamily Hydropsychoidea Curtis, 1835

Family: Ecnomidae Ulmer, 1903

Genus: *Agmina* Ward & Schefter, 2000

### Species group 1, *tuberosa* group

Included species in this group are: *Agmina tuberosa* sp. nov., *A. semiovale* sp. nov., *A. rocheta* sp. nov., *A. tenuisa* sp. nov., *A. multidentata* sp. nov., and *A. cornuta* sp. nov.

#### Agmina tuberosa sp. nov.

Taxon classificationAnimaliaTrichopteraEcnomidae

F2CA5C49-6622-5EA2-B8C7-5A1C471F435D

http://zoobank.org/69CE945D-293D-4A0F-9F82-4A13533C2724

Figs 1–5

##### Diagnosis.

*Agmina tuberosa* sp. nov. resembles many other *Agmina* species in having large, oval superior appendage, but is distinguished from other species by the large inferior appendages, each with a long dorsal and short ventral branch in lateral view, and the sternal processes reaching to half length of the inferior appendages. The genitalia are similar to those of *A. semiovale* sp. nov. from which it is distinguished by the presence of a row of teeth-like megasetae on the mesal margin of each paramere in dorsal view.

**Figures 1–5.**
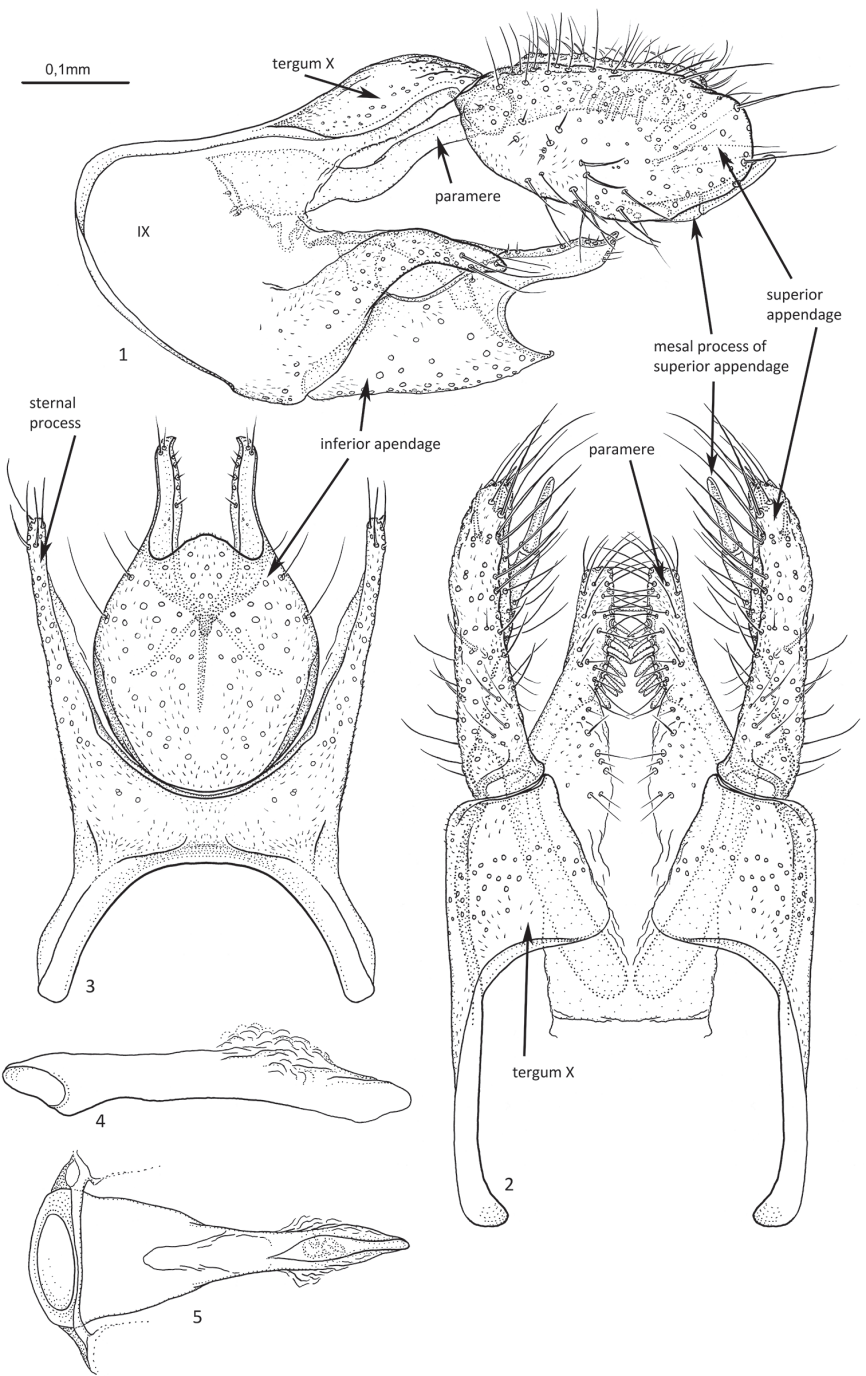
*Agmina tuberosa* sp. nov. male holotype **1** genitalia, left lateral view **2** genitalia, dorsal view **3** genitalia, ventral view **4** phallus, lateral view **5** phallus, ventral view.

##### Etymology.

*Tuberosa*, from Latin, meaning potato, named for the superior appendage being potato-shaped in lateral view.

##### Material examined

**. *Holotype***: New Caledonia – **Province Sud** • ♂; Rivière des Lacs, 1.1 km NW Lac en Huit, 4.9 km NW summit of Pic du Grand Kaori; loc#078; 22°15.195'S, 166°52.178'E; 10.xii.2003; light trap; leg. KA Johanson; MNHN.

***Paratype***: New Caledonia – **Province Sud** • 1 ♂; Rivière des Lacs, above waterfall at Chutes de Madeleine; 22°13.930'S, 166°51.633'E; 243 m; 23.xi.2003; light trap; loc#042; leg. KA Johanson; NHRS.

##### Measurements.

Fore wing length 4.2–4.8 mm (*N* = 2). Total length of genitalia: 0.6 mm.

##### Description.

***Genitalia***: In lateral view, segment IX widely rounded anteriorly, apex located dorsally; in ventral view anteriorly widely U-shaped. Sternal processes, lateral view, with apex not exceeding posterior apex of tergum X, narrowing along their length, curved ventro-posteriorly at mid-length; in ventral view, slender, straight, slightly diverging along their length. Tergum X smoothly convex dorsally, in lateral view longer than high; in dorsal view, mesally separate, axe-shaped with straight inner margins. Parameres robust, starting before tergum X, long, narrow; in lateral view running parallel with dorsal margin of segment IX, X, and superior appendage, ending before apex of superior appendage; in dorsal view, separated and narrowing along their length, each with truncate apex, inner margin with row of megasetae. Superior appendage, in lateral view, large, oval, with posterad spine-like mesal process present on ventromesal margin exceeding the main branch posteriorly; in dorsal view uniformly narrow, running almost parallel, slightly curving inwards towards blunt apex. Mesal processes straight, orientated slightly mesally. Inferior appendage with posterad orientated long dorsal branch slightly dorsally curving at acute apex; dorsal branch widely separated from short, triangular, ventral branch; in ventral view large, wide, oval, with posterad orientated dorsal branches, ventral branch forming central lobe. Phallus, in lateral view as long as segment IX, slender and slightly curving downwards; in ventral view uniformly tapering along its length.

##### Additional information.

This species was referred to as “sp. 26” in Espeland and Johanson (2010a).

#### Agmina semiovale sp. nov.

Taxon classificationAnimaliaTrichopteraEcnomidae

EC9B46C5-54BC-5563-A189-E09F495F62A8

http://zoobank.org/49E2FB6D-0222-4EFB-9DB8-273D468257B5

Figs 6–9

##### Diagnosis.

*Agmina semiovale* sp. nov. resembles many other *Agmina* species in having a large semi-oval shaped superior appendage in lateral view. The species is distinguished from the other species by the presence of very large parameres that partly exceeds the superior appendages dorsally as seen in lateral view, and in dorsal view forming an oval basis and two broad posterad branches. The genitalia are similar to those of *A. tuberosa* sp. nov. from which it is distinguished by the absence of a row of teeth-like megasetae on the mesal margin of each paramere.

**Figures 6–9.**
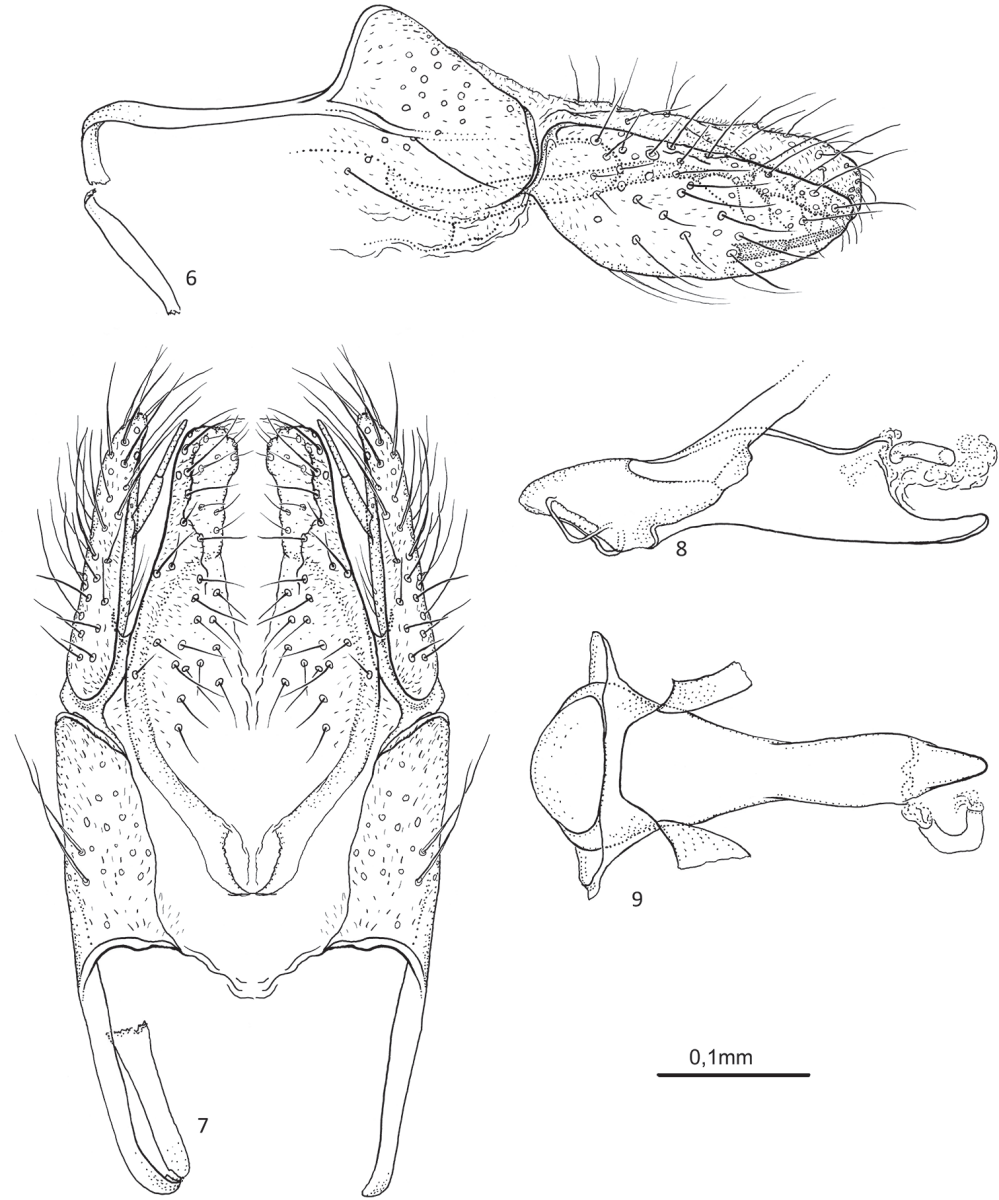
*Agmina semiovale* sp. nov. male holotype **6** genitalia, dorsal part in left lateral view **7** genitalia, dorsal view **8** phallus, lateral view **9** phallus, ventral view.

##### Etymology.

*Semiovale*, from Latin, meaning half oval, named for the shape of the superior appendage in lateral view.

##### Material examined.

***Holotype***: New Caledonia – **Province Sud** • ♂; stream crossing Nouméa-Yaté road immediately W of turnoff to Rivière Bleue Reserve; 22°10.191'S, 166°44.474'E; 162 m; 22.xi-4.xii.2003; Malaise trap; loc#040; leg. KA Johanson; MNHN.

##### Measurements.

Fore wing length 3.6 mm (*N* = 1). Total length of genitalia: 0.5 mm.

##### Description.

***Genitalia***: Segment IX damaged, with sternal processes missing. Tergum X sub-rectangular; in dorsal view, mesally separate, more than two times longer than wide. Parameres robust, starting before tergum X, in lateral view long, wide at base, first half narrowing towards apex, apical half slightly widening, ventral margin slightly concave; in dorsal view, fused at base, apical 2/3^rds^ separated, each with truncated apex, lateral margins convex, mesal margins slightly concave. Superior appendages, in lateral view, longer than tergum X, longer than wide, narrowing along its length, dorsal margin straight, ventral margin convex, with posterad spine-like mesal process present on ventromesal margin not exceeding posteriorly the main branch; in dorsal view slightly narrowing towards apex, running almost parallel, almost straight with blunt apex slightly curving inwards. Mesal processes straight, orientated slightly mesally. Inferior appendages missing due to damage. Phallus, in lateral view approx. as long as segment IX, three times longer than wide; in ventral view proximal half tapering along its length, apical half straight and tapering at apex.

##### Additional information.

This species was referred to as “sp. 57” in Espeland and Johanson (2010a).

#### Agmina rocheta sp. nov.

Taxon classificationAnimaliaTrichopteraEcnomidae

2470C312-10E5-51FF-B026-02AA01BC4DC5

http://zoobank.org/02A8CFE3-5A96-47D9-BA0D-1434230634DA

Figs 10–14

##### Diagnosis.

*Agmina rocheta* sp. nov. is distinguished from the other *Agmina* species by the presence of a pair of drop-shaped superior appendages, in lateral view, each with a very long and curved mesal process reaching as far posteriorly and above the apex of the superior appendages. It particularly resembles *A. nodosa* Ward, 2003 but in *A. nodosa* the sternal processes are simple while in *A. rocheta* sp. nov. they are bifurcated.

**Figures 10–14.**
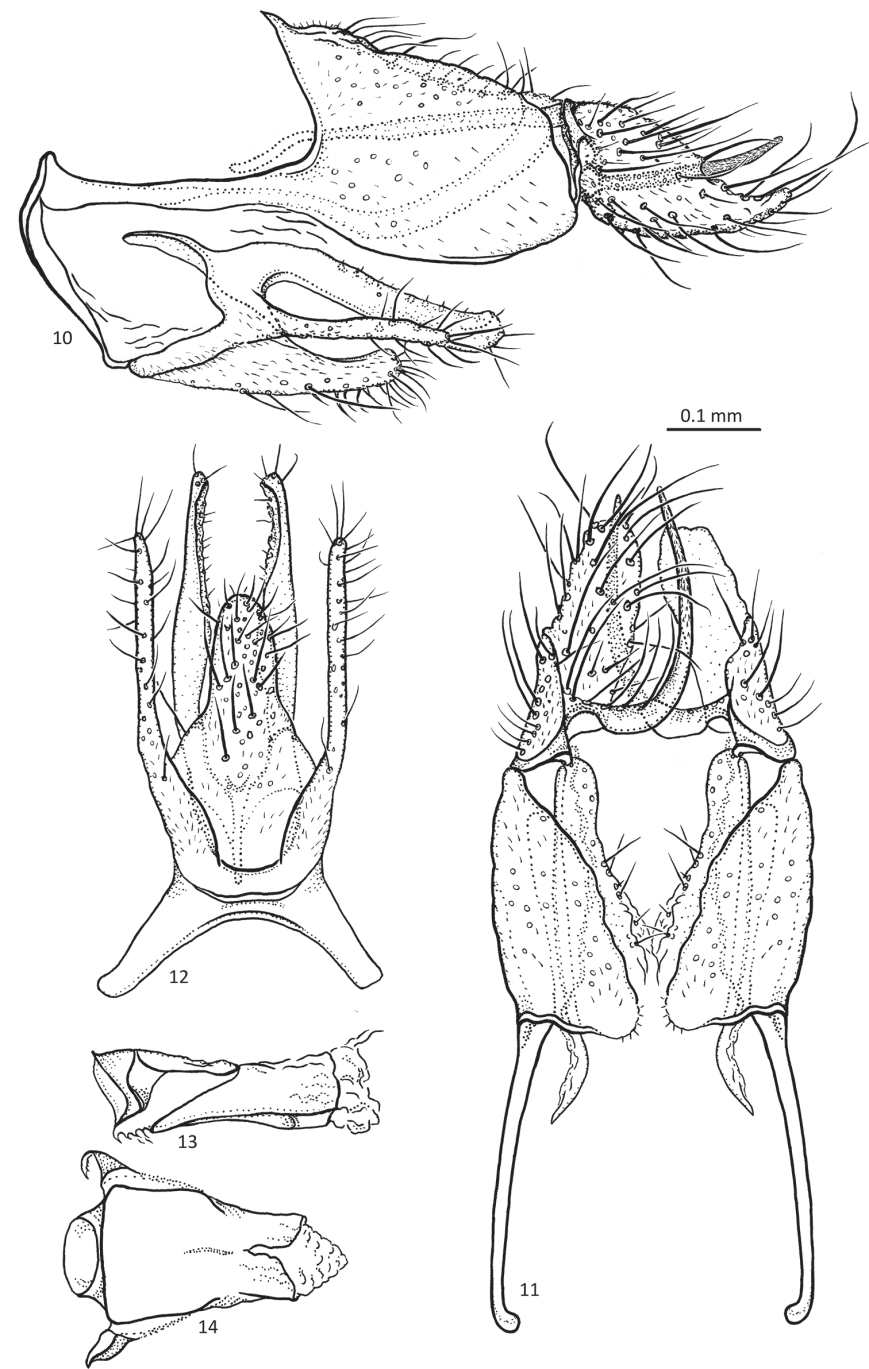
*Agmina rocheta* sp. nov. male holotype **10** genitalia, left lateral view **11** genitalia, dorsal view **12** genitalia, ventral view **13** phallus, lateral view **14** phallus, ventral view.

##### Etymology.

*Rocheta* (noun, feminine), from Latin, meaning rocket. Named for the rocket-shaped phallus in ventral view.

##### Material examined.

***Holotype***: New Caledonia – **Province Nord** • ♂; Wan Pwé On Stream, draining NNE side of Mt. Panié, 3.9 km NW Cascade de Tao; 20°31.820'S, 164°47.016'E; 18.xii.2003; light trap; loc#085; leg. KA Johanson; MNHN.

***Paratype***: New Caledonia – **Province Nord** • 1 ♂; same data as for holotype; NHRS.

##### Type locality.

New Caledonia, Province Nord, Mt. Panié.

##### Measurements.

Fore wing length 4.1–4.3 mm (*N* = 2). Total length of genitalia: 0.4 mm.

##### Description.

***Genitalia***: In lateral view, segment IX apex located medially, dorsal margin straight, abruptly turning downwards at apex, anterior margin convex; in ventral view anteriorly widely U-shaped. Sternal processes, lateral view, with each apex not exceeding posterior apex of tergum X, slender, apical 2/3 straight, spine-like with blunt end; in ventral view, slender, straight, apical 2/3^rd^ parallel. Tergum X very large, in lateral view longer than superior appendage, dorsal margin almost straight, ventral margin convex; in dorsal view, mesally separate, semi-triangular, tapering posteriorly. Parameres starting before tergum X; in lateral view, long, slender, not exceeding tergum X, apical half curving upwards; in dorsal view, fused at base, apical 2/3^rd^ separated, each with truncated apex, narrowing along their length, lateral margins straight, mesal margins slightly concave. Superior appendages, in lateral view, shorter than tergum X, tapering along its length, apex slightly curved upwards, dorsal and ventral margin convex, with posterad, long, spine-like mesal process present on meso-ventral margin exceeding posteriorly the main branch; in dorsal view almost three times longer than wide, converging posteriorly, apex truncated. Mesal processes long, directed mesad at base, crossing each other, then strongly curving posteriorly. Inferior appendages, in lateral view, with posterad orientated, long, tubular dorsal branch at base slightly curving ventrally, then straight, apex blunt; tubular ventral branch 2/3^rd^ the length of dorsal branch, slightly curving upward towards dorsal branch, apex blunt. in ventral view broader at base, with long, slender, almost straight posteriorly orientated dorsal branches, ventral branch forming long central lobe, slightly tapering towards rounded apex. Phallus, in lateral view much shorter than segment IX, straight; in ventral view rocket-shaped.

##### Additional information.

This species was referred to as “sp. 48” in Espeland and Johanson (2010a).

#### Agmina tenuisa sp. nov.

Taxon classificationAnimaliaTrichopteraEcnomidae

347E46B8-CC2D-5A6C-BA34-48FC9401B3BA

http://zoobank.org/C5BBC458-D026-4B39-83B5-EECC7236D03C

Figs 15–19

##### Diagnosis.

*Agmina tenuisa* sp. nov. is distinguished from the other *Agmina* species by the presence of a pair of slender superior appendages, each with a long and slightly undulating mesal process reaching as far posteriorly and on the same height as the apex of the superior appendages; and the presence of a pair of parameres, each ending in the superior appendages and angling ventrally with apical part orientated ventrally below the superior appendages. The genitalia of *Agmina tenuisa* sp. nov. resemble those of *Agmina padi* Ward & Schefter, 2000, particularly in the shape of the inferior appendages in lateral view. *Agmina tenuisa* sp. nov. is distinguished from *A. padi* by the more slender superior appendages in lateral view, the ventral branch of the parameres exceeding below the superior appendages, and in ventral view the plate of inferior appendages has no central posterior plate.

**Figures 15–19.**
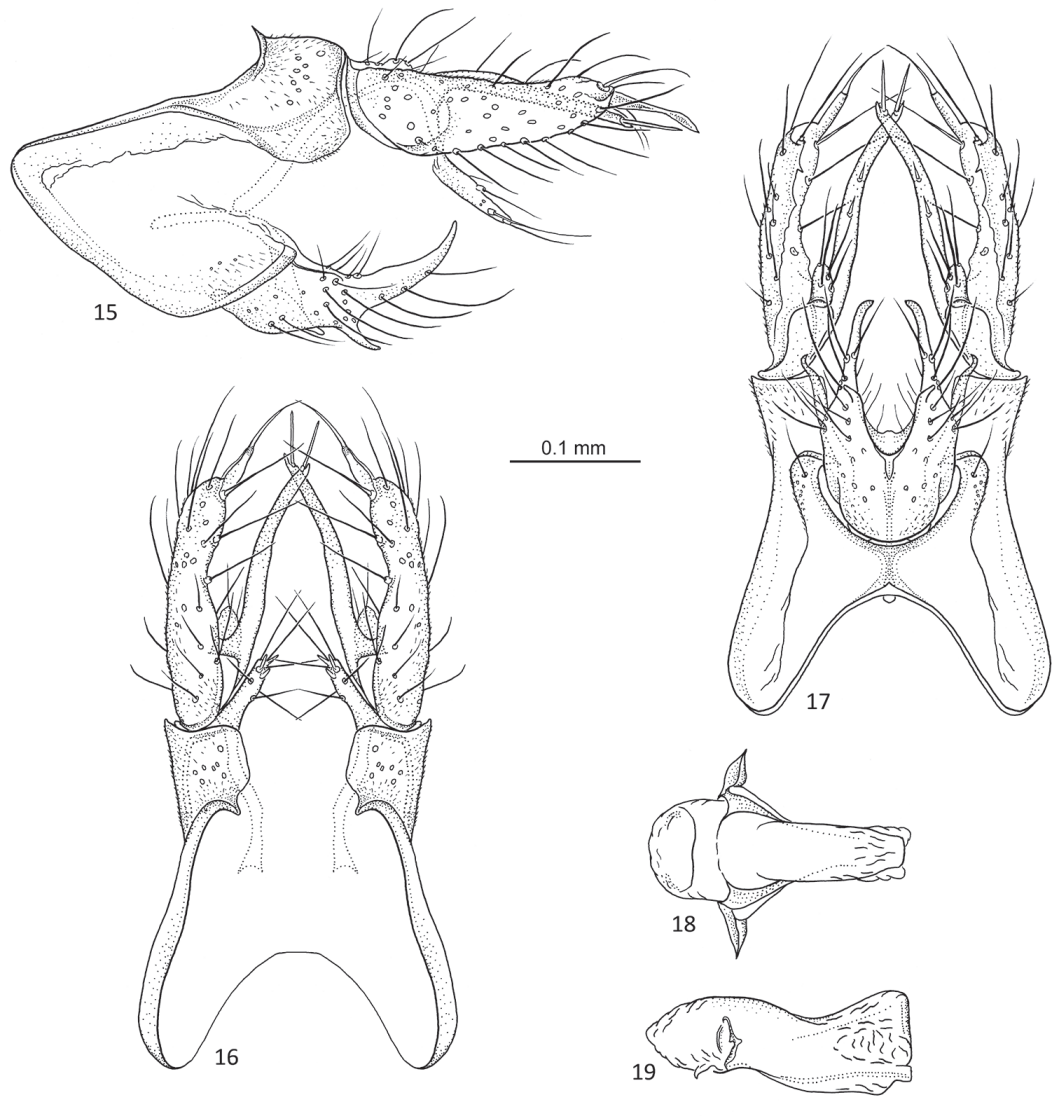
*Agmina tenuisa* sp. nov. male holotype **15** genitalia, left lateral view **16** genitalia, dorsal view **17** genitalia, ventral view **18** phallus, lateral view **19** phallus, ventral view.

##### Etymology.

*Tenuisa*, from Latin, meaning slender. Named for the slender superior appendages as seen in lateral view.

##### Material examined.

***Holotype***: New Caledonia – **Province Sud** • ♂; Haute Yaté fauna reserve, 1760 m S bridge Pont Perignon, 50 m upstream bridge over stream; 22.14954S, 166.701211E; 180 m; 14.xii.2003–13.i.2004; Malaise trap; loc#081; leg. KA Johanson; MNHN.

***Paratypes***: New Caledonia – **Province Sud** • 1 ♂; stream crossing way to Sanatorium 2.3 km E St. Laurent, ca. 30 m downstream bridge; 22°04.484'S, 166°19.900'E; 15.xi.2003; light trap; loc#028; leg. KA Johanson; NHRS; 1 ♂; Koghi Mt., 522 m, source of Riv. Oueanoue; 22°10.327'S, 166°30.524'E; Malaise trap; 12–16.xi.2001; loc 138 (01-2001); leg. KA Johanson, T Pape & B Viklund; NHRS; **Province Nord** • 1 ♂; Mt Mé Amélié, River Fö Töpliba, upstream bridge on rd Sarraméa-Koh, at banana plantation; 21°37.940'S, 165°49.619'E; loc 144 (14-2001); Malaise trap; 18–21.xi.2001; leg. KA Johanson, T Pape & B Viklund; NHRS.

##### Measurements.

Fore wing length 2.9–3.4 mm (*N* = 4). Total length of genitalia: 0.5 mm.

##### Description.

***Genitalia***: In lateral view, segment IX triangular anteriorly, apex located medially; in ventral view anteriorly U-shaped. Sternal processes, lateral view, with each apex not exceeding posterior margin of tergum X, short, triangular; in ventral view, short, not reaching mid-length of inferior appendage, slightly diverging along their length, apices slightly curving mesad. Tergum X small, in lateral view around half the length of inferior appendage, dorsal margin straight, ventral margin slightly convex; in dorsal view, mesally separate, semi-trapezoid, outer margins straight, inner margin slightly convex. Parameres starting at anterior margin of tergum X; in lateral view, long, slender, widely U-shaped, with posterior end slightly diverging, apex pointing posteroventrally; in dorsal view, separated, narrow, posterior 1/3 directed posteromesad, each with short, thick, setae at apex. Superior appendages, in lateral view, slender, slightly curving upwards, more than twice as long as tergum X, with spine-like process at apex, mesal process spine-like straight, slightly curving downwards at base, exceeding posteriorly the main branch; in dorsal view around four times longer than wide, almost parallel, each slightly curving inwards toward apex with thin posteromesad orientated process with long thick seta. Mesal processes long, slender, gently curving mesad, crossing each other near apex. Inferior appendages, in lateral view, with posterodorsally orientated claw-shaped dorsal branch, ventral branch short, claw-shaped, orientated posterodorsally, originating at approx. mid-length on ventral margin of dorsal branch; in ventral view small, oval at base with slender, posteriorly orientated dorsal branches, slightly converging towards apex, ventral branches directed posterolaterally, each with claw-shaped apex. Phallus, in lateral view as long as segment IX, slightly sigmoid; in ventral view anterior half distinctly wider than posterior half.

##### Additional information.

This species was referred to as “sp. 12” in Espeland and Johanson (2010a).

#### Agmina multidentata sp. nov.

Taxon classificationAnimaliaTrichopteraEcnomidae

EEFFCA92-5092-5837-93E2-2670BEE77A31

http://zoobank.org/4EF4D313-1216-4E31-98AF-FFD90E150F95

Figs 20–24

##### Diagnosis.

*Agmina multidentata* sp. nov. is distinguished from the other *Agmina* species by the presence of a pair of uniformly tapering, long superior appendages having deeply undulating ventral and mesal margins along their length; the inferior appendages forming rhomboid plates in lateral view; and tergum X forms a transverse bridge. It resembles *A. cornuta* sp. nov. in the shape of tergum X, but in *A. multidentata* sp. nov. the inferior appendages are higher in lateral view and the superior appendages are more prolonged posteriorly compared to those in *A. cornuta* sp. nov.

**Figures 20–24.**
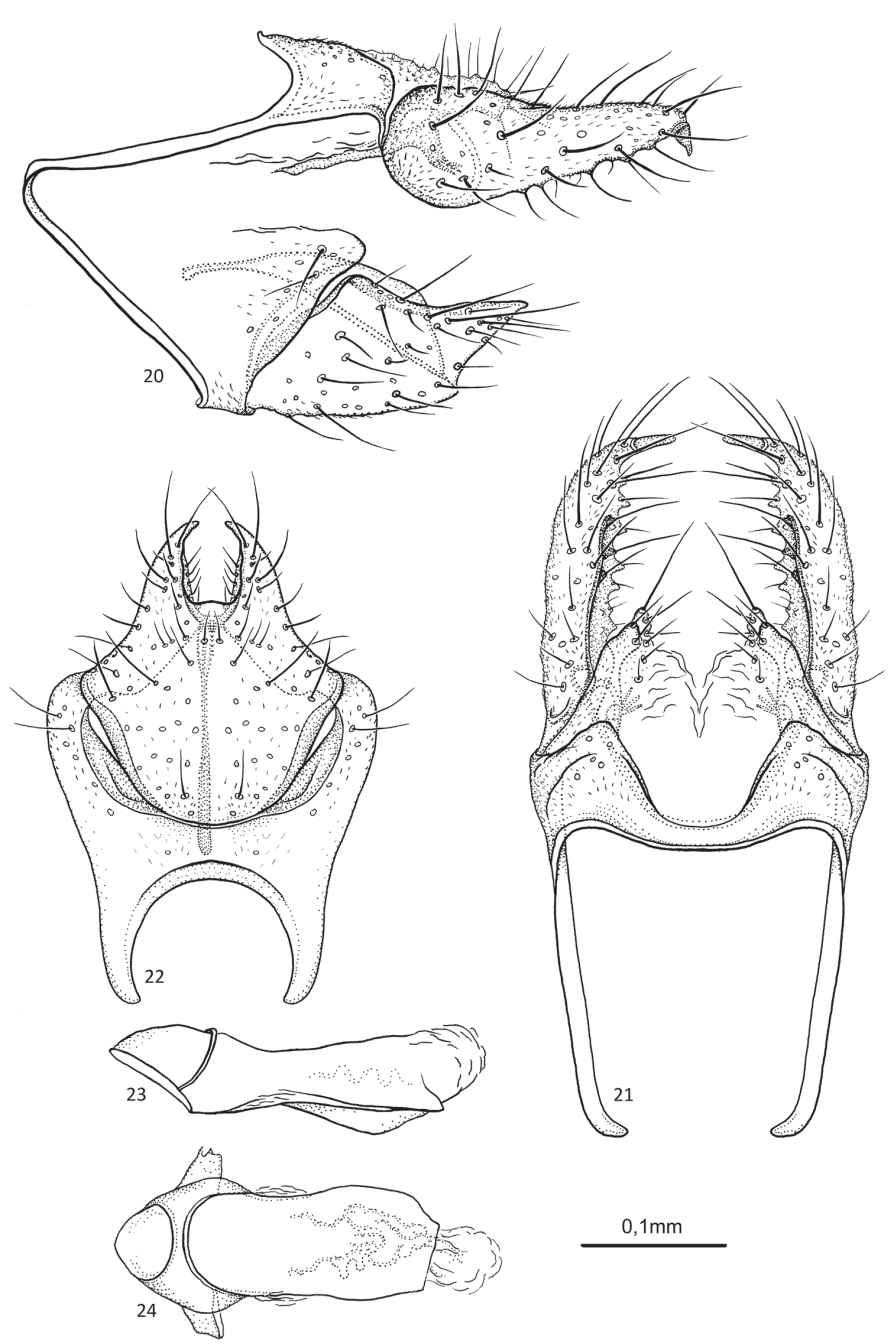
*Agmina multidentata* sp. nov. male holotype **20** genitalia, left lateral view **21** genitalia, dorsal view **22** genitalia, ventral view **23** phallus, lateral view **24** phallus, ventral view.

##### Etymology.

*Multidentata*, from Latin, meaning many teeth. Named for the ventral and mesal margins of the superior appendages with many teeth as seen in lateral view.

##### Material examined.

***Holotype***: New Caledonia – **Province Sud** • ♂; Réserve spéciale de faune de la haute Yaté, along road on southern part of Marais de la Rivière Blanche, stream draining to Marais de la Rivière Blanche, 5.0 km SW Pont Pérignon; 22°09.513'S, 166°39.942'E; 180 m; 6.xi-16.xi.2003; Malaise trap; loc#011; leg. KA Johanson; MNHN.

***Paratypes***: New Caledonia – **Province Sud • 2** ♂; W part of Plaine des lacs, 150 m downstream bridge at La Capture; 22°15.967'S, 166°49.493'E; 261 m; 4–22.xi.2003; Malaise trap; loc#007; leg. KA Johanson; NHRS; • 1 ♂; stream crossing Nouméa-Yaté road, 1.5 km S Yaté Dam, approx. 200 m upstream the road; 22°09.931'S, 166°52.535'E; 197 m; 22.xi-17.xii.2003; Malaise trap; loc#041; leg. KA Johanson; NHRS.

##### Measurements.

Fore wing length 2.8–3.6 mm (*N* = 4). Total length of genitalia: 0.5 mm.

##### Description.

***Genitalia***: In lateral view, segment IX triangular anteriorly, apex located mediodorsally; in ventral view anteriorly semi-circular. Sternal processes, lateral view, with each rounded apex not exceeding posterior apex of tergum X, triangular; in ventral view, broad, reaching mid-length of inferior appendage, slightly diverging, apices curving mesad. Tergum X small, in lateral view less than half the length of superior appendage, slightly longer than high, inner and outer margins almost straight, slightly diverging anteriorly, anterior margin concave; in dorsal view, mesally connected, axe-shaped. Parameres complex; in lateral view with tubular dorsal lobe running parallel with dorsal margin of tergum X and superior appendage, ending before mid-length of superior appendage; shorter, narrower, ventral lobe orientated posteroventrally; in dorsal view, dorsal lobes separated and narrowing along their length, not reaching mid-length of superior appendage, directed posteromesad, each with truncate apex. Superior appendages, in lateral view, more than twice as long as tergum X, tapering along its length with rounded base, apex semi-acute with stout megaseta orientated ventrad mesal process absent; in dorsal view uniformly narrow, running almost parallel, slightly curving inwards towards apex with stout megaseta directed mesad, inner margin dentate. Mesal processes absent. Inferior appendages, in lateral view, with posterad orientated triangular dorsal branch with acute apex; dorsal branch present as short, obtuse process at posteroventral margin in ventral view convex at base, gradually widening until mid-length, then tapering towards apex, dorsal branches orientated posteriorly, claw-shaped, with apices directed posteromesad, ventral branch barely visible as short very obtuse central lobe. Phallus, in lateral view as long as segment IX, almost straight with slightly concave margins in ventral view anterior half not distinctly wider than posterior half.

##### Additional information.

This species was referred to as “sp. 10” in Espeland and Johanson (2010a).

#### Agmina cornuta sp. nov.

Taxon classificationAnimaliaTrichopteraEcnomidae

8DF29A39-4D9C-517F-8D8B-CC67E8B1A4E5

http://zoobank.org/F11DF372-A11F-4D40-AC2A-2625787E0DF3

Figs 25–30

##### Diagnosis.

*Agmina cornuta* sp. nov. is distinguished from the other *Agmina* species by the presence of a pair of almost uniformly oval superior appendages in lateral view, and a pair of branches of the inferior appendages which appear beak-like in lateral view and are approx. as long as the superior appendages. It resembles *A. multidentata* sp. nov. in the shape of tergum X, but in *A. cornuta* sp. nov. the inferior appendages are lower in lateral view and the superior appendages are rounder posteriorly compared to those in *A. multidentata* sp. nov.

**Figures 25–30.**
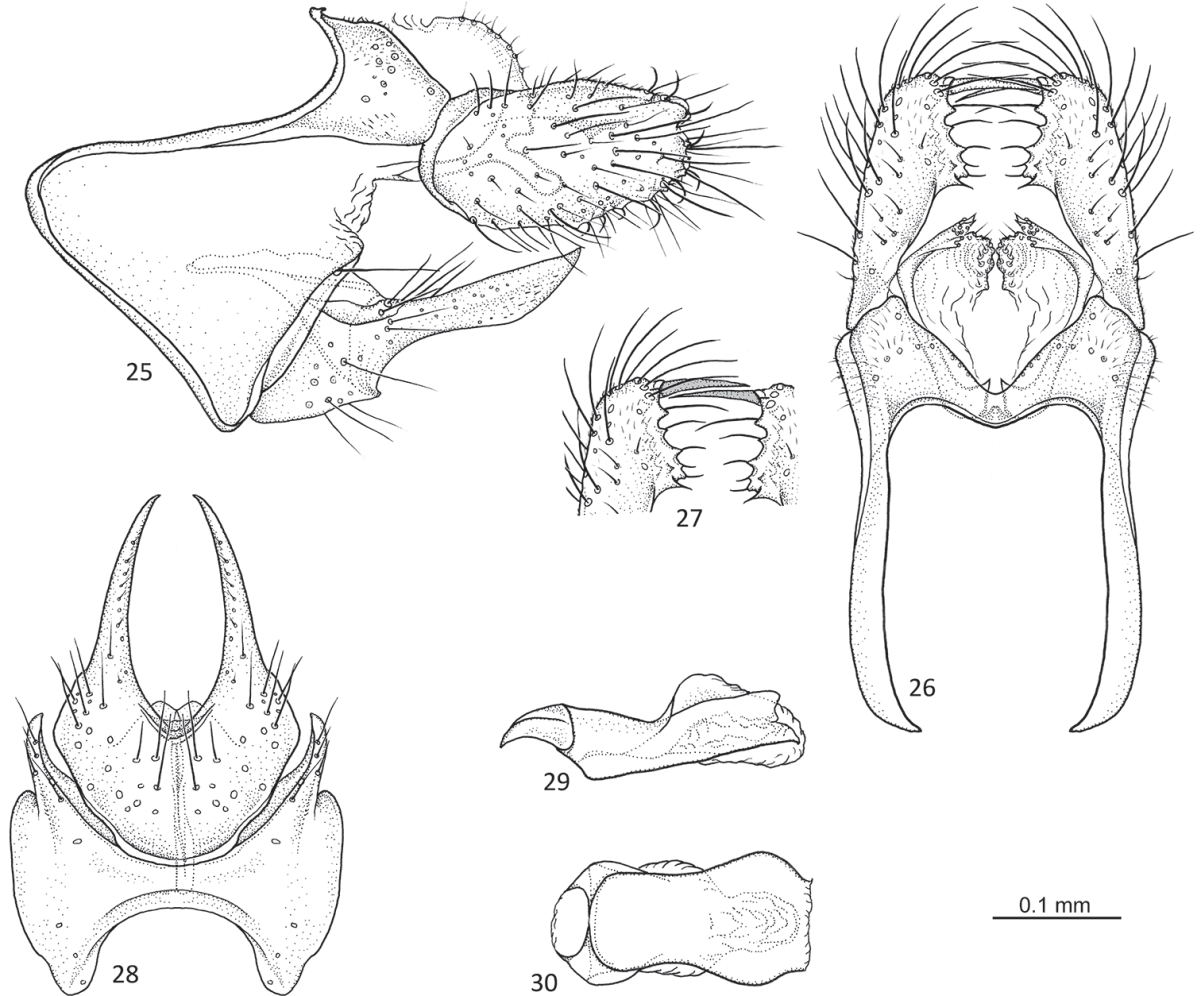
*Agmina cornuta* sp. nov. male holotype **25** genitalia, left lateral view **26** genitalia, dorsal view **27** end of superior appendages, showing mesal spine, dorsal view **28** genitalia, ventral view **29** phallus, lateral view **30** phallus, ventral view.

##### Etymology.

*Cornuta*, from Latin, meaning horned. Named for the shape of the inferior appendage in lateral view, which has two long, horn-shaped, dorsal branches.

##### Material examined.

***Holotype***: New Caledonia – **Province Sud** • ♂; Plateau de Dogny, northern part; 21°36.853'S, 165°52.548'E; loc#159 (32-2001); Malaise trap; 2–5.xii-2001; leg. KA Johanson, T Pape & B Viklund. MNHN.

***Paratypes***: New Caledonia – **Province Sud** • ♂; Réserve spéciale de faune de la haute Yaté, along road on southern part of Marais de la Rivière Blanche, stream draining to Marais de la Rivière Blanche, 2.25 km SW Pont Pérignon; 180 m; 6–16.xi.2003; Malaise trap; loc#010a; leg. KA Johanson; NHRS; • ♂; Mt. Dzumac, source stream of Ouinne River, near crossing point to mountain track; 22°02.073'S, 166°28.460'E; 810 m; 18.xi-4.xii.2003; Malaise trap; loc#030; leg. KA Johanson; NHRS; • ♂; Monts des Koghis, ca 300 m S Koghi Restaurant; 22.18288S, 166.50167E; 417 m; 2–16.xi.2003; Malaise trap; loc#004; leg. KA Johanson; NHRS.

##### Measurements.

Fore wing length 3.2–4.1 mm (*N* = 4). Total length of genitalia: 0.5 mm.

##### Description.

***Genitalia***: In lateral view, segment IX semi-triangular anteriorly, apex located dorsally; in ventral view anteriorly widely U-shaped. Sternal processes, lateral view, with each apex, not exceeding posterior apex of tergum X, very short, triangular; in ventral view, short, slender, not reaching mid-length of inferior appendage, each with apex slightly curving mesad. Tergum X small, in lateral view approx. half the length of superior appendage, outer margin irregularly convex, slightly converging towards posterior with straight inner margin; in dorsal view, mesally connected, axe-shaped with inner margins forming a right triangle. Parameres starting at tergum X, in lateral view semi-circular, each with narrow protrusion orientated posteroventrad with blunt apex, approx. half the length of the main parameres; in dorsal view, separated, slightly diverging towards apices, outer margins straight for first 2/3, then abruptly curving inwards at obtuse angle, inner margin irregularly lobed. Superior appendages, in lateral view, approx. twice as long as tergum X, semi-ovoid with truncated, irregularly dentate apex, mesal process absent; in dorsal view almost parallel, each gently widening to mid-length, then inner margin abruptly widening, with apical half irregularly dentate. Mesal processes absent. Inferior appendages, in lateral view, with posterad orientated long, knife-shaped dorsal branch; ventral branch present as short, obtuse process at posteroventral margin; in ventral view convex at base, gradually widening for first 1/3, then abruptly narrowing towards apex, long, narrow, slightly tapering dorsal branches orientated posteriorly, slightly curving inwards, apices pointed, ventral branch indiscernible. Phallus, in lateral view shorter than segment IX, irregular; in ventral view posterior half slightly wider than anterior half.

##### Additional information.

This species was referred to as “sp. 53” in Espeland and Johanson (2010a).

### Species group 2, *sagittata* group

This group includes the following species: *Agmina sagittata* sp. nov., *A. circulata* sp. nov., *A. jepiva* Ward & Schefter, 2000, *A. digitata* sp. nov., *A. longispina* sp. nov., *A. urugi* Ward & Schefter, 2000, *A. magnahamata* sp. nov., *A. longicordata* sp. nov., *A. campanula* sp. nov., *A. semicampanula* sp. nov., *A. cunicula* sp. nov., *A. panda* Ward & Schefter, 2000, *A. cerritula* sp. nov., *A. monstrosa* sp. nov., *A. rectangulata* sp. nov., *A. chela* sp. nov., *A. mariae* Ward & Schefter, 2000, and *A. piscaria* sp. nov.

#### Agmina sagittata sp. nov.

Taxon classificationAnimaliaTrichopteraEcnomidae

FB9D82B1-C807-50F6-860D-60E169D04ADF

http://zoobank.org/A1558F49-EE6D-492A-83F3-CFE38A48B280

Figs 31–35

##### Diagnosis.

*Agmina sagittata* sp. nov. is distinguished from the other *Agmina* species in the genitalia by the presence of a pair of inferior appendages having a ventral part that, in lateral view, reaches as far posteriorly as the sternal processes, and the apex of the sternal processes and inferior appendages are separated by a rectangular incision. It resembles *A. circulata* sp. nov. in the overall similarity of the genitalia, but *A. circulata* sp. nov. is larger than *A. sagittata* sp. nov., and in the genitalia *A. sagittata* sp. nov. has superior appendages that are more rounded in lateral view, shorter sternal processes in relation to the inferior appendages, and the sternal processes of *A. sagittata* sp. nov. is slightly wider and straighter than those of *A. circulata* sp. nov.

**Figures 31–35.**
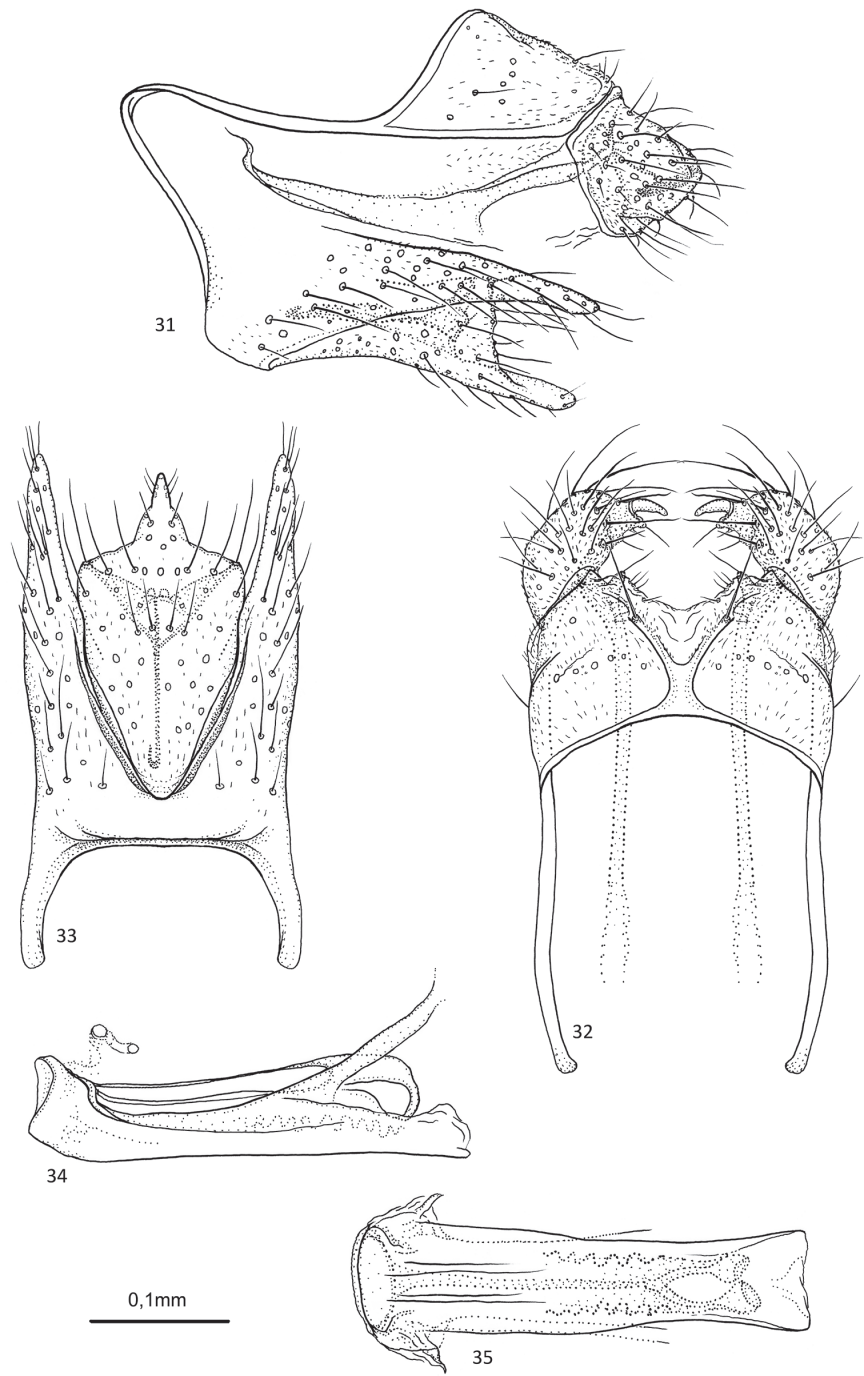
*Agmina sagittata* sp. nov. male holotype **31** genitalia, left lateral view **32** genitalia, dorsal view **33** genitalia, ventral view **34** phallus, lateral view **35** phallus, ventral view.

##### Etymology.

*Sagittata*, from Latin, meaning arrow-shaped. Named for the inferior appendages being arrow-shaped in ventral view.

##### Material examined.

***Holotype***: New Caledonia – **Province Nord** • ♂; Aoupinié Mt., Réserve spéciale de faune de l’Aoupinié, spring to side stream to Öröpömwati River; 21°08.386'S, 165°19.257'E; 402 m; 6–27.xii.2003; Malaise trap; loc#064; leg. KA Johanson; MNHN.

***Paratypes***: New Caledonia – **Province Nord** • 1 ♂; Réserve spéciale de faune de l’Aoupinié, ca 25 km S Poindimié, 21°08.940'S, 165°19.409'E, loc#147a (17-2001), Malaise trap; 24–28.xi.2001; leg. KA Johanson, T Pape & B Viklund; NHRS; • 1 ♂; ditto, except 21°09.775'S, 165°19.017'E; loc#148 (18-2001); Malaise trap; 24–28.xi.2001; leg. KA Johanson, T Pape & B Viklund; NHRS;

• 2 ♂; ditto, except 21°09.369'S, 165°19.209'E; loc#149 (19-2001); Malaise trap; 24–28.xi.2001; leg. KA Johanson, T Pape & B Viklund; NHRS; **Province Sud** • ♂; Monts Kwa Ne Mwa, along Nouméa-Yaté road, 2.0 km E Pic Mouirange, 20 m upstream road; 22°12.356'S, 166°40.798'E; 220 m; 15–16.i.2004; light trap; loc#120; leg. KA Johanson; NHRS; • ♂; Réserve spéciale de faune de la haute Yaté, along road on southern part of Marais de la Rivière Blanche, stream draining to Marais de la Rivière Blanche, 3.7 km SW Pont Pérignon; 22°09.327'S, 166°40.841'E; 180 m; 6–16.xi.2003; Malaise trap; loc#013; leg. KA Johanson; NHRS; • 1 ♂; Mt. Panié, Riv. Padyéém, 400 m, 22–28.xi.2001, Malaise trap 22–28.xi.2001, 20°34.122'S, 164°48.147'E, loc 146 (16-2001); leg. KA Johanson, T Pape & B Viklund; NHRS.

##### Measurements.

Fore wing length 3.2–4.1 mm (*N* = 8). Total length of genitalia: 0.5 mm.

##### Description.

***Genitalia***: Total length 0.4 mm. In lateral view, segment IX semi-triangular anteriorly, apex located dorsally; in ventral view anteriorly obround. Sternal processes, in lateral view, with each apex exceeding the length of tergum X, elongated narrowly triangular with semi-acute apex, dorsal margin almost straight, ventral margin gently concave; in ventral view, longer than inferior appendage, narrowing along their length, apex acute. Tergum X in lateral view irregularly quadrilateral, slightly longer than superior appendage, longer than wide, tapering along its length; in dorsal view, mesally separate, axe-shaped, inner margins forming triangle. Parameres starting before tergum X, in lateral view long, slowly widening along 2/3 of its length, then narrow, apex slender, multifurcated, ending before apex of superior appendage; in dorsal view, separated, long, slender, straight, apex pipe wrench-shaped. Superior appendages, in lateral view, shorter than tergum X, semi-trapezoid; in dorsal view stout, curving inwards, with rounded outer margin; mesal process, stout claw-like, directed mesad. Inferior appendages, in lateral view, with posterad orientated short, rectangular dorsal branch; ventral branch narrow, tubular with rounded apex, as long as sternal process; in ventral view sagittate, pointing anteriorly. Phallus, in lateral view shorter than segment IX, complex, ventral margin straight; in ventral view tubular, anterior end slightly wider than remainder.

##### Additional information.

This species was referred to as “sp. 21” in Espeland and Johanson (2010a).

#### Agmina circulata sp. nov.

Taxon classificationAnimaliaTrichopteraEcnomidae

FF12235A-FC27-5942-8316-0B14E9BF4706

http://zoobank.org/5412025A-3150-4520-B40E-1232535326C5

Figs 36–40

##### Diagnosis.

*Agmina circulata* sp. nov. is distinguished from the other *Agmina* species in the genitalia by the presence of a pair of inferior appendages having a ventral part that, in lateral view, almost reaches as far posteriorly as the sternal processes, and with the apex of the sternal processes and inferior appendages that are separated by a rectangular incision. It resembles *A. sagittata* sp. nov. in the overall similarity in the genitalia, but *A. circulata* sp. nov. is smaller than *A. sagittata* sp. nov., and in the genitalia *A. sagittata* sp. nov. has superior appendages that are more rounded in lateral view, shorter sternal processes in relation to the inferior appendages, and the sternal processes of *A. sagittata* sp. nov. is slightly wider and straighter than those of *A. circulata* sp. nov.

**Figures 36–40.**
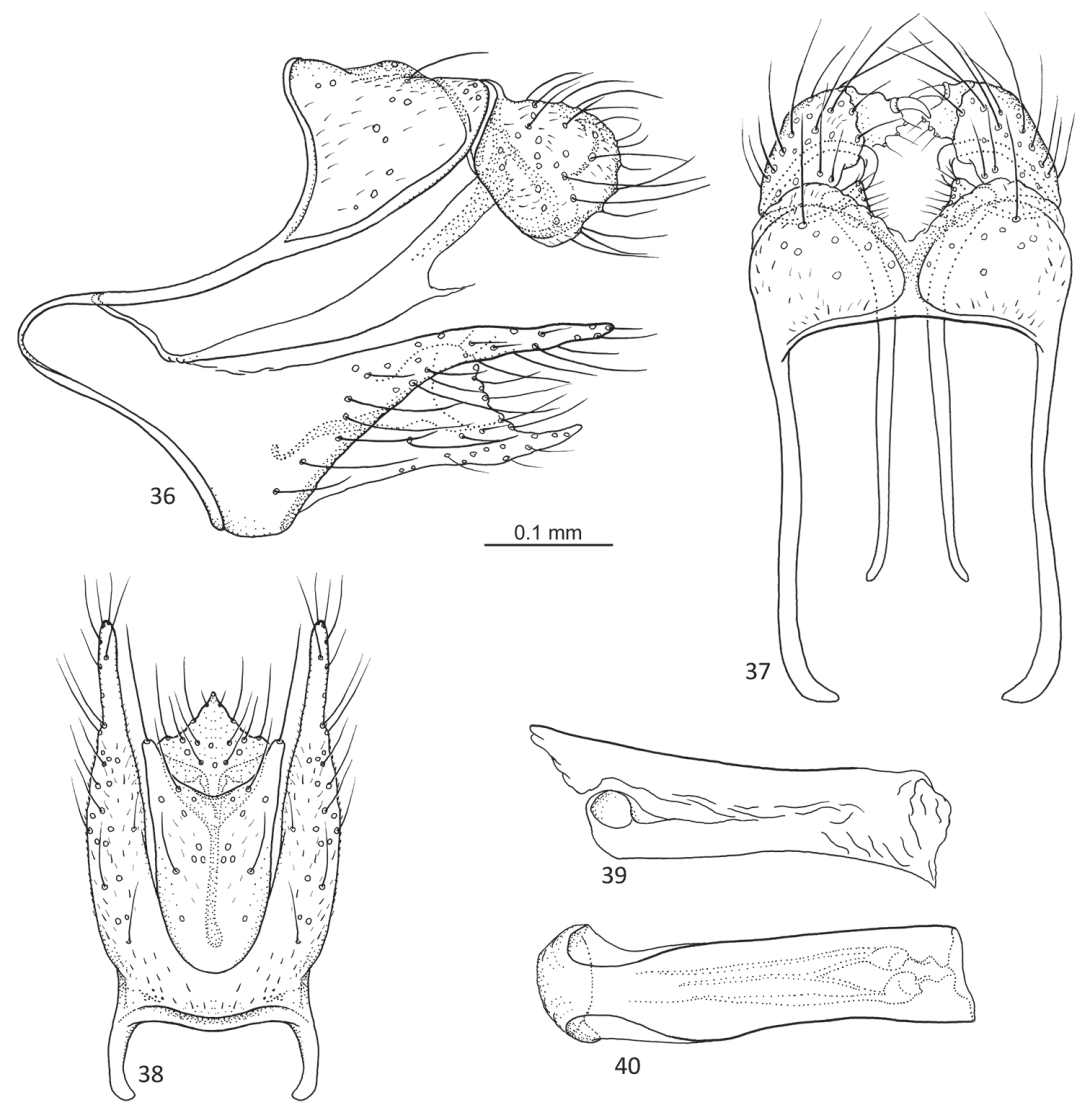
*Agmina circulata* sp. nov. male holotype **36** genitalia, left lateral view **37** genitalia, dorsal view **38** genitalia, ventral view **39** phallus, lateral view **40** phallus, ventral view.

##### Etymology.

*Circulata* from Latin, meaning rounded. Named for the parameres forming a circular loop at the apex.

##### Material examined.

***Holotype***: New Caledonia – **Province Sud** • ♂; W part of Plaine des lacs, 150 m downstream bridge at La Capture; 22°15.967'S, 166°49.493'E; 261 m; 4–22.xi.2003; Malaise trap; loc#007; leg. KA Johanson; MNHN.

***Paratype***: New Caledonia – **Province Nord** • 1 ♂; same data as for holotype, except NHRS.

##### Measurements.

Fore wing length 4.6 mm (*N* = 1). Total length of genitalia: 0.5 mm.

##### Description.

***Genitalia***: In lateral view, segment IX narrowly bell-shaped anteriorly, apex located medially; in ventral view anteriorly concave lens-shaped with slightly rounded lateral margins. Sternal processes, lateral view, with each apex exceeding the length of tergum X and almost superior appendage, elongated narrowly triangular with acute apex, dorsal margin relatively straight, ventral margin gently concave; in ventral view, longer than inferior appendage, narrowing along their length, apex semi-acute, outer margin slightly rounded, inner margin straight. Tergum X in lateral view semi-triangular with concave anterior margin, dorsal margin with central bump, longer than superior appendage, longer than wide; in dorsal view, mesally separate, golf club shaped, inner margins forming shallow triangle. Parameres starting before tergum X, in lateral view long, slowly widening along 2/3 of its length, then narrow, apex forming ring, ending before apex of superior appendage; in dorsal view, separated, long, slender, straight, apex forming ring. Superior appendages, in lateral view, shorter than tergum X, irregularly rounded; in dorsal view stout, slightly curving inwards, with rounded outer margin and gently lobed inner margin, apex directed mesad with claw like process directed posteromesad. Inferior appendages, in lateral view, with posterad orientated short, rounded dorsal branch; ventral branch spine like, gently curving dorsad, almost reaching length of sternal process; in ventral view narrowly rounded base, slightly diverging along its length towards triangular apex. Phallus, in lateral view shorter than segment IX, tubular; in ventral view tubular with rounded anterior end.

##### Additional information.

This species was referred to as “sp. 19” in Espeland and Johanson (2010a).

#### Agmina digitata sp. nov.

Taxon classificationAnimaliaTrichopteraEcnomidae

90DDA974-E5BD-5A27-8A00-28A511DD95F4

http://zoobank.org/E2576FF9-A450-4DEF-9FA2-66B45EC5B7CB

Figs 41–45

##### Diagnosis.

*Agmina digitata* sp. nov. is distinguished from the other *Agmina* species by the combination of a pair of long, straight sternal processes that form almost a right angle with the anterior margin of segment IX in lateral view; the apex almost reaching the posterior terminal part of the superior appendages; inferior appendages well hidden behind the sternal processes in lateral view; and the superior appendages in lateral view being rhomboid and slightly produced dorsally at the posterior end. It resembles *A. cunicula* sp. nov. in the shape of the genitalia in lateral view, but *A. digitata* sp. nov. can be separated from *A. cunicula* sp. nov. by the shape and larger size of the superior appendages.

**Figures 41–45.**
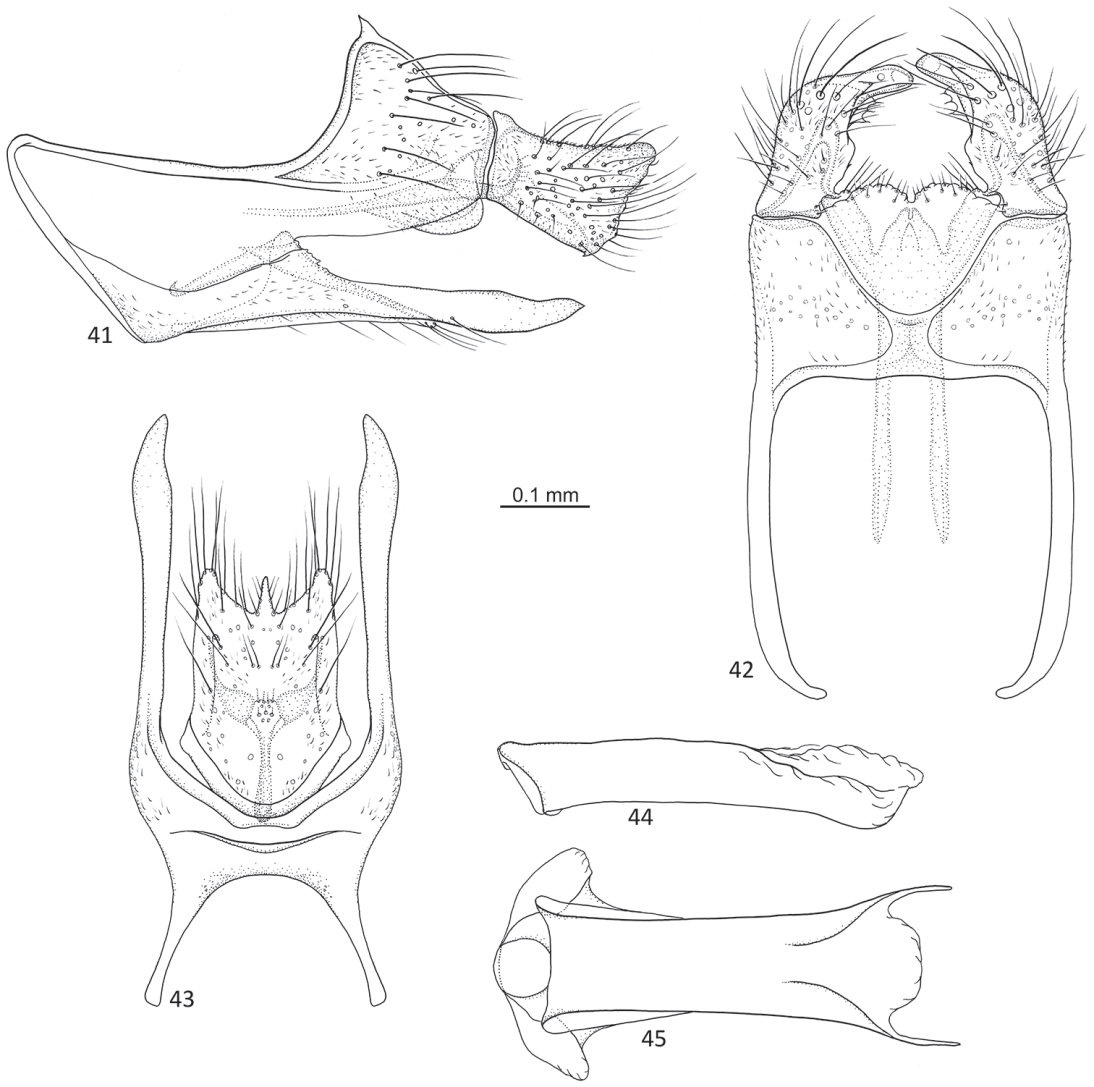
*Agmina digitata* sp. nov. male holotype **41** genitalia, left lateral view **42** genitalia, dorsal view **43** genitalia, ventral view **44** phallus, lateral view **45** phallus, ventral view.

##### Etymology.

*Digitata*, from Latin, meaning digit-like. Named for the shape of the apex of the superior appendage in dorsal view, which is toe-shaped.

##### Material examined.

***Holotype***: New Caledonia – **Province Nord** • ♂; 50 m upstream bridge on Hienghène-Tnèdo road, 3.9 km S summit of Mt. Tnèda, 2.2 km E Tnèdo; 20°43.085'S, 164°49.928'E; 29 m; 7.xii.2003; light trap; loc#071a; leg. KA Johanson; MNHN.

##### Measurements.

Fore wing length 4.0 mm (*N* = 1). Total length of genitalia: 0.7 mm.

##### Description.

***Genitalia***: In lateral view, segment IX triangular anteriorly, apex located medially; in ventral view, anteriorly widely U-shaped. Sternal processes, lateral view, with each apex exceeding the length of tergum X and almost superior appendage, long, slender, dorsal margin with widely triangular notch before mid-length, then straight, ventral margin slightly concave, apex acute; in ventral view, almost twice as long as inferior appendage, long, slender, straight, slightly widening at club shaped, acute apex. Tergum X in lateral view approx. same length as superior appendage, irregularly triangular; in dorsal view, mesally separate, irregularly quadrilateral, rounded antero-mesally, inner margins forming U. Parameres starting before tergum X, in lateral view slender, with two thin whip-like processes at apex almost forming a ring, barely reaching superior appendage; in dorsal view, anteriorly separated, straight, posterior half fused to complex structure, apex with central lobe and truncate lateral branches ending at base of superior appendage. Superior appendages, in lateral view, axe-blade shaped with dorsal margin slightly concave, as long as tergum X; in dorsal view stout, curving inwards, with rounded outer margin and irregular inner margin, apex finger-like, directed mesoposterad. Inferior appendages, in lateral view, with posterad orientated, small, widely triangular dorsal branch; ventral branch narrowly triangular, apex acute; in ventral view rounded at base, margins almost parallel, posterior margin concave with spine-like central process. Phallus, in lateral view shorter than segment IX, tubular; in ventral view tubular, anterior end with rounded lateral lobes, posterior end convex with spine-like lateral processes.

##### Additional information.

This species was referred to as “sp. 50” in Espeland and Johanson (2010a).

#### Agmina longispina sp. nov.

Taxon classificationAnimaliaTrichopteraEcnomidae

DB8585E5-A3D0-5374-93C8-0CFE74A2495F

http://zoobank.org/AB339E71-AF14-4234-A149-31843FAAA773

Figs 46–51

##### Diagnosis.

*Agmina longispina* sp. nov. is unique in having a pair of lower parameres that are needle-like and exceed the superior appendages posteriorly, and in ventral view are orientated laterally before curving slightly mesally. It resembles many other species in the genus having small and almost rectangular or rhomboid superior appendages in lateral view, but is distinguished from these species by the presence of the lower parameres, as well as the straight sternal processes that are undulating in thickness. It resembles *A. longicordata* sp. nov. in the presence of a group of densely arranged setae at the posterior part of the parameres. *Agmina longispina* sp. nov. is easily separated from *A. longicordata* sp. nov. by the more rectangular shape of the inferior appendage plate in ventral view.

**Figures 46–51.**
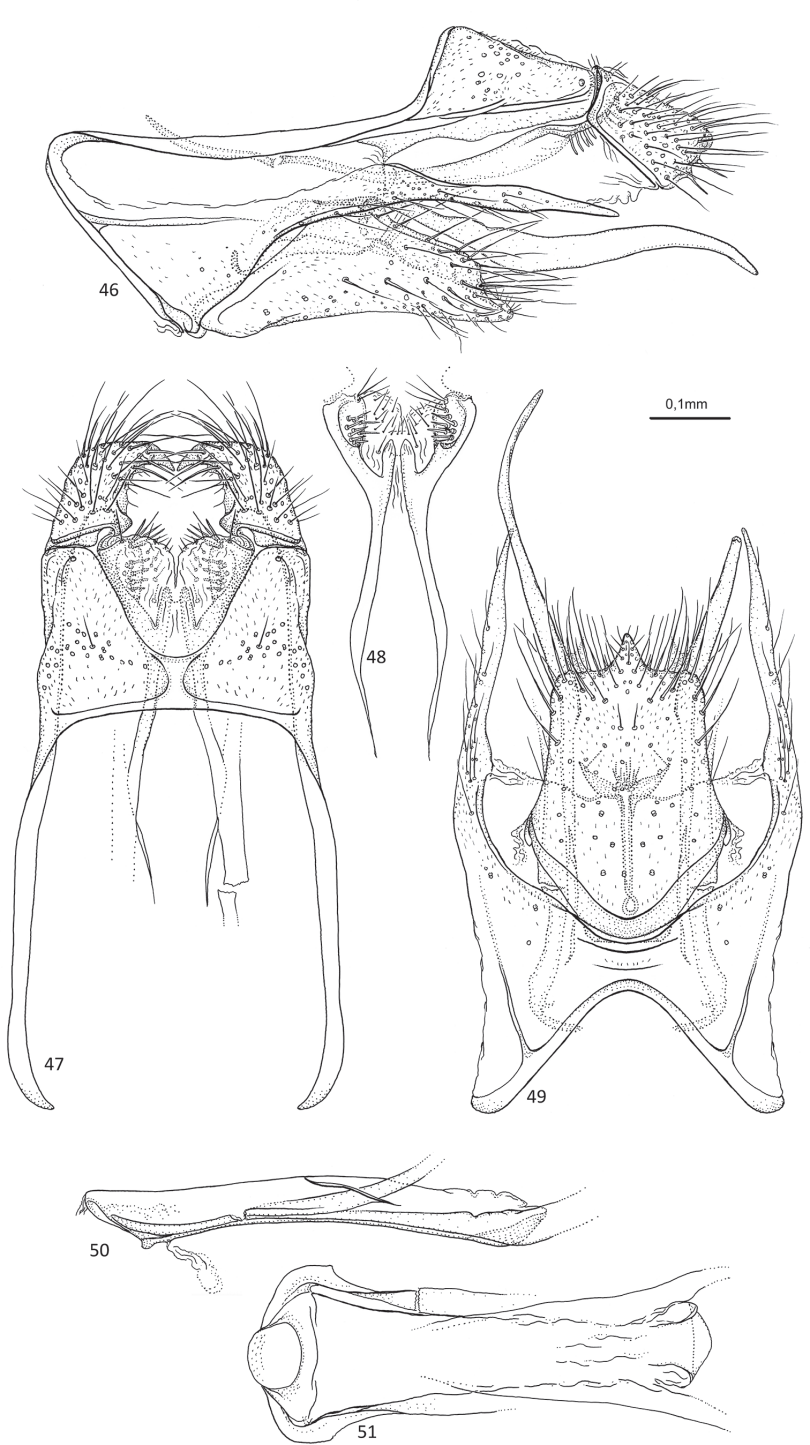
*Agmina longispina* sp. nov. male holotype **46** genitalia, left lateral view **47** genitalia, dorsal view **48** parameres, dorsal view **49** genitalia, ventral view **50** phallus, lateral view **51** phallus, ventral view.

##### Etymology.

*Longispina*, from Latin, *longi* and *spina* (noun, feminine) meaning long spine. Named for the shape of the sternal processes in lateral view, which are long and pointed posteriorly.

##### Material examined.

***Holotype***: New Caledonia – **Province Sud** • ♂; W slope Mt. Ningua, Kwé Néco Stream, at Camp Jacob, 3.7 km WNW summit of Mt. Ningua, on Boulo-Thio Road, ca. 50 m upstream road; 21°43.613'S, 166°06.567'E; 150 m; 29.xi-12.xii.2003; Malaise trap; loc#054; leg. KA Johanson; MNHN.

##### Measurements.

Fore wing length 3.9 mm (*N* = 1). Total length of genitalia: 0.9 mm.

##### Description.

***Genitalia***: In lateral view, segment IX triangular anteriorly; apex located dorsally; in ventral view anteriorly widely bell shaped. Sternal processes, lateral view, with each apex exceeding the length of apex X, almost reaching apex of superior appendage; wide at base, then long, slender, slightly curving ventrad, apex acute; in ventral view, longer than inferior appendage, slender, slightly converging towards acute apex. Tergum X in lateral view trapezoid, tapering along its length, slightly longer than superior appendage, longer than wide; in dorsal view, mesally separate, trapezoid, tapering along its length, inner margins forming bell. Parameres comprising pair of upper and lower branches; upper branches starting before tergum X, reaching base of superior appendage, in lateral view, first half very slender, then slightly broadening along its length, spine-like at apex with multiple wide setae on ventral surface, directed ventrad; in dorsal view, first half separated, slender, gradually converging and fusing at apex forming two large lobes; lower branches starting anteriorly of upper branches, slender and undulating in lateral view, in ventral view with anterior halves orientated posteriorly before bending laterally and slightly recurving mesally before posterior end, posteriorly exceeding superior appendages. Superior appendages, in lateral view, almost rectangular, slightly wider posteriorly, shorter than tergum Xin dorsal view stout, curving inwards, outer margin rounded, inner margin relatively rounded, apex claw-like, directed mesoanteriorly. Inferior appendages, in lateral view, with posterad orientated, slender, acute dorsal branch, exceeding length of superior appendage, ventral branch half the length of dorsal branch, broader, irregularly tapering along its length, abruptly narrowing at semi-acute apex; in ventral view widely bell-shaped at base, margins almost parallel, posterior margin straight with narrowly triangular central process. Phallus, in lateral view shorter than segment IX, slender, tubular, slightly curving downwards; in ventral view largely tubular, straight, slightly wider anteriorly.

##### Additional information.

This species was referred to as “sp. 34” in Espeland and Johanson (2010a).

#### Agmina magnahamata sp. nov.

Taxon classificationAnimaliaTrichopteraEcnomidae

1A25A9C6-BBDF-59BB-A7D5-CDFE602D7A87

http://zoobank.org/22A9C164-3B51-481E-8E55-2F829FA8E1DC

Figs 52–57

##### Diagnosis.

*Agmina magnahamata* sp. nov. resembles many other *Agmina* species having short superior appendages with a large, strong mesally hook-shaped process that are present in other species, particularly in the *Agmina sagittata* species group. *Agmina magnahamata* sp. nov. resembles species with straight and posteriorly wide sternal processes, like *A. digitata* sp. nov. and *A. circulata* sp. nov., from which *A. magnahamata* sp. nov. is distinguished by the more complex parameres in the genitalia.

**Figures 52–57.**
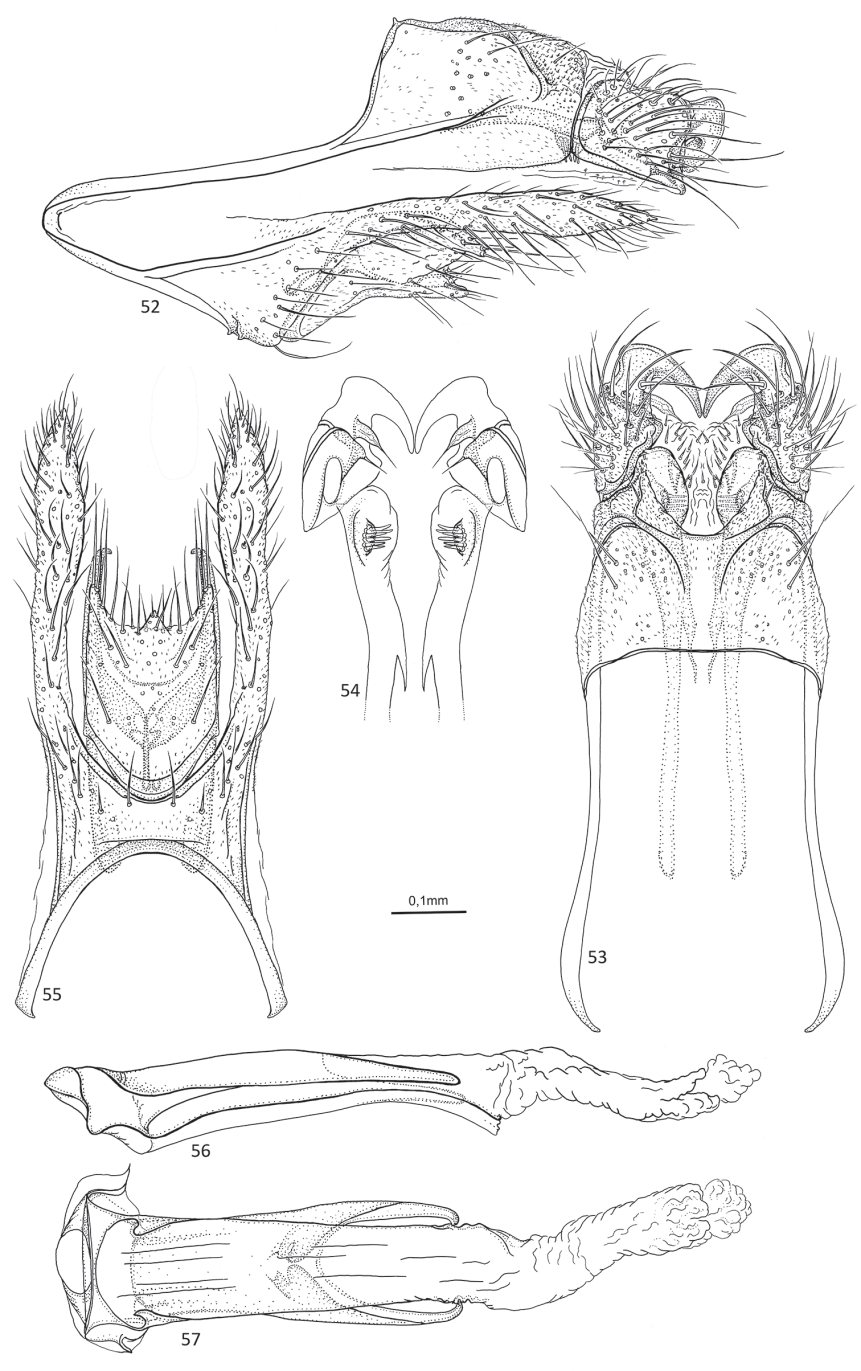
*Agmina magnahamata* sp. nov. male holotype **52** genitalia, left lateral view **53** genitalia, dorsal view **54** parameres, dorsal view **55** genitalia, ventral view **56** phallus, lateral view **57** phallus, ventral view.

##### Etymology.

*Magnahamata*, from Latin, *magna* and *hamata* (noun feminine), meaning large hook. Named for the process originating from the superior appendage, which in dorsal view is large, broad and hook-shaped.

##### Material examined.

***Holotype***: New Caledonia – **Province Sud** • ♂; western part of Mt. Ningua, Kwé Néco Stream, 3.9 km W summit of Mt. Ningua, on Boulo-Thio Road, ca. 50 m upstream road; 21°44.359'S, 166°06.009'E; 117 m; 20.xi.2003–12.xii.2003; Malaise trap; loc#035; leg. KA Johanson; MNHN.

##### Measurements.

Fore wing length 4.1 mm (*N* = 1). Total length of genitalia: 0.9 mm.

##### Description.

***Genitalia***: In lateral view, segment IX triangular anteriorly, apex located medially; in ventral view anteriorly widely U-shaped. Sternal processes, lateral view, with each apex exceeding the length of tergum X, almost reaching posterior margin of superior appendage; wide at base, then long, relatively slender, but slightly widening towards acute apex; in ventral view, almost twice as long as inferior appendage, parallel, slightly widening towards acute apex. Tergum X in lateral view largely rectangular, twice the length of superior appendage, longer than wide; in dorsal view, mesally fused, forming rounded lobe. Parameres starting before tergum X, reaching base of superior appendage, in lateral view, slender, slightly curving downwards, apex directed postero-dorsad, ventral surface with multiple wide setae directed ventrad; in dorsal view, first half parallel, long, slender, second half widening along its length, apex club shaped, on ventral surface multiple wide setae directed mesad. Superior appendages, in lateral view, largely quadratic, with large, broad hook-like process originating dorsally on posterior margin, curving downwards and back under main part, shorter than tergum X; in dorsal view stout, directed posterad, triangular, widening posteriorly, at posterior margin large hook-shaped protrusion with apex directed meso-anterad. Inferior appendages, in lateral view, with posterad orientated dorsal branch slightly longer than ventral branch, apices of both branches slightly converging, short, semi-acute, dorsal margin convex, ventral margin slightly concave; in ventral view widely bell-shaped at base, margins parallel, posterior margin concave with triangular central process. Phallus, in lateral view shorter than segment IX, slender, tubular, slightly curving downwards; in ventral view tubular, relatively wide, lateral lobes at anterior end.

##### Additional information.

This species was referred to as “sp. 42” in Espeland and Johanson (2010a).

#### Agmina longicordata sp. nov.

Taxon classificationAnimaliaTrichopteraEcnomidae

C483C132-78A0-5F5D-83C8-13699AAA7604

http://zoobank.org/0B452C33-EEBA-4BE5-9F7F-060DBF6AEE49

Figs 58–63

##### Diagnosis.

*Agmina longicordata* sp. nov. is distinguished from the other *Agmina* species by the inferior appendages in ventral view, which plate is narrowly heart-shaped and with a median process originating from the middle of its posterior margin. It is also unique by the presence of a group of small ventrally orientated setae densely arranged in a small circular area on each paramere. In addition, the sternal processes are almost boomerang-shaped, with each of the posterior part orientated in parallel. The shape of the plate formed by the inferior appendages is somewhat similar to that of *A. sagittata* sp. nov. and *A. circulata* sp. nov. but is easily separated from those species on the other characters. It resembles *A. longispina* sp. nov. in the presence of a group of densely arranged setae at the posterior part of the parameres. *Agmina longicordata* sp. nov. is easily separated from *A. longispina* sp. nov. by the oval shape of the inferior appendage plate in ventral view.

**Figures 58–63.**
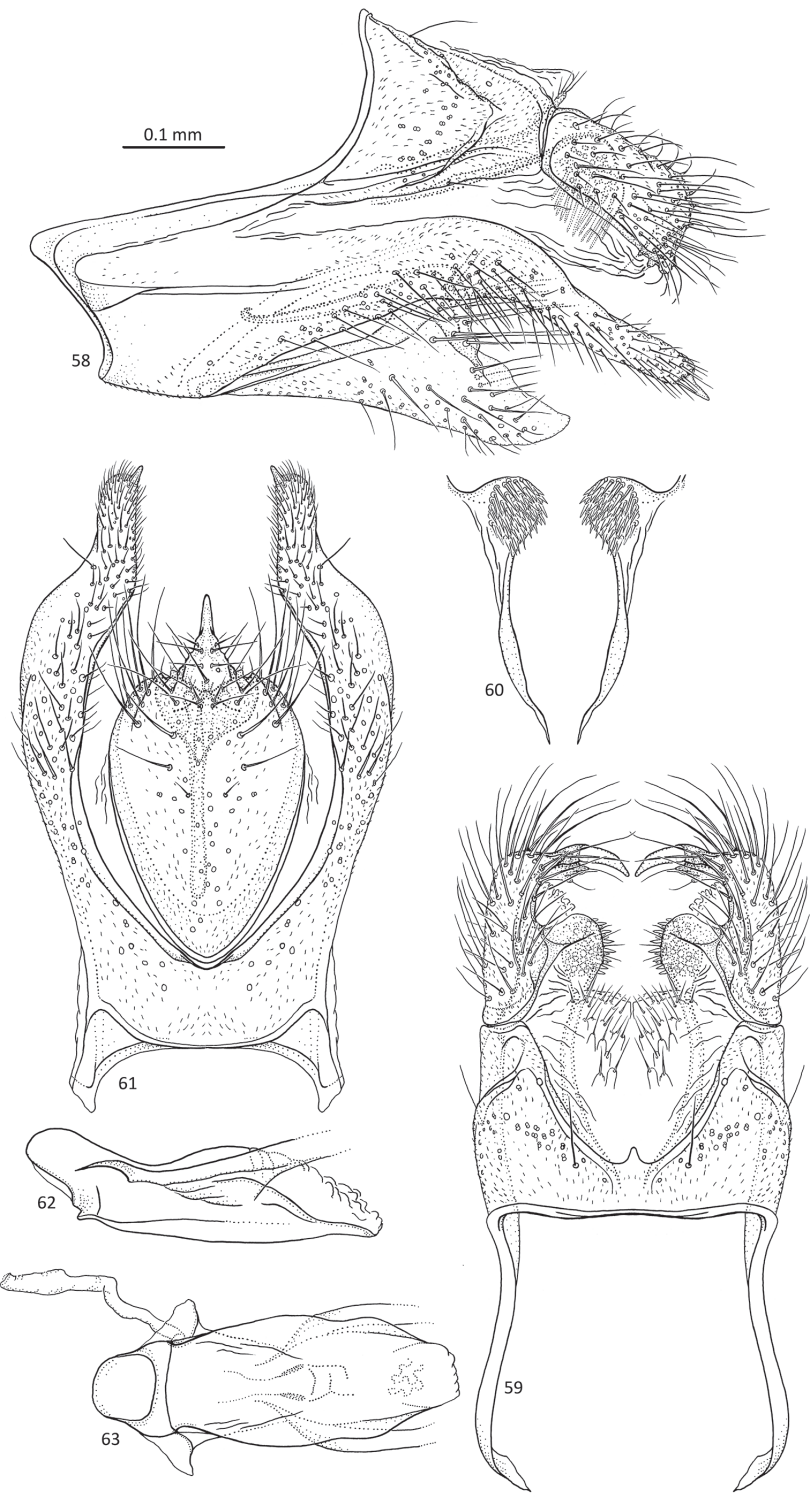
*Agmina longicordata* sp. nov. male holotype **58** genitalia, left lateral view **59** genitalia, dorsal view **60** parameres, dorsal view **61** genitalia, ventral view **62** phallus, lateral view **63** phallus, ventral view.

##### Etymology.

*Longicordata*, from Latin, *longi* and *cordata*, meaning long and heart-shaped. Named for the shape of the inferior appendage, which is shaped like an elongated heart.

##### Material examined.

***Holotype***: New Caledonia – **Province Nord** • ♂; Wan Pwé On Stream, draining NNE side of Mt. Panié, 3.9 km NW Cascade de Tao; 20°31.820'S, 164°47.016'E; 18.xii.2003; light trap; loc#085; leg. KA Johanson; MNHN.

***Paratypes***: New Caledonia – **Province Nord** • 1 ♂; same data as holotype, except NHRS; • 1 ♂; Wé Caot Stream, draining NNE side of Mt. Panié, 0.9 km NW Cascade de Tao; 20°33.311'S, 164°48.064'E; 18.xii.2003; light trap; loc#084; leg. KA Johanson; NHRS; • 1 ♂; Mt. Panié, Riv. Padyéém; 400 m; 22–28.xi.2001; Malaise trap; 22–28.xi.2001; 20°34.122'S, 164°48.147'E, loc#146 (16-2001); leg. KA Johanson, T Pape & B Viklund; NHRS.

##### Etymology.

*Cornuta*, from Latin, meaning bill of a bird. Named for the shape of the inferior appendage in lateral view, which is long, beak-like.

##### Measurements.

Fore wing length 2.4–4.0 mm (*N* = 4). Total length of genitalia: 0.7 mm.

##### Description.

***Genitalia***: In lateral view, segment IX sharply triangular anteriorly, apex located medially; in ventral view anteriorly widely oblong. Sternal processes, lateral view, with each apex exceeding the length of superior appendage, narrowing along its length, curving downwards, apex acute, spine-like; in ventral view, robust, first half slightly diverging, second half converging, wider than first half, narrower blunt apices parallel with very straight inner margin, small spine-like process at apex, directed posteromesad. Tergum X in lateral view irregularly quadrilateral with concave anterior margin, tapering towards posterior, slightly longer than superior appendage; in dorsal view, mesally fused, inner margins forming wide U-shape with small, spine-like central process. Parameres starting around the base of tergum X, in lateral view, straight, tubular, with apex widening, sharply curving ventrad, ventral surface with large amounts of wide setae directed ventroanterad; in dorsal view, robust, finger-like, outer margins convex, inner margins concave, apex rounded with large amounts of mesally directed, wide setae on inner margin. Superior appendages, in lateral view, largely rectangular, longer than wide, slightly shorter than tergum X; in dorsal view almost parallel, outer margins straight, inner margins sigmoid; bent sharply mesally at apex, ending with claw-like structure directed mesoanterad. Inferior appendages, in lateral view, with posterad orientated, rounded, lobe-like dorsal branch, ventral branch longer, tapering along its length, ventral margin straight, convex towards acute apex; in ventral view elongated cordate, posterior margin concave with large triangular central process; Phallus, in lateral view approx. half the length of segment IX, dorsal margin sigmoid, ventral margin straight; in ventral view widest at mid-length, anteriorly with lateral lobes.

##### Additional information.

This species was referred to as “sp. 29” in Espeland and Johanson (2010a).

#### Agmina campanula sp. nov.

Taxon classificationAnimaliaTrichopteraEcnomidae

2D6EAAAA-17CF-5C5F-B591-4A0B53E7854A

http://zoobank.org/77020379-094A-4E25-AC1F-5F699F791427

Figs 64–68

##### Diagnosis.

This species is very similar to *A. semicampanula* sp. nov., from which it is distinguished by the rectangular-shaped anterior margin of segment IX in ventral view; the angled sternal process in lateral view; and, in dorsal view, the pair of parameres that have sharply angled instead of rounded posteromesal corners.

**Figures 64–68.**
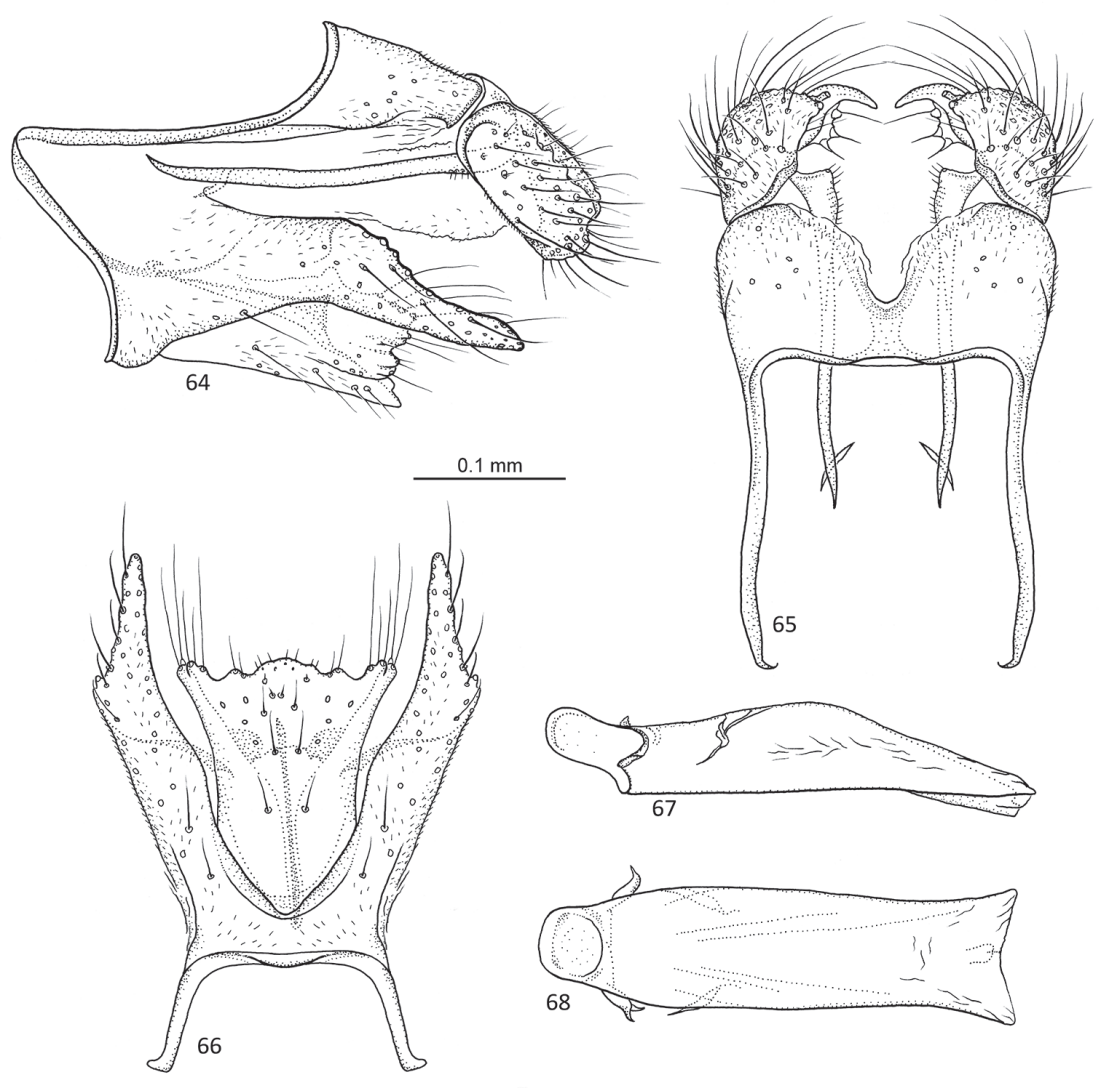
*Agmina campanula* sp. nov. male holotype **64** genitalia, left lateral view **65** genitalia, dorsal view **66** genitalia, ventral view **67** phallus, lateral view **68** phallus, ventral view.

##### Etymology.

*Campanula*, named for the bell-shaped flowers in the genus *Campanula*, based on the shape of the inferior appendages in ventral view.

##### Material examined.

***Holotype***: New Caledonia – **Province Nord** • ♂; Monts des Koghis, ca 300 m S Koghi Restaurant; 22.18288S, 166.50393E; 447 m; 15.xi.2001; Malaise trap; loc#002c (08-2001); leg. KA Johanson, T Pape & B Viklund; MNHN.

***Paratypes***: New Caledonia – **Province Sud** • 1 ♂; Rivière Ouanéoue, at bridge crossing road to Koghi Mountains, ca. 1.5 km from road RT1 Nouméa-Dumbea; 22°10.861'S, 166°29.531'E; 11.xi.2003; light trap; loc 024a; leg. KA Johanson; NHRS; • 1 ♂; Mt. Rembai, River Xwê Be, upstream bridge on road Sarraméa-Koh; 21°34.926'S, 165°49.305'E; loc#157 (027-2006); Malaise trap; 9–19.x.2006; leg. KA Johanson & M Espeland; NHRS.

##### Measurements.

Fore wing length 3.1–4.0 mm (*N* = 4). Total length of genitalia: 0.4 mm.

##### Description.

***Genitalia***: In lateral view, segment IX sharply triangular anteriorly, apex located medially; in ventral view anteriorly obround. Sternal processes, lateral view, with each apex almost reaching apex of superior appendage, narrowing along its length, curving downwards, apex acute; in ventral view, robust, diverging, first 2/3 of equal width, apices parallel, narrowing, acuminate. Tergum X in lateral view irregularly quadrilateral with concave anterior margin, tapering towards posterior, approx. same length as superior appendage; in dorsal view, mesally fused, forming rounded lobe, inner margins forming narrow bell. Parameres starting before tergum X, in lateral view long, slender, straight, acute apex slightly directed dorsad; in dorsal view, long, slender, widening at truncated apex, posterior margin straight. Superior appendages, in lateral view, oblong, with posterodorsal indentation, approx. same length as tergum X; in dorsal view stout, almost semi-circular with rounded outer margin, inner margin semi-straight, at apex large claw-shaped structure directed anteromesad. Inferior appendages, in lateral view, with posterad orientated rectangular dorsal branch and directly adjacent slender ventral branch of equal length, whole structure semi-rectangular; in ventral view bell-flower shaped. Phallus, in lateral view slightly shorter than segment IX, posterior half tapering along its length; in ventral view tubular with small lateral lobes at anterior end.

##### Additional information.

This species was referred to as “sp. 24” in Espeland and Johanson (2010a).

#### Agmina semicampanula sp. nov.

Taxon classificationAnimaliaTrichopteraEcnomidae

28726FCF-9A9E-5E00-A987-3E3363B4B02A

http://zoobank.org/D596A2CF-8922-4AAF-98E3-A94612D4BA3E

Figs 69–73

##### Diagnosis.

This species is very similar to *A. campanula* sp. nov., from which it is distinguished by the U-shaped anterior margin of segment IX in ventral view; the smoothly curved sternal process in lateral view; and, in dorsal view, the pair of parameres that have rounded instead of sharply angled posteromesal corners.

**Figures 69–73.**
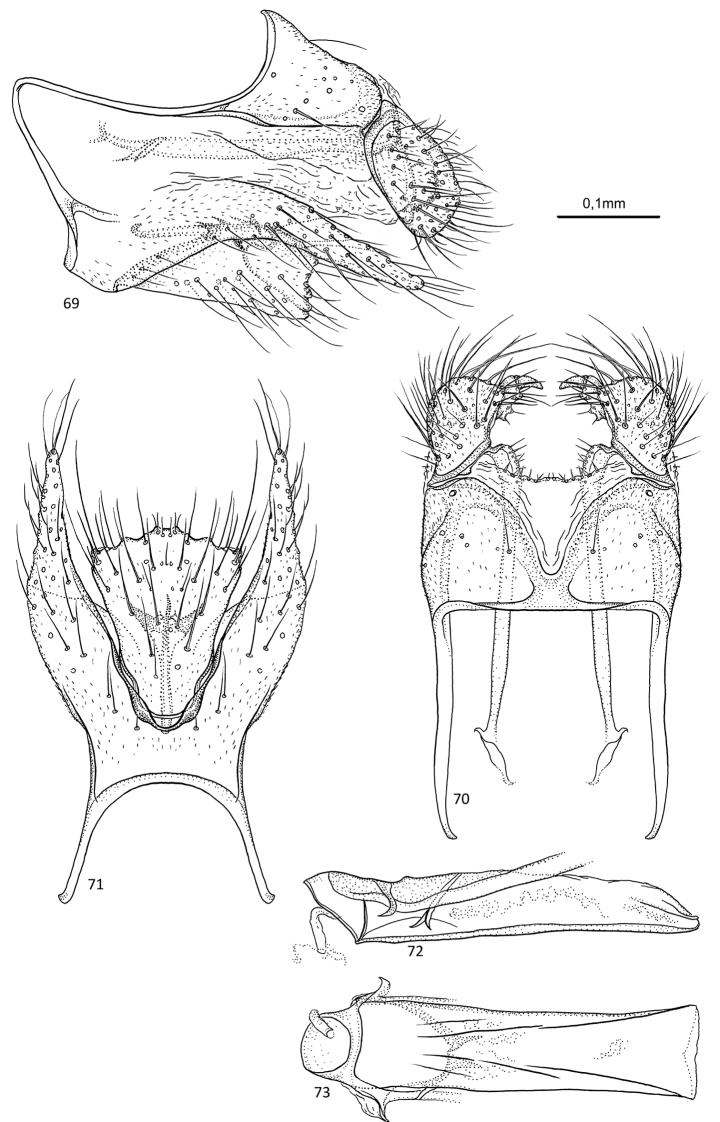
*Agmina semicampanula* sp. nov. male holotype **69** genitalia, left lateral view **70** genitalia, dorsal view **71** genitalia, ventral view **72** phallus, lateral view **73** phallus, ventral view.

##### Etymology.

Named for the similarity to the closely related species *Agmina campanula* sp. nov.

##### Material examined.

***Holotype***: New Caledonia – **Province Sud** • ♂; Mt. Dzumac, source stream of Ouinne River, near crossing point to mountain track; 22°02.073'S, 166°28.460'E; 810 m; 18.xi-4.xii.2003; Malaise trap; loc#030; leg. KA Johanson; MNHN.

##### Measurements.

Fore wing length 4.1 mm (*N* = 1). Total length of genitalia: 0.5 mm.

##### Description.

***Genitalia***: In lateral view, segment IX triangular anteriorly, apex located dorsally; in ventral view anteriorly widely U-shaped. Sternal process es, lateral view, with each apex almost reaching apex of superior appendage, narrowing along its length, slightly curving downwards, apex acute; in ventral view, robust, anterior half diverging, posterior half parallel, tapering along their length, apex acuminate. Tergum X in lateral view irregularly quadrilateral with concave anterior margin and convex posterior margin, tapering towards posterior, approx. same length as superior appendage; in dorsal view, mesally fused, forming slightly irregular lobes, inner margins forming acuminate triangle. Parameres starting before tergum X, in lateral view long, slender, straight, bifurcating at apex, with dorsal branch orientated sharply dorsad and ventral branch orientated sharply ventrad; in dorsal view, long, slender, straight, slightly converging along its length, slightly diverging and widening at blunt apex. Superior appendages, in lateral view, oblong with anterodorsal tooth, approx. same length as tergum X; in dorsal view stout, almost semi-circular with rounded outer margin, inner margin slightly concave, apex with short, stout, claw-shaped structure directed anteromesad. Inferior appendages, in lateral view, with both branches forming a single posterad orientated, rectangular structure with slightly dentated, truncated, posterior margin; in ventral view semi-bell-shaped, posterior margin slightly convex with shallow teeth. Phallus, in lateral view slightly shorter than segment IX, largely tubular, ventral margin straight; in ventral view tubular with small lateral lobes at anterior end.

##### Additional information.

This species was referred to as “FP2 *Agmina* sp. 24” in Espeland and Johanson (2010a).

#### Agmina cunicula sp. nov.

Taxon classificationAnimaliaTrichopteraEcnomidae

5B86EE80-41A0-57EC-8DC5-B93DDD5AE2A9

http://zoobank.org/9353EC29-AEB0-466F-AF10-B3FB9249C1B0

Figs 74–78

##### Diagnosis.

*Agmina cunicula* sp. nov. is distinguished from other *Agmina* species in having genitalia that are relatively simple, particularly in lateral view where they are composed of two visible branches, the upper including a large tergum X and the superior appendage, and the lower including the very broad sternal process that hide the inferior appendages and slightly exceed the superior appendages posteriorly. It resembles *A. digitata* sp. nov. but in lateral view *A. cunicula* sp. nov. have superior appendages that are rounded posteriorly, while trapezoid in *A. digitata* sp. nov. The inferior appendages of *A. cunicula* sp. nov. are very short and deeply divided longitudinally, while those in *A. digitata* form a wide and long plate that is not divided.

**Figures 74–78.**
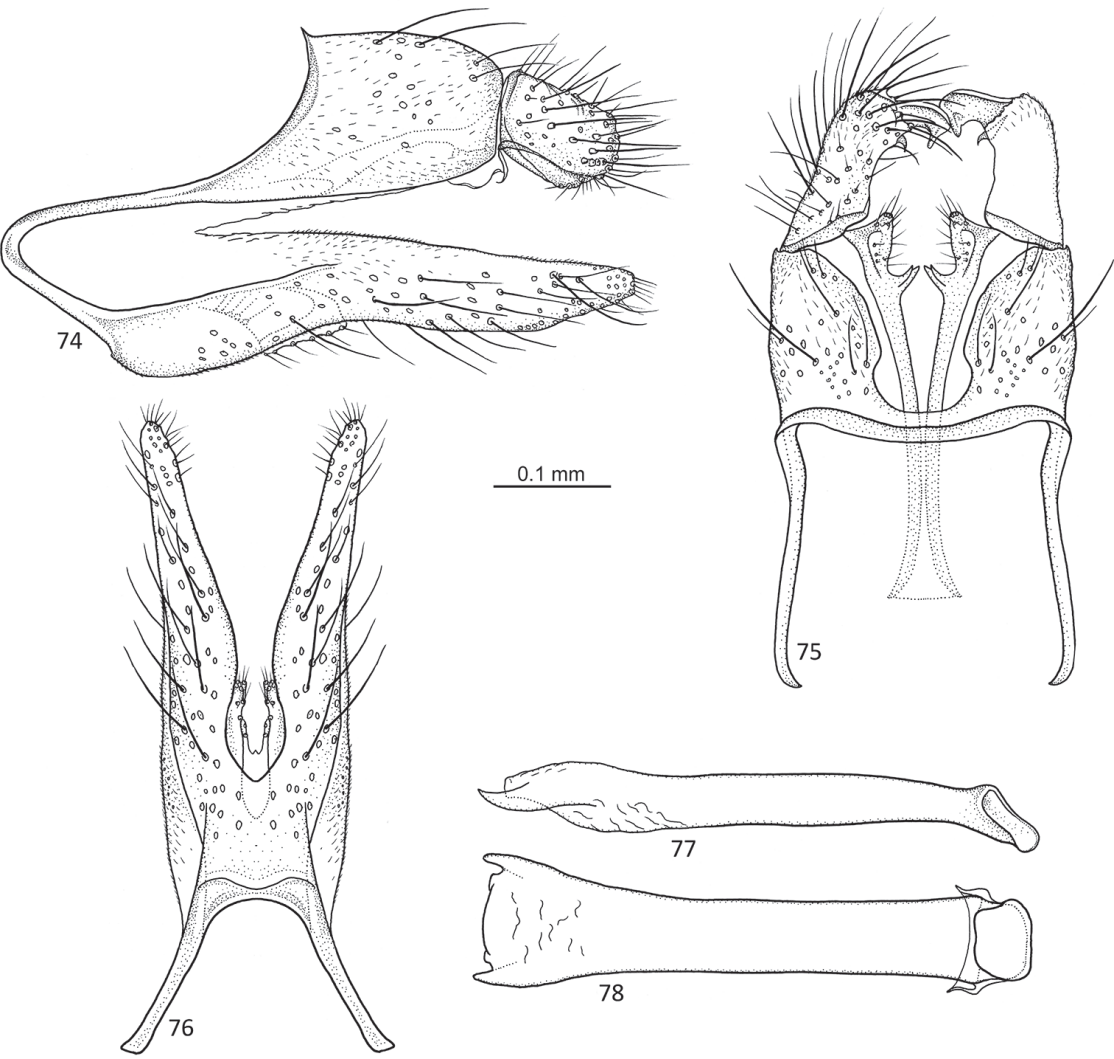
*Agmina cunicula* sp. nov. male holotype **74** genitalia, left lateral view **75** genitalia, dorsal view **76** genitalia, ventral view **77** phallus, lateral view **78** phallus, ventral view.

##### Etymology.

*Cunicula*, from Latin, meaning lengthened. Named for the very long sternal processes.

##### Material examined.

***Holotype***: New Caledonia – **Province Sud** • ♂; Plateau de Dogny; 846 m; 18–21.xi.2001; Malaise trap; 21°37.000'S, 165°52.500'E; loc#145 (15-2001); leg. KA Johanson, T Pape & B Viklund; MNHN.

***Paratype***: New Caledonia – **Province Sud** • 1 ♂; Monts des Koghis, ca 300 m S Koghi Restaurant; 22.18288S, 166.50490E; 457 m; 6.xii.2001; light trap; loc#001c (07-2001); leg. KA Johanson, T Pape & B Viklund; NHRS.

##### Measurements.

Fore wing length 4.3–4.6 mm (*N* = 2). Total length of genitalia: 0.5 mm.

##### Description.

***Genitalia***: In lateral view, segment IX narrowly U-shaped anteriorly, apex located medially; in ventral view anteriorly U-shaped. Sternal processes, lateral view, with each apex exceeding apex of superior appendage, narrowing along its length, straight, apex rounded, ventral margin slightly sigmoid, dorsal margin straight; in ventral view, long, almost parallel, slightly diverging towards rounded apex, lateral margin straight, medial margin convex. Tergum X in lateral view jar-shaped, with dorsal and ventral margins gently convex posteriorly; longer than superior appendage; in dorsal view, mesally fused, with lateral margins straight, mesal margin convex, narrowing towards posterior; inner margins forming bell-shaped structure with distended oval base. Parameres starting before tergum X, in lateral view slender, slightly curving downwards and widening at truncate apex; in dorsal view, long, slender, first half converging, second half diverging, widening towards apex, apex truncated, almost triangular with mesal process shaped like double-spine orientated posteromesad. Superior appendages, in lateral view, oblong, with slightly straighter anterior margin, shorter than tergum X; in dorsal view stout, slightly converging towards apex, outer margin almost straight, inner margin slightly concave, apex with thick hook-shaped structure and small spine directed anteromesad. Inferior appendages, in lateral view, very short, rounded structure with acute apex, at apex large, hook-shaped structure almost as long as main appendage, parallel with ventral margin of sternal process, then sharply bending, with apex pointing posterad; in ventral view with two posteriorly orientated branches, remainder hardly visible under sternal process. Phallus, in lateral view slightly shorter than segment IX, slender, tubular, with broad spine-like lateral lobe at anterior end; in ventral view tubular with anterior end slightly widening, truncate, with small spine-like lateral processes directed anterad.

##### Additional information.

This species was referred to as “sp. 11” in Espeland and Johanson (2010a).

#### Agmina cerritula sp. nov.

Taxon classificationAnimaliaTrichopteraEcnomidae

80593908-2960-5AFB-9E8F-0585BF25E0C6

http://zoobank.org/9D73F213-BE2C-4AFD-8157-D68BE6B9734E

Figs 79–83

##### Diagnosis.

Both *Agmina cerritula* sp. nov. and *A. monstrosa* sp. nov. have genitalia with inferior appendages that are fused into a single, very long, slender and dorsally curving process, which make them easily recognised from other *Agmina* species. *Agmina cerritula* sp. nov. is distinguished from *A. monstrosa* sp. nov. by having less strongly narrowing sternal processes in lateral view and that the apex is more truncated, and the superior appendages are broader posteriorly compared to those in *A. monstrosa* sp. nov. *Agmina recurvata* sp. nov. have similar inferior appendages in lateral view, but in this species the long processes are paired, not simple as in *A. serricula* sp. nov.

**Figures 79–83.**
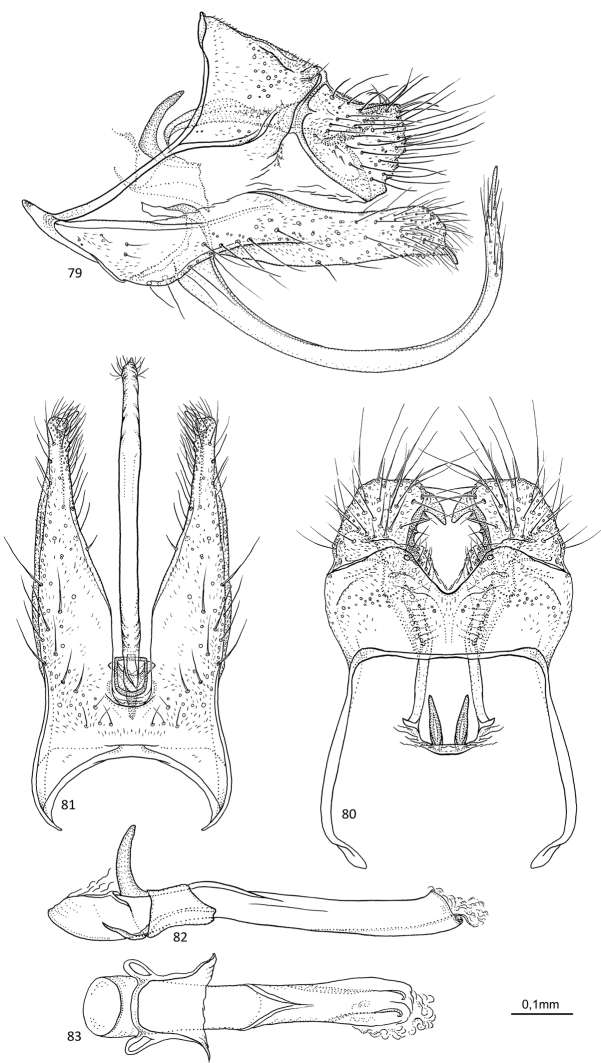
*Agmina cerritula* sp. nov. male holotype **79** genitalia, left lateral view **80** genitalia, dorsal view **81** genitalia, ventral view **82** phallus, lateral view **83** phallus, ventral view.

##### Etymology.

*Cerritula*, from Latin, meaning weird. Named for the weirdly shaped inferior appendage.

##### Material examined.

***Holotype***: New Caledonia – **Province Sud** • ♂; Creek Pernod, 7 m downstream bridge at Route du Carénage on Lac Yaté-Prony road; 22°10.862'S, 166°50.565'E; 162 m; 10.xii.2003; light trap; loc#076; leg. KA Johanson; MNHN.

##### Measurements.

Fore wing length 4.4 mm (*N* = 1). Total length of genitalia: 0.8 mm.

##### Description.

***Genitalia***: In lateral view, segment IX sharply triangular anteriorly, apex located ventrally; in ventral view anteriorly oval. Sternal processes, lateral view, with each apex exceeding apex of superior appendage, robust, largely of equal width throughout its length, margins slightly sigmoid, apex widely rounded, ventrally with short spine-like process directed posteroventrad; in ventral view, very long, tapering along their length, margins almost straight, apex blunt with short, spine-like process directed posteromesad. Tergum X in lateral view saddle-shaped, anteriorly triangular with straight dorsal margin, slightly longer than superior appendage; in dorsal view, mesally fused, almost rectangular, inner margins forming wide V-shape. Parameres starting before tergum X, in lateral view slightly widening along its length, with convex dorsal margin until mid-length, then narrowing before again widening, forming long loop at apex running parallel with ventral margin, and reaching ventral posterior corner of superior appendage; in dorsal view, separated, slender, first 1/3 slightly diverging, second 1/3 widening and diverging, third 1/3 initially parallel, then curving laterally and looping anterad. Superior appendages, in lateral view, axe-blade shaped with posterior margin slightly dentate, slightly shorter than tergum X; in dorsal view very stout, equally long as wide, almost rectangular, apex truncated, mesally with stout claw-like process directed anteromesad, followed by two small triangular processes of diminishing size. Inferior appendages, in lateral view, slender, longer than sternal process, initially directed posteroventrad, then greatly curving, forming a wide U-shape, with acuminate apex directed dorsad posterodorsally of apex of sternal process; in ventral view long, slender, tubular with straight margins, longer than sternal processes, apex acute. Phallus, in lateral view almost as long as segment IX, slender, tubular, straight, with long spine-like process near anterior end; in ventral view tubular, slightly wider at base with rounded lateral lobes and sheet-like structure.

##### Additional information.

This species was referred to as “sp. 46” in Espeland and Johanson (2010a).

#### Agmina monstrosa sp. nov.

Taxon classificationAnimaliaTrichopteraEcnomidae

96FFCAED-2712-56F9-A356-EB8D7511F792

http://zoobank.org/9D9A3F19-BD9D-4AE9-B343-0CFC9980750C

Figs 84–88

##### Diagnosis.

Both *Agmina monstrosa* sp. nov. and *A. cerritula* sp. nov. have genitalia with inferior appendages that form a very long, slender and dorsally curving process, which make them easily recognised from other *Agmina* species. *Agmina monstrosa* sp. nov. is distinguished from *A. cerritula* sp. nov. by having clearly narrowing sternal processes in lateral view and that the apex is tapering into a long, ventrally orientated tip, and the superior appendages are narrowing posteriorly instead of broadening as in *A. monstrosa* sp. nov.

**Figures 84–88.**
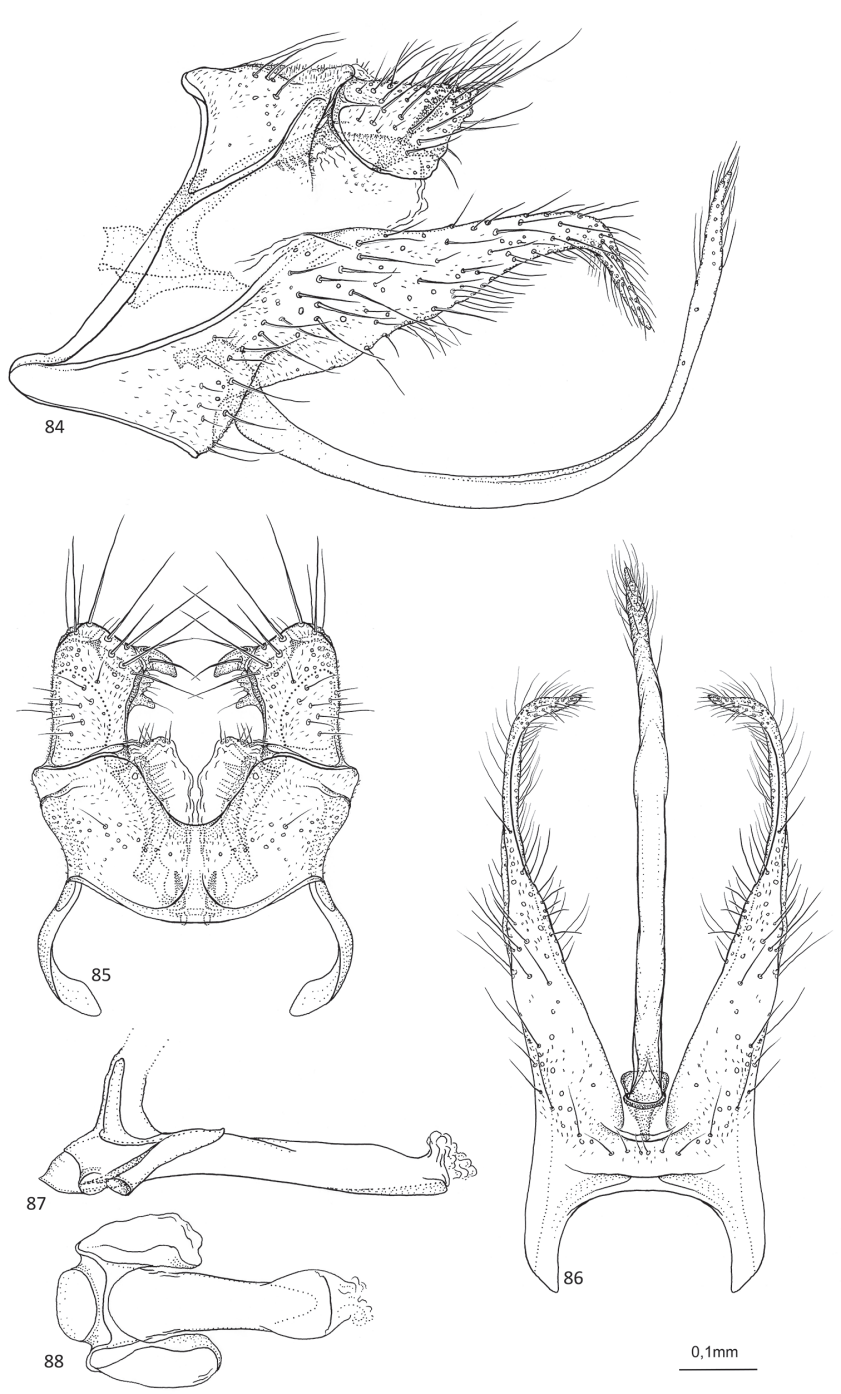
*Agmina monstrosa* sp. nov. male holotype **84** genitalia, left lateral view **85** genitalia, dorsal view **86** genitalia, ventral view **87** phallus, lateral view **88** phallus, ventral view.

##### Etymology.

*Monstrosa*, from Latin, meaning monstrous. Named for the weirdly shaped inferior appendage.

##### Material examined.

***Holotype***: New Caledonia – **Province Sud** • ♂; Dumbea River, Branche Sud; 22°08.344'S, 166°30.147'E; 42 m; 3.xi.2003; light trap; loc#006; leg. KA Johanson; MNHN.

##### Measurements.

Fore wing length 4.8 mm (*N* = 1). Total length of genitalia: 1.0 mm.

##### Description.

***Genitalia***: In lateral view, segment IX sharply triangular anteriorly, apex located ventrally; in ventral view anteriorly oblong. Sternal processes, lateral view, with each apex greatly exceeding apex of superior appendage, robust, widest at mid-length, then tapering along its length, with straight margins, acuminate apex strongly curving posteroventrad; in ventral view, very long, nearly parallel, tapering along their length, posterior third uniformly narrow, with acuminate apex directed mesad. Tergum X in lateral view saddle-shaped, anteriorly semi-triangular with almost straight dorsal margin, slightly longer than superior appendage; in dorsal view, mesally fused, almost rectangular, inner margins forming bell. Parameres starting before tergum X, in lateral view wide at base, then slender, apex complex with multiple branches, possibly forming a loop, reaching apex of superior appendage; in dorsal view, separated, initially converging, then twisting and diverging, slightly widening along their length, apex forming large loop covered by superior appendage. Superior appendages, in lateral view, small, cone-shaped with convex anteroventral margin, slightly shorter than tergum X; in dorsal view very stout, slightly longer than wide, apex rounded, mesally with stout claw-like process directed anteromesad, followed by small triangular process and slightly larger claw-like process. Inferior appendages, in lateral view, slender, longer than sternal process, initially directed posteroventrad, then widely curving, forming a very wide U-shape, with acuminate apex directed almost dorsad posterodorsally of apex of sternal process; in ventral view long, slender, tubular with almost straight margins, longer than sternal processes, apex acuminate. Phallus, in lateral view shorter than segment IX, slender, tubular, straight, with long spine-like process near anterior end; in ventral view tapering along its length, widening at apex, two large rounded lateral lobes at anterior end.

##### Additional information.

This species was referred to as “sp. 7” in Espeland and Johanson (2010a).

#### Agmina rectangulata sp. nov.

Taxon classificationAnimaliaTrichopteraEcnomidae

BE81AC5C-0F2A-556E-ADBB-2E1907A82545

http://zoobank.org/AF87DB48-A78C-4C58-BAF6-4E44DD6B5BA0

Figs 89–93

##### Diagnosis.

The inferior appendages of *A. rectangulata* sp. nov. is unique from most other species in the genus due to being almost rectangular in ventral view; and a wide, with a convex ventral margin and presence of a posterodorsal and posteroventral process when viewed from the lateral side. The inferior appendages of *Agmina diriwi* Ward & Schefter, 2000, and *A. hexacantha* Ward, 2003 resemble those of *A. rectangulata* sp. nov. in lateral view, except lacking the posterodorsal processes, and in ventral view the shape of the appendages are different, i.e., the plate is produced posteriorly in *A. rectangulata* while excised in *A. diriwi* and *A. hexacantha*. *Agmina rectangulata* is morphologically closest to *A. chela* sp. nov. *Agmina chela* sp. nov. is distinguished from *A. rectangulata* sp. nov. by the inferior appendages having slightly larger posterodorsal process in lateral view; in ventral view, a more truncate posterior margin of inferior appendages. Furthermore, the superior appendage is clearly longer than in *A. rectangulata* sp. nov. in lateral view.

**Figures 89–93.**
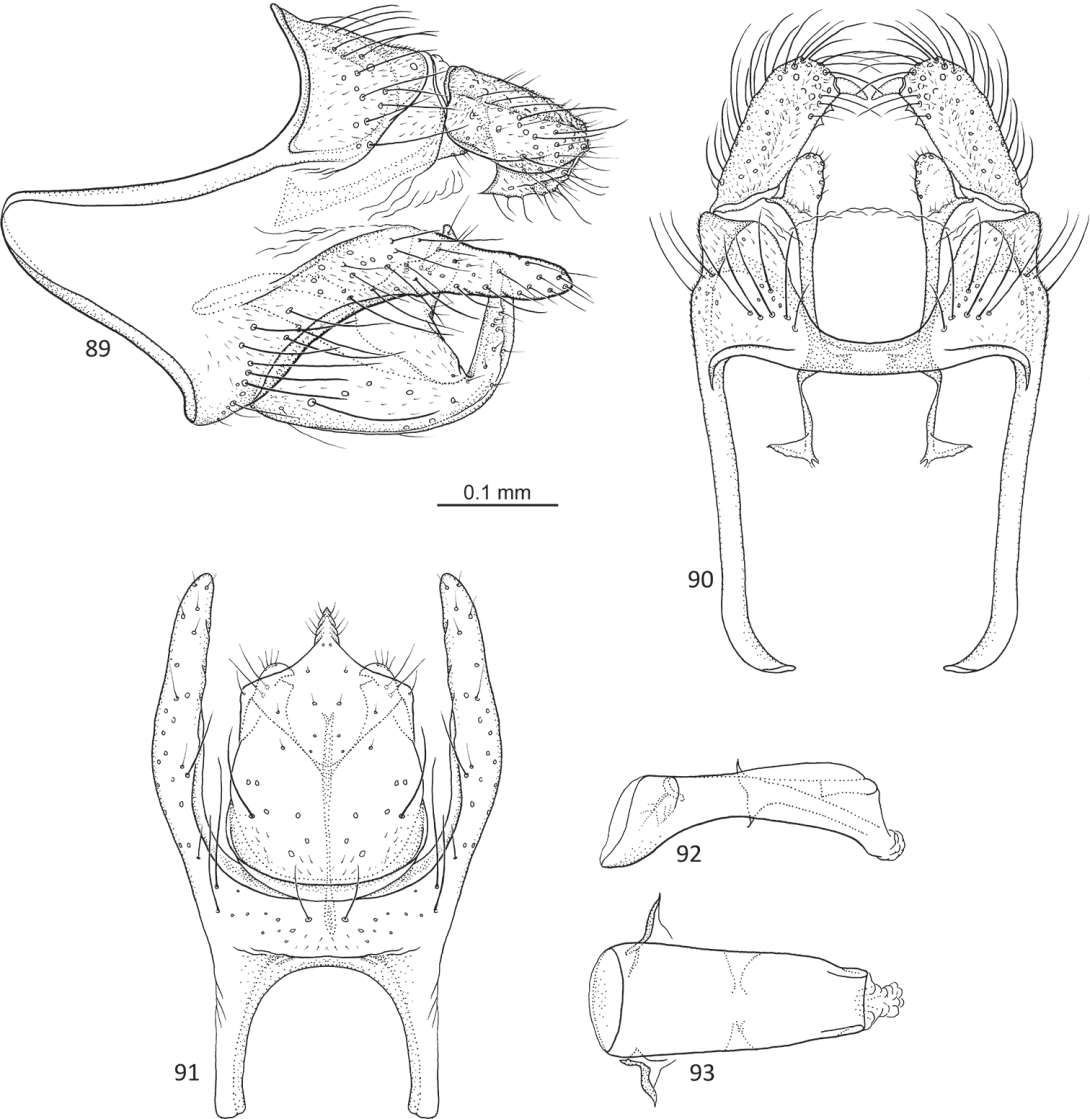
*Agmina rectangulata* sp. nov. male holotype **89** genitalia, left lateral view **90** genitalia, dorsal view **91** genitalia, ventral view **92** phallus, lateral view **93** phallus, ventral view.

##### Etymology.

*Rectangulata*, from Latin, meaning rectangular. Named for the shape of the inferior appendages having an almost rectangular impression.

##### Material examined.

***Holotype***: New Caledonia – **Province Sud** • ♂; Plateau de Dogny; 846 m; 21°37.000'S, 165°52.500'E; loc#145 (15-2001); Malaise trap; 18–21.xi.2001; leg. KA Johanson, T Pape & B Viklund; MNHN.

***Paratypes***: New Caledonia – **Province Sud** • 1 ♂; same data as holotype, except NHRS; • 1 ♂; Mt. Rembai, River Xwê Be, upstream bridge on road Sarraméa-Koh; 21°33.877'S, 165°49.922'E; loc#157; Malaise trap; 9–19.x.2006; leg. KA Johanson & M Espeland; NHRS.

##### Measurements.

Fore wing length 4.0–4.3 mm (*N* = 3). Total length of genitalia: 0.5 mm.

##### Description.

***Genitalia***: In lateral view, segment IX triangular anteriorly, apex located medially; in ventral view anteriorly U-shaped. Sternal processes, lateral view, with each apex reaching apex of superior appendage, gently curving with convex dorsal margin and concave ventral margin, apex blunt; in ventral view, parallel, ventral margin convex, mesal margin straight, apex semi-acute. Tergum X in lateral view saddle-shaped, anteriorly narrowly triangular with acuminate apex, anterior margin concave, dorsal margin straight, approx. same length as superior appendage; in dorsal view, mesally fused, irregular quadrilateral, inner margin widely U-shaped. Parameres starting at base of tergum X, in lateral view wider at base, then slender, tubular, second half extremely wide sheet-like, curving laterally and ventrally, with apex below superior appendage; in dorsal view, separated, wider at base, then slender, tubular, wider, club-shaped apex with lateral spine. Superior appendages, in lateral view, nearly oblong with straighter anterior margin, similar in length to tergum X; in dorsal view converging, longer than wide, finger-shaped with rounded apex with claw-like spine directed anteromesad, two small triangular processes on mesal margin anteriorly of claw. Inferior appendages, in lateral view, with dorsoposterad orientated shorter, claw-shaped dorsal branch; ventral branch longer, strongly curving at base, then straight, orientated dorsad; in ventral view almost rectangular with oval anterior margin, posterior margin widely triangular with ventral process forming acuminate apex, dorsal processes barely exceeding posterior margin, apices rounded with triangular notch mesally, lateral margins slightly undulating. Phallus, in lateral view shorter than segment IX, dorsal margin straight, ventral margin concave; in ventral view tubular, slightly tapering towards apex, with triangular lateral lobes near anterior end.

##### Additional information.

This species was referred to as “sp. 45” in Espeland and Johanson (2010a).

#### Agmina chela sp. nov.

Taxon classificationAnimaliaTrichopteraEcnomidae

452D80E4-7C92-5FC3-9206-674D81ABB53E

http://zoobank.org/7547FEDE-D338-4EB3-8B5D-DE125EBE80CB

Figs 94–98

##### Diagnosis.

The inferior appendages of *A. chela* sp. nov. are distinguished from those of most other species in the genus due to being almost rectangular in ventral view; and a wide, with a convex ventral margin and presence of a posterodorsal and posteroventral process when viewed from the lateral side. The inferior appendages of *Agmina diriwi* Ward & Schefter, 2000, and *A. hexacantha* Ward, 2003 resemble those of *A. chela* sp. nov. in lateral view, except lacking the posterodorsal processes, and in ventral view the shape of the appendages is different, i.e., the plate is produced posteriorly in *A. chela* while excised in *A. diriwi* and *A. hexacantha*. The species that is closest morphologically is *A. rectangulata* sp. nov. *Agmina chela* sp. nov. is distinguished from *A. rectangulata* sp. nov. in the inferior appendages by the, in lateral view, slightly larger posterodorsal process; in ventral view, a more truncate posterior margin of inferior appendages; and the superior appendage is clearly longer than in *A. rectangulata* sp. nov., as seen in lateral view.

**Figures 94–98.**
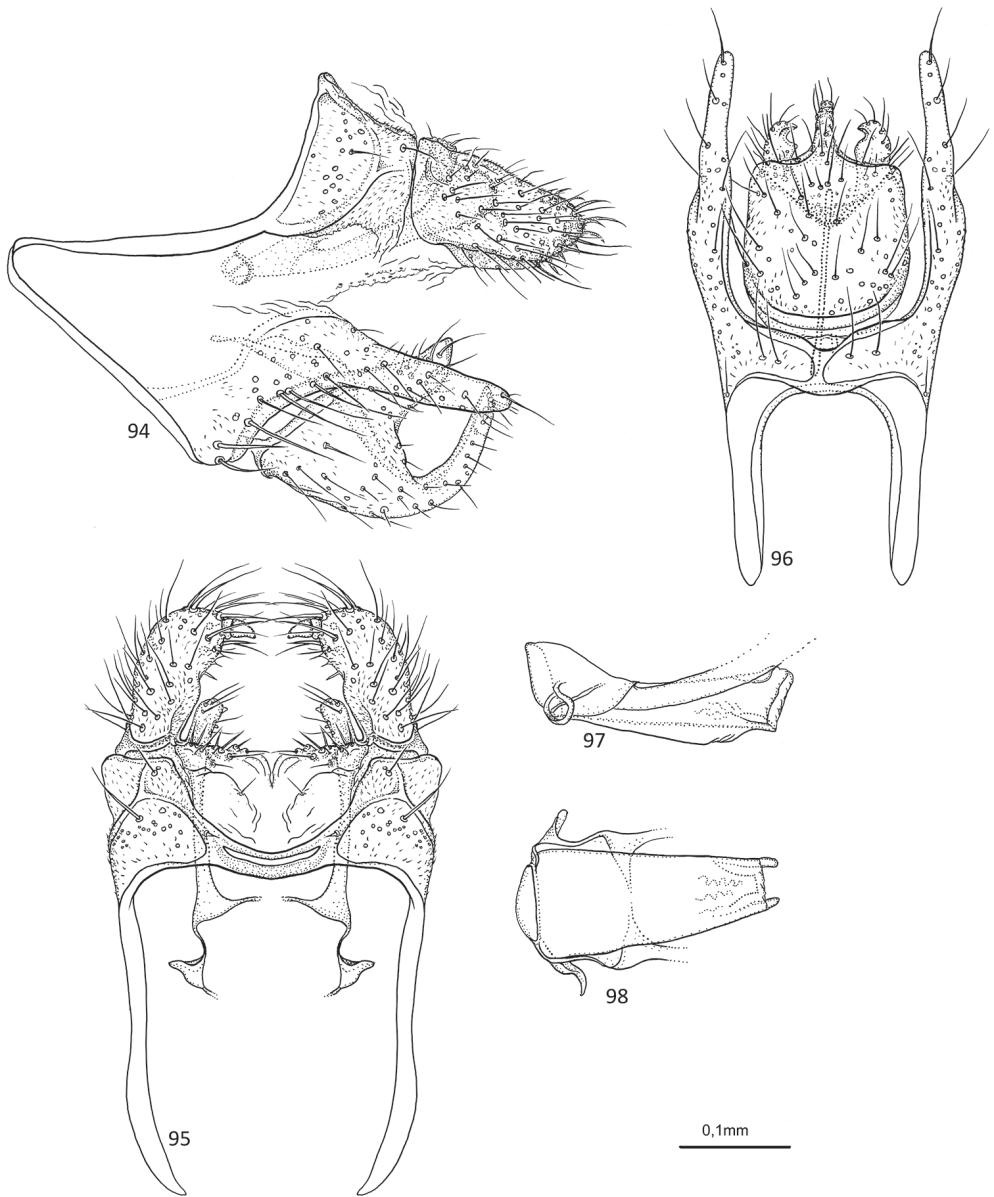
*Agmina chela* sp. nov. male holotype **94** genitalia, left lateral view **95** genitalia, dorsal view **96** genitalia, ventral view **97** phallus, lateral view **98** phallus, ventral view.

##### Etymology.

From Greek *chela* (noun, feminine), meaning pincer-like claw. Named for the crab-claw shaped inferior appendage in lateral view.

##### Material examined.

***Holotype***: New Caledonia, Province Sud, Monts des Koghis, ca 300 m S Koghi Restaurant, 22.18288S, 166.50167E, 457 m, 16–26.xi.2003, Malaise trap, loc#001a [KA Johanson].

***Paratypes***: New Caledonia – **Province Sud** • 1 ♂; Monts des Koghis, ca 300 m S Koghi Restaurant; 22.18288S, 166.50245E; 427 m; 2–16.xi.2003, Malaise trap, loc#003; leg. KA Johanson; NHRS; • 1 ♂; Monts des Koghis, ca 300 m S Koghi Restaurant; 22.18288S, 166.50167E; 417 m; 2–16.xi.2003; Malaise trap; loc#004; leg. KA Johanson; NHRS.

##### Measurements.

Fore wing length 4.0–4.3 mm (*N* = 3). Total length of genitalia: 0.5 mm.

##### Description.

***Genitalia***: In lateral view, segment IX triangular anteriorly, apex located medially; in ventral view anteriorly U-shaped. Sternal processes, lateral view, with each apex almost reaching posterior apex of superior appendage, gently curving with convex dorsal margin and concave ventral margin, apex blunt; in ventral view, long, slender, almost parallel, blunt apex. Tergum X in lateral view anteriorly narrowly triangular with acuminate apex, anterior margin straight, dorsal margin slightly concave, shorter than superior appendage; in dorsal view, mesally fused, semi-rectangular, inner margins forming very wide U-shape. Parameres starting before tergum X, in lateral view widening along its length, apex club-shaped, not exceeding superior appendage; in dorsal view, separated, straight, club-shaped apices slightly converging, with complex, sheet-like structure curving ventrally, ending just below superior appendage, anterior end semi-triangular. Superior appendages, in lateral view, longer than wide, anterior margin straight, apex rounded, slightly tapering along its length; in dorsal view robust, longer than wide, apex rounded with claw-shaped spine directed mesoanterad. Inferior appendages, in lateral view, with dorsoposterad orientated slightly shorter, but broader straight dorsal branch; ventral branch longer, curving at base, then straight, orientated dorsoposterad; in ventral view almost rectangular with oval anterior margin, posterior margin almost straight, narrow ventral process with blunt apex, dorsal processes clearly exceeding posterior margin, apices rounded with rounded notch mesally, lateral margins straight. Phallus, in lateral view shorter than segment IX, dorsal margin concave, ventral margin almost straight; in ventral view uniformly tapering along its length, with narrow lateral lobes at anterior end.

##### Additional information.

This species was referred to as “sp. 55” in Espeland and Johanson (2010a).

#### Agmina piscaria sp. nov.

Taxon classificationAnimaliaTrichopteraEcnomidae

545728A4-E830-5380-99E1-FA8413826906

http://zoobank.org/AEBF5739-478F-4FD1-B86D-E72A2A0EB6D3

Figs 99–104

##### Diagnosis.

*Agmina piscaria* sp. nov. is distinguished from other Agmina species in combination of having a subapical instead of apical position of the median hooks of the superior appendage, dorsal branch of inferior appendages that are hooked mesally at their apex, seen in dorsal view, the shape of the plate formed by the inferior appendages that is narrowly parallelogram-shaped with a long central lobe, and long, straight sternal process. Also *A. amieuensis* sp. nov. and *A. christinae* sp. nov. have a subapical position of the mesal hooks of the superior appendages, but *A. amieuensis* sp. nov. is separated from *A. piscaria* sp. nov. by the tapering superior appendage in lateral view, the short sternal processes, and the different shape of the inferior appendage. *Agmina christinae* sp. nov. has a group of megasetae on the dorsobasal part of the superior appendages that are absent in *A. piscaria* sp. nov.

**Figures 99–104.**
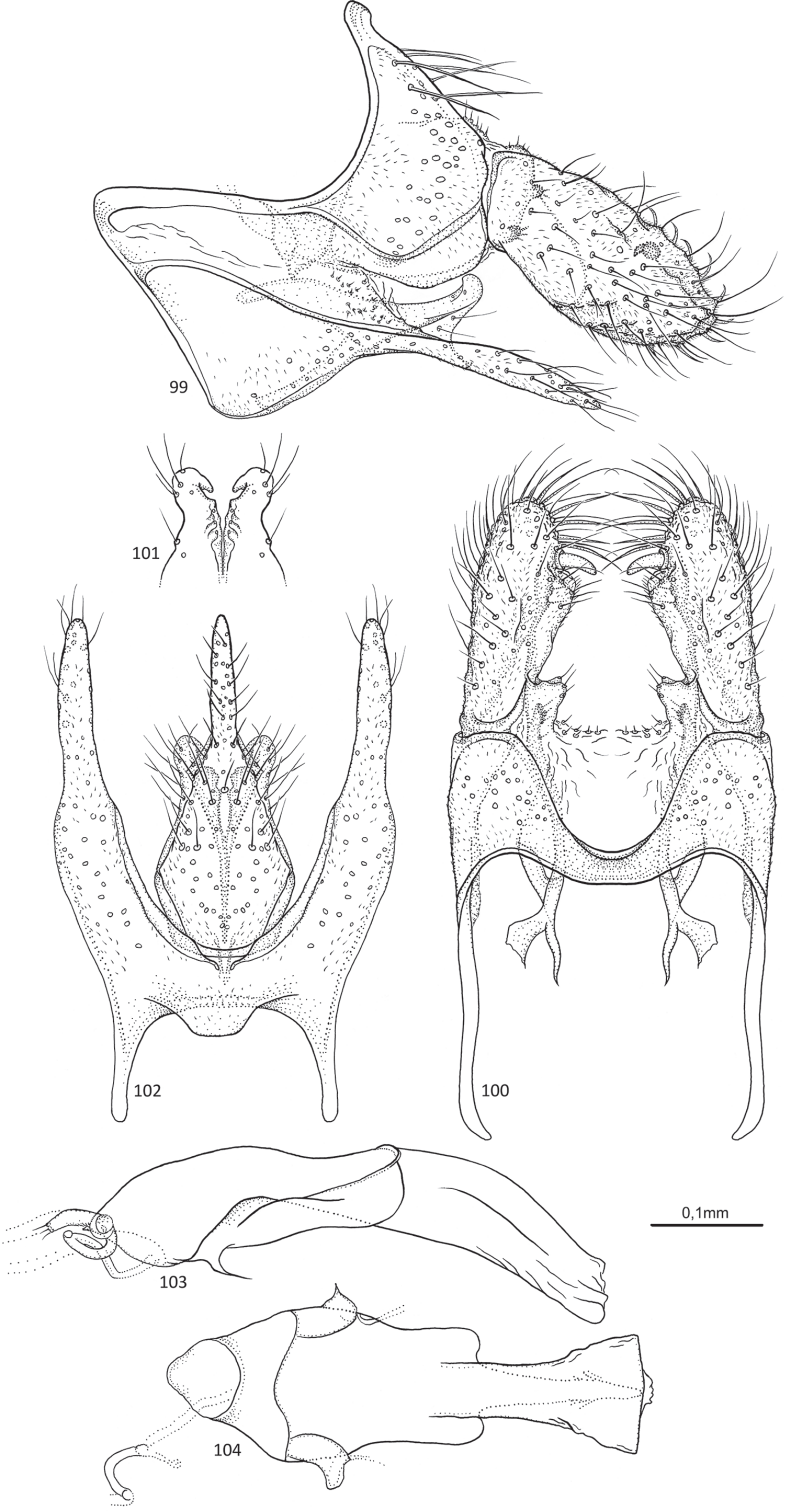
*Agmina piscaria* sp. nov. male holotype **99** genitalia, left lateral view **100** genitalia, dorsal view **101** posterodorsal part of inferior appendages, dorsal view **102** genitalia, ventral view **103** phallus, lateral view **104** phallus, ventral view.

##### Etymology.

*Piscaria*, from Latin, meaning fish-like. Named for the fish-shaped phallus in ventral view.

##### Material examined.

***Holotype***: New Caledonia – **Province Sud** • ♂; Mt. Panié; 20.57306S, 164.77139E; 902 m; 9.xii.2003; Malaise trap; loc#075; leg. KA Johanson; MNHN.

##### Measurements.

Fore wing length 4.5 mm (*N* = 1). Total length of genitalia: 0.6 mm.

##### Description.

***Genitalia***: In lateral view, segment IX triangular anteriorly, apex located medially; in ventral view anteriorly oblong. Sternal processes, lateral view, with each apex almost reaching posterior apex of superior appendage, anterior half triangular, abruptly narrowing around mid-length, posterior half slender, straight with semi-acute apex, directed posteroventrad; in ventral view, anterior half robust, slightly diverging, posterior half narrower, almost parallel, apices blunt. Tergum X in lateral view anterodorsally narrowly triangular, with anterior margin concave, dorsal margin straight, ventral margin convex, shorter than superior appendage; in dorsal view, mesally fused, strongly concave anteriorly, inner margin forming U-shape. Parameres in lateral view anteriorly triangular, then abruptly narrowing, midpart very slender, curving, apex much wider, club-shaped, directed dorsad, with two warts at posterior margin; in dorsal view, widely separated, bifurcated anteriorly, slender, anterior half slightly diverging, posterior half parallel, narrowly, club-shaped at apex. Superior appendages, in lateral view, largely oval, longer than wide, with wide setae at posterior part of dorsal margin; in dorsal view robust, slightly converging, lateral margin straight, mesal margin convex with claw-like process directed anteromesad near rounded. Inferior appendages, in lateral view, with dorsoposterad orientated dorsal branch with rounded apex slightly longer than ventroposterad-directed narrow, acuminate, ventral branch; in ventral view rhomboid, with ventral branch forming long, acute, central process, club-shaped dorsal processes exceeding posterior margin. Phallus, in lateral view as long as segment IX, tubular, of equal width along its length, curving upwards towards posterior; in ventral view fish-shaped.

##### Additional information.

This species was referred to as “sp. 30” in Espeland and Johanson (2010a).

### Species group 3, *kapiwa* group

Included species in this group are: *Agmina kapiwa* Ward & Schefter, 2000, *A. amplexa* sp. nov. and *A. caraffa*, sp. nov.

#### Agmina amplexa sp. nov.

Taxon classificationAnimaliaTrichopteraEcnomidae

902CC180-6ED1-5A1A-87F1-C7C5EB88D3AD

http://zoobank.org/D8630A6F-9F11-453F-AFE0-CFBCEDB8E5EB

Figs 105–109

##### Diagnosis.

*Agmina amplexa* sp. nov. is unique in the genus in that the superior appendages are strongly modified with three strong hooks that are orientated mesad as seen in dorsal view. Also *A. complexa* sp. nov. have strongly modified superior appendages with large hooks but in *A. complexa* sp. nov. there are only one pair, which are strongly curved anteriorly instead of directed mesally, as seen in dorsal view.

**Figures 105–109.**
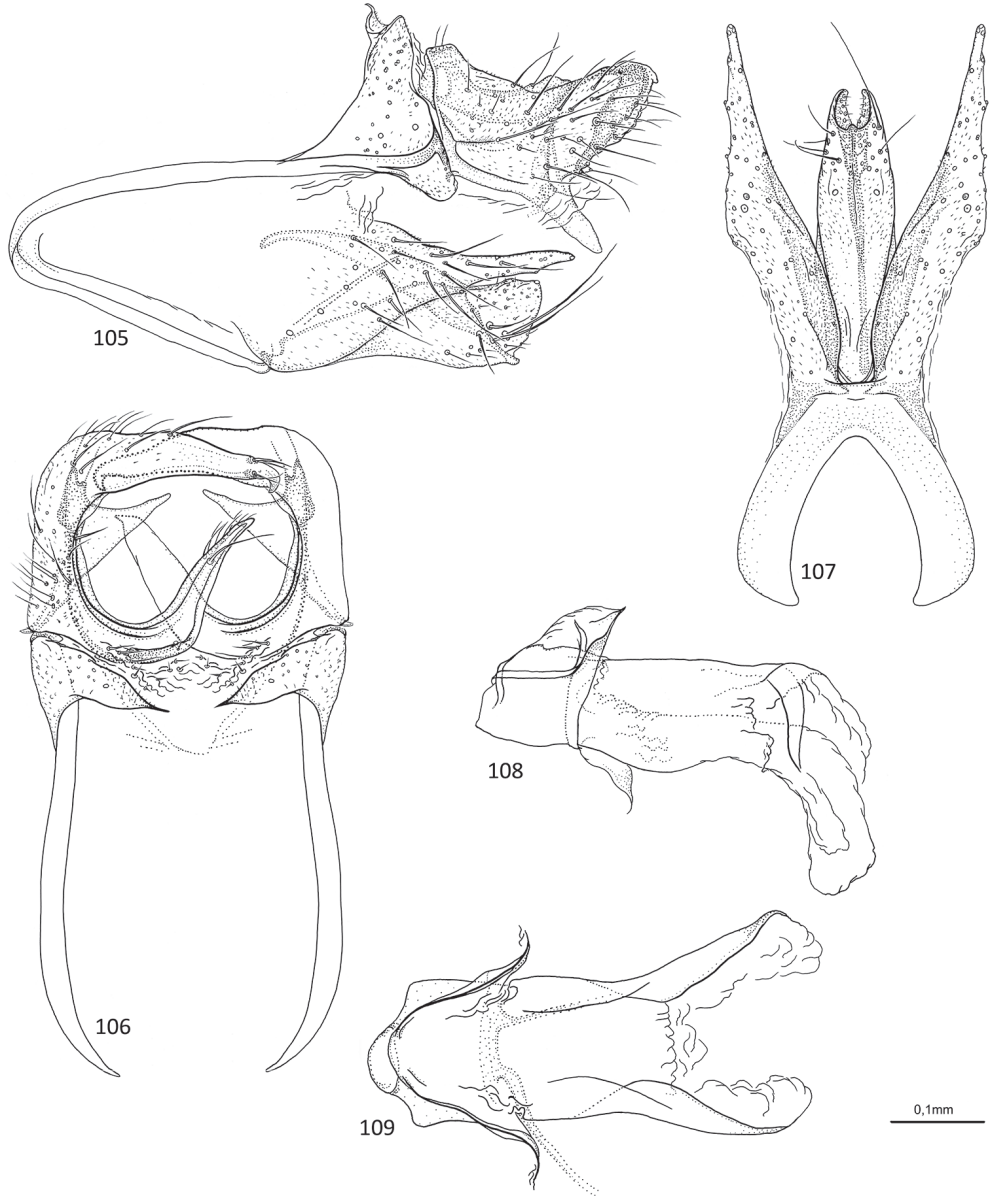
*Agmina amplexa* sp. nov. male holotype **105** genitalia, left lateral view **106** genitalia, dorsal view **107** genitalia, ventral view **108** phallus, lateral view **109** phallus, ventral view.

##### Etymology.

*Amplexa*, from Latin, meaning embrace. Named for the superior appendages in dorsal view encircling each other.

##### Material examined.

***Holotype***: New Caledonia – **Province Sud** • ♂; Mt. Dzumac, source stream of Ouinne River, downstream crossing point to mountain track; 22°01.997'S, 166°28.486'E; 795 m; over ca. 30 m waterfall; 18.xi-4.xii.2003; Malaise trap; loc#031; leg. KA Johanson; MNHN.

***Paratype***: New Caledonia – **Province Sud** • 1 ♂; Mt. Dzumac, source stream of Ouinne River, at crossing point to mountain track; 22°02.218'S, 166°28.566'E; 797 m; 18.xi.2003; light trap; loc#032; leg. KA Johanson; NHRS.

##### Measurements.

Fore wing length 3.5–4.2 mm (*N* = 2). Total length of genitalia: 0.7 mm.

##### Description.

***Genitalia***: In lateral view, segment IX narrowly U-shaped anteriorly, apex located mesally; in ventral view anteriorly ovoid. Sternal processes, lateral view, with each apex reaching posterior half of superior appendage, straight, directed posterad, narrowing along its length, apex acuminate. in ventral view, diverging, lateral margins convex, mesal margins slightly convex anteriorly, apices acuminate. Tergum X in lateral view triangular; in dorsal view, narrowly triangular with concave posterior margin. Parameres absent. Superior appendages, in lateral view, twice as long as tergum X, saddle-shaped with spine like ventral process, two tubular, curving mesal processes, the anterior largely parallel with dorsal margin, apex blunt, the posterior parallel with posterior margin with acute apex; in dorsal view long, anterior half straight, parallel, curved mesad at 90 degree angle at mid-length, tapering along its length, apex claw-like directed anterad; main branch bifurcating anteriorly, with slender mesal branch curving upwards with acuminate apex; second, narrowly triangular mesal branch originating between first mesal branch and the curve of main branch. Inferior appendages, in lateral view, with posterad orientated large, lobe-like, acute dorsal branch, ventral branch in the form of small tooth; in ventral view dorsal branch forming long, slender shape with convex margins and two slender, slightly converging posterior processes, ventral branch forming small central tooth. Phallus, in lateral view shorter than segment IX, tubular, straight, with anterior lateral lobes; in ventral view slightly wider posteriorly, thin lateral lobes anteriorly.

##### Additional information.

This species was referred to as “sp. 8” in Espeland and Johanson (2010a).

#### Agmina caraffa sp. nov.

Taxon classificationAnimaliaTrichopteraEcnomidae

E4BA0087-7E3D-5A88-B65F-EEF1B863A85D

http://zoobank.org/877F45E8-18AC-430F-880F-7F05FB9BA902

Figs 110–114

##### Diagnosis.

*Agmina caraffa* sp. nov. is distinguished from all other *Agmina* species in the presence of a pair of long, slender and curving parameres, each armed with tubular megasetae at the meso-ventral face of the posterodorsal apex; and a perfect triangular superior appendage in lateral view.

**Figures 110–114.**
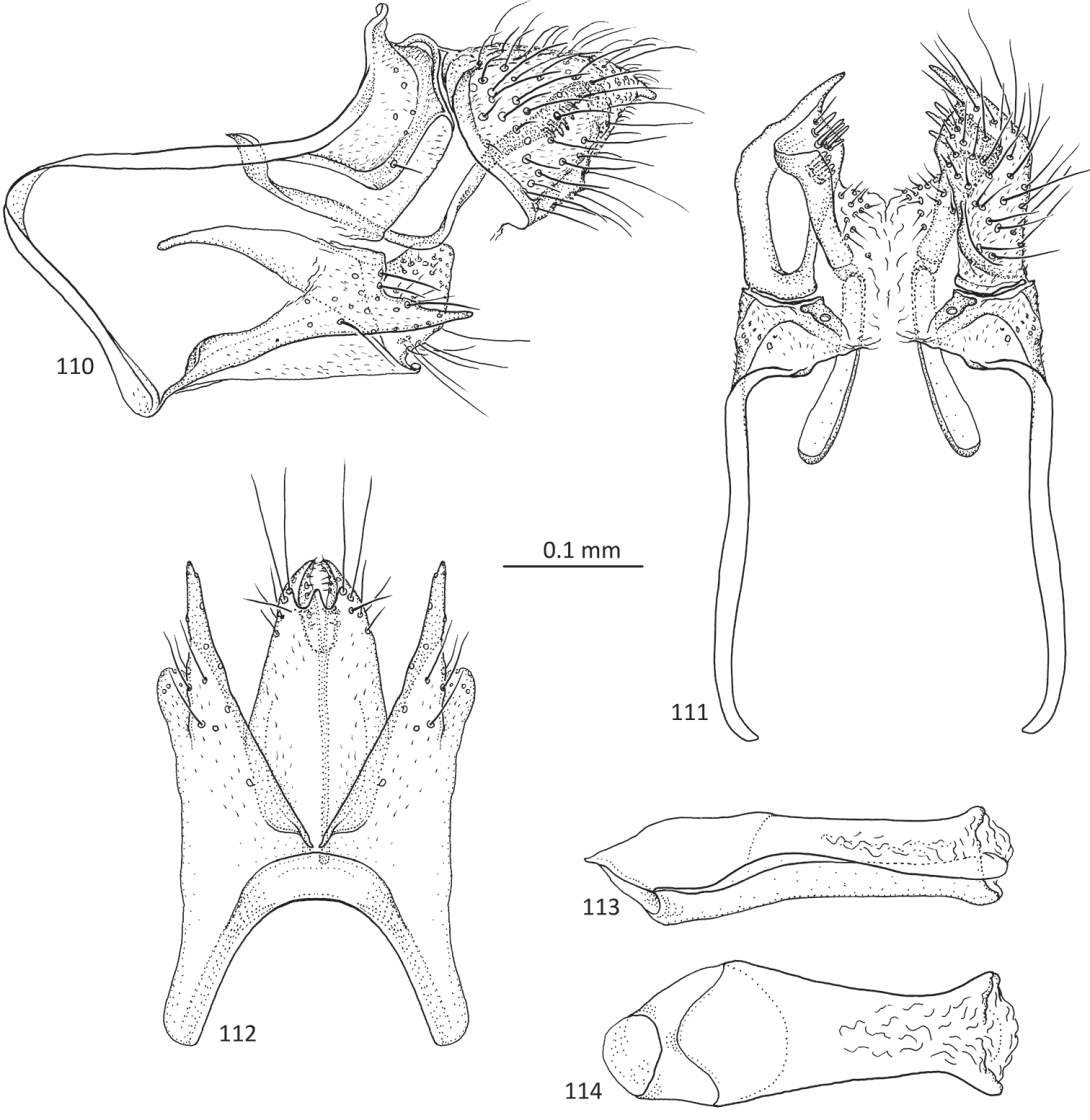
*Agmina caraffa* sp. nov. male holotype **110** genitalia, left lateral view **111** genitalia, dorsal view **112** genitalia, ventral view **113** phallus, lateral view **114** phallus, ventral view.

##### Etymology.

Named for the carafe-shaped phallus in lateral view.

##### Material examined.

***Holotype***: New Caledonia – **Province Sud** • ♂; stream crossing Nouméa-Yaté road immediately W of turnoff to Rivière Bleue Reserve; 22°10.191'S, 166°44.474'E; 162 m; 22.xi-4.xii.2003; Malaise trap; loc#040; leg. KA Johanson; MNHN.

##### Measurements.

Fore wing length 3.2 mm (*N* = 1). Total length of genitalia: 0.6 mm.

##### Description.

***Genitalia***: In lateral view, segment IX semi-triangular with rounded apex located medially; in ventral view anteriorly widely U-shaped. Sternal processes, lateral view, with each apex barely exceeding posterior apex of tergum X, ventral margin almost straight, dorsal margin with rectangular protrusion after mid-length, apex acuminate; in ventral view, parallel, triangular with acuminate apices, rounded lobes after mid-length on lateral margin. Tergum X in lateral view pot-shaped with anterior margin concave, posterior margin straight, higher than long; in dorsal view, irregularly quadrilateral with slightly concave posterior margin. Parameres long, in lateral view slender equally wide along their length, curving upwards towards narrowly club-shaped apex with megasetae at ventral margin, ending before apex of superior appendage; in dorsal view, anterior half slender, converging, posterior half diverging, apex complex sheet-like folding, with megasetae on mesal surface. Superior appendages, in lateral view, triangular, twice as long as tergum X, apex with short spine; in dorsal view stout, longer than wide, slightly widening along their length, abruptly narrowing towards posteromesad directed spine-like apex. Inferior appendages, in lateral view, with posterad orientated large, lobe-like, blunt dorsal branch, ventral branch in the form of small tooth; in ventral view dorsal branch forming slender shape narrowing along its length, with slightly convex margins, two slender, converging posterior processes, ventral branch forming central tooth. Phallus, in lateral view slightly shorter than segment IX, carafe-shaped, straight; in ventral view tubular, straight.

##### Additional information.

This species was referred to as “sp. 15” in Espeland and Johanson (2010a).

### Species group 4, *nodosa* group

Included species in this group are: *Agmina nodosa* Ward, 2003, *A. rostrata* sp. nov., *A. artarima* Ward & Schefter, 2000, *A. dathioensis* sp. nov., *A. rougensis* sp. nov., *A. viklundi* sp. nov., *A. lata* sp. nov., *A. falx* sp. nov., *A. guttula* sp. nov., *A. amieuensis* sp. nov., *A. spina* sp. nov., *A. complexa* sp. nov., *A. berada* Ward & Schefter, 2000, and *A. cheirella* Ward, 2003.

The species in the *nodosa* species group are characterised by having relatively short inferior appendage that is simple and curving dorsally in lateral view.

#### Agmina rostrata sp. nov.

Taxon classificationAnimaliaTrichopteraEcnomidae

47A5D1ED-CBDA-540E-82F3-59EE86D5AADD

http://zoobank.org/B9FD1DF2-4FF7-45D3-B794-931C0DD18D5D

Figs 115–119

##### Diagnosis.

*Agmina rostrata* sp. nov. is characterised by having long posteriorly orientated sternal processes that are almost parallel-sided along their length, and heavily sclerotised parameres with posterior part orientated dorsally and deeply bifurcated in lateral view. Also *A. dathioensis* sp. nov., *A. rougensis* sp. nov. and *A. lata* sp. nov. have long and parallel-sided sternal processes in lateral view. *Agmina rostrata* sp. nov. is distinguished from *A. dathioensis* sp. nov. in that the sternal process is orientated posteriorly, not ventrally; and from *A. lata* sp. nov. in the slightly narrower sternal process in lateral view, and absence of megasetae on the posterior apex of the parameres on the mesal face of the superior appendages.

**Figures 115–119.**
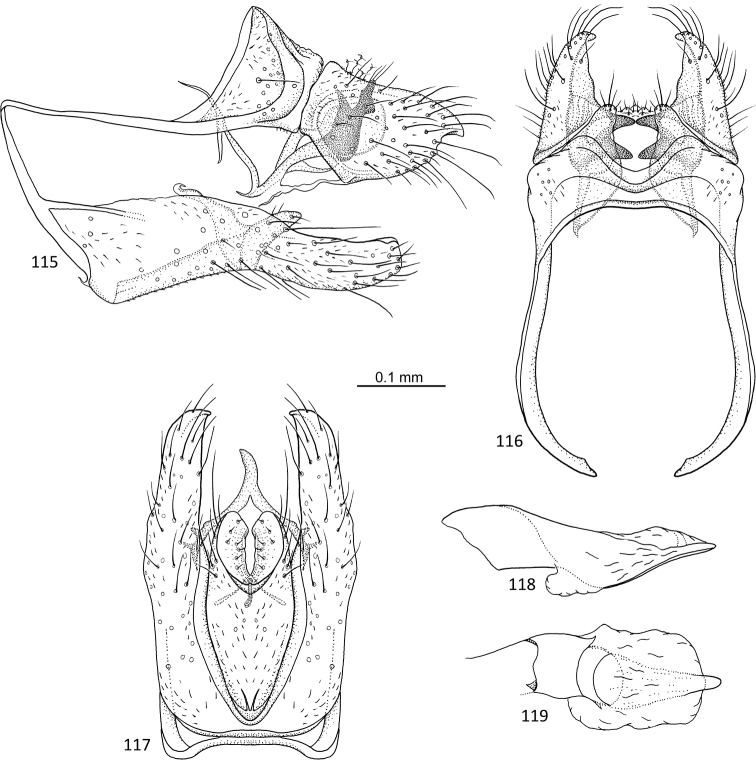
*Agmina rostrata* sp. nov. male holotype **115** genitalia, left lateral view **116** genitalia, dorsal view **117** genitalia, ventral view **118** phallus, lateral view **119** phallus, ventral view.

##### Etymology.

*Rostrata*, from Latin, meaning beak-shaped. Named for the medial processes on the superior appendage in ventral view, together forming a beak-shaped process.

##### Material examined.

***Holotype***: New Caledonia – **Province Sud** • ♂; Dumbea River, Branche Nord, 2.2 km SE summit of Mt. Piditéré; 22°07.503'S, 166°29.899'E; 25 m; 21.i.2004; light trap; loc#124a; leg. KA Johanson & C Pöllabauer; MNHN.

***Paratype***: New Caledonia – **Province Sud** • 1 ♂; same data as holotype; • 1 ♂; Dumbea River, Branche Sud; 22°08.344'S, 166°30.147'E; 42 m; 3.xi.2003; light trap; loc#006; leg. KA Johanson; NHRS.

##### Measurements.

Fore wing length 2.9–3.5 mm (*N* = 2). Total length of genitalia: 0.5 mm.

##### Description.

***Genitalia***: In lateral view, segment IX sharply triangular anteriorly, apex located dorsally; in ventral view anteriorly shallowly oblong. Sternal processes, lateral view, with each apex almost reaching apex of superior appendage, gently tapering along its length, slightly anti-sigmoid, apex truncated; in ventral view, parallel, of similar width throughout its length, slightly bulging at mid-length, apex acute, directed mesad. Tergum X in lateral view trapezoid, shorter than superior appendage, in dorsal view, mesally fused, semi-rectangular with rounded posterior corners, inner margin forming shallow, wide U. Parameres in lateral view complex, multi-branched, anteriorly directed posteromesad, then turning posterodorsad at 90 degree angle, gradually widening with two-pronged apex directed dorsad, mesal branch originating close to 90 degree angle, with wide circular apex; in dorsal view, widest at mid-length, there with large open beak-shaped protrusion directed mesad. Superior appendages, in lateral view, irregularly axe-blade shaped with acute, tooth-shaped apex dorsally, dorsal margin slightly concave, ventral margin convex; in dorsal view, obtuse triangular with apex directed mesally. Inferior appendages, in lateral view, with dorsoanterad orientated slender dorsal branch with hooked, acute apex, ventral branch directed dorsoposterad, wider, shorter, with semi-acute apex, both branches exceeding dorsal margin of sternal process; in ventral view slender, with convex lateral margins, narrow, rounded anteriorly, slightly widening towards mid-length, ventral branch forming two broad posterior processes with blunt apices. Phallus, in lateral view shorter than segment IX, slightly narrowing along its length, posterior end acute; in ventral view irregular.

##### Additional information.

This species was referred to as “sp. 4” in Espeland and Johanson (2010a).

#### Agmina dathioensis sp. nov.

Taxon classificationAnimaliaTrichopteraEcnomidae

9DE6C18A-E878-5A64-8037-0CD979D336D7

http://zoobank.org/AB710193-1B39-4314-8501-7E1143986475

Figs 120–124

##### Diagnosis.

*Agmina dathioensis* sp. nov. is characterised by having long posteriorly orientated sternal processes that are almost parallel-sided along their length. Also *A. rostrata* sp. nov., *A. rougensis* sp. nov., and *A. lata* sp. nov. have long and parallel-sided sternal processes in lateral view. *Agmina dathioensis* sp. nov. is distinguished from all these species by not having heavily sclerotised parameres. *Agmina dathioensis* sp. nov. is furthermore distinguished from *A. rostrata* sp. nov. and *A. lata* sp. nov. in that the sternal processes are orientated ventrally, not posteriorly, and from *A. lata* sp. nov. in the slightly narrower sternal processes in lateral view, and absence of megasetae on the posterior apex of the parameres on the mesal face of the superior appendages. Finally, *A. dathioensis* sp. nov. can be distinguished from *A. rougensis* sp. nov. by the narrower sternal processes in lateral view.

**Figures 120–124.**
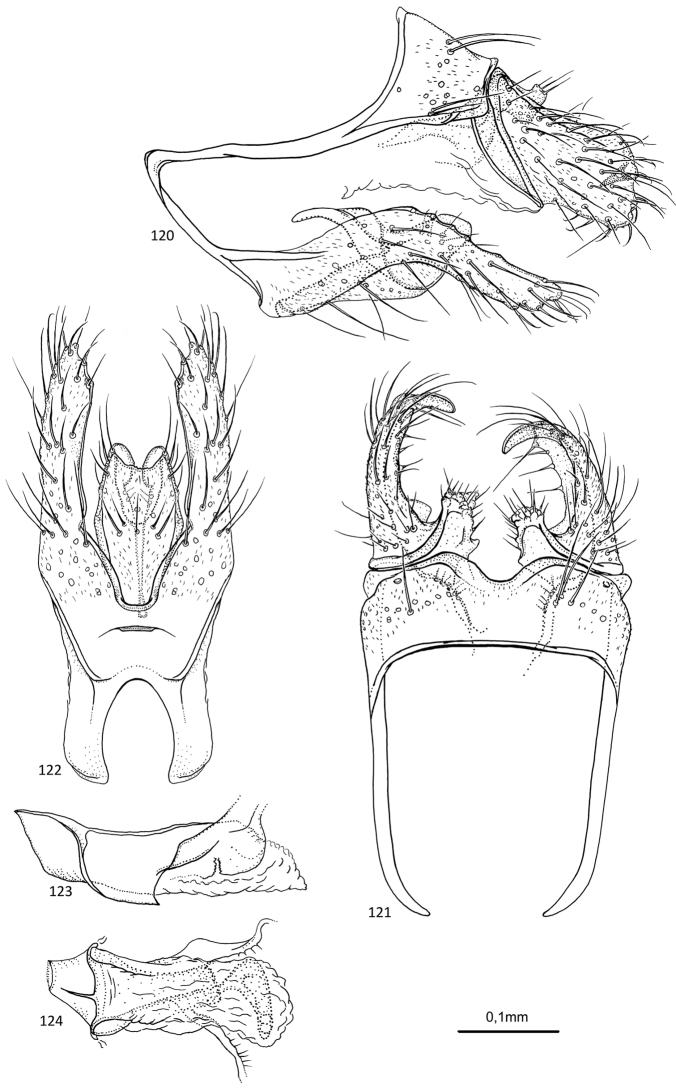
*Agmina dathioensis* sp. nov. male holotype **120** genitalia, left lateral view **121** genitalia, dorsal view **122** genitalia, ventral view **123** phallus, lateral view **124** phallus, ventral view.

##### Etymology.

*Dathioensis*, named for the Dathio River, the type locality of the species.

##### Material examined.

***Holotype***: New Caledonia – **Province Sud** • ♂; Xwé Pemöu Stream, 300 m N bridge over Dathio River at Atè, 6.2 km WNW of Thio; 21.58835S, 166.15117E; 13 m; 29.xi.2003; light trap; loc#056; leg. KA Johanson; MNHN.

##### Measurements.

Fore wing length 3.9 mm (*N* = 1). Total length of genitalia: 0.5 mm.

##### Description.

***Genitalia***: In lateral view, segment IX sharply triangular anteriorly, apex located medially; in ventral view anteriorly ovoid. Sternal processes, lateral view, with each apex almost reaching apex of superior appendage, slightly wider anteriorly, curving downwards, apex blunt forming three small lobes; in ventral view, long, robust, parallel, of equal with along their length, apex acute directed posteriorly. Tergum X in lateral view trapezoid, shorter than superior appendage, in dorsal view, mesally fused with wide bridge, wide rounded lobe, inner margin forming very shallow U. Parameres starting at tergum X, in lateral view complex folded structure with truncated apex exceeding dorsal margin of superior appendage; in dorsal view, slender, hardly discernible structure. Superior appendages, in lateral view, irregularly quadrilateral, apex, wide, truncated, almost twice the length of tergum X; in dorsal view relatively long, slender, slightly curving mesad, narrowing along its length, long acute apex sharply curving mesad. Inferior appendages, in lateral view, with dorsoanterad orientated dorsal branch, initially wider, then tapering towards apex, ventral branch with wide, club-shaped apex, both branches exceeding dorsal margin of sternal process, main structure exceeding ventral margin of superior appendage; in ventral view relatively short, with convex lateral margins, ventral branch forming two broad posterior processes with blunt apices. Phallus, in lateral view shorter than segment IX, almost straight; in ventral view irregular, with long, narrow lateral processes at posterior end.

##### Additional information.

This species was referred to as “sp. 32” in Espeland and Johanson (2010a).

#### Agmina rougensis sp. nov.

Taxon classificationAnimaliaTrichopteraEcnomidae

C087431C-7036-539E-A82F-4FDE650028B3

http://zoobank.org/C8567268-ECAA-441A-8741-11316F2B1E4D

Figs 125–129

##### Diagnosis.

*Agmina rougensis* sp. nov. is characterised by having long ventrally orientated sternal processes that are almost parallel-sided along their length. Also *A. dathioensis* sp. nov., *A. rostrata* sp. nov. and *A. lata* sp. nov. have long and parallel-sided sternal processes in lateral view, and *A. rougensis* sp. nov. is particularly similar to *A. dathioensis* sp. nov. by the ventral orientation of the sternal processes. *Agmina rougensis* sp. nov. is distinguished from *A. rostrata* sp. nov., *A. dathioensis* sp. nov., and *A. lata* sp. nov. by having a much more slender sternal processes in lateral view.

**Figures 125–129.**
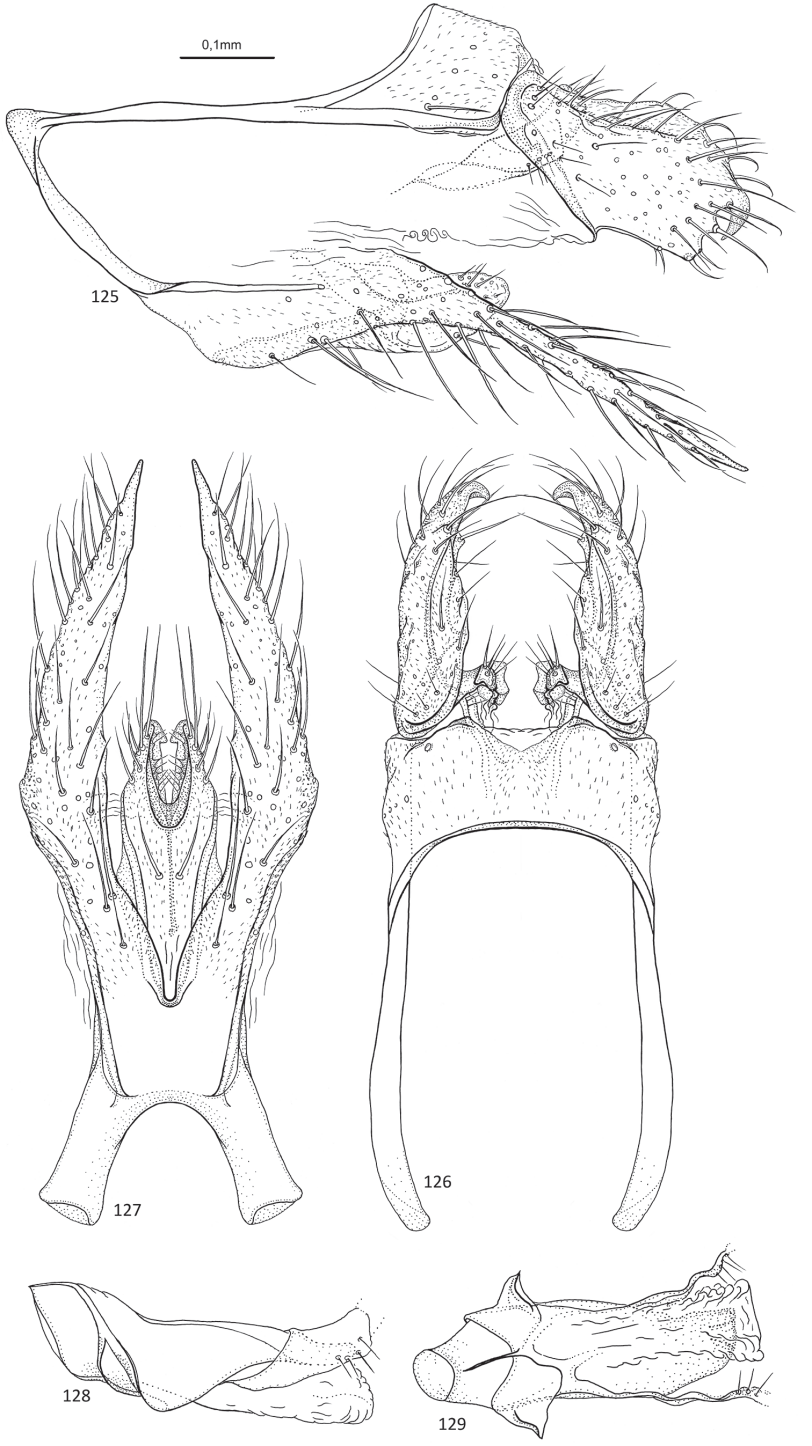
*Agmina rougensis* sp. nov. male holotype **125** genitalia, left lateral view **126** genitalia, dorsal view **127** genitalia, ventral view **128** phallus, lateral view **129** phallus, ventral view.

##### Etymology.

*Rougensis*, named for Rivière Rouge, the type locality of the species.

##### Material examined.

***Holotype***: New Caledonia – **Province Sud** • ♂; Plaine des Gaïacs, Rivière Rouge, 14.2 km NW summit of Mt. Rouge, 50 m upstream road RT1 Nouméa-Koné; 20°31.573'S, 164°46.690'E; 23 m; 2.i.2004; light trap; loc#104; leg. KA Johanson; MNHN.

##### Measurements.

Fore wing length 4.0 mm (*N* = 1). Total length of genitalia: 0.9 mm.

##### Description.

***Genitalia***: In lateral view, segment IX sharply triangular anteriorly, apex located dorsally; in ventral view anteriorly U-shaped. Sternal processes, lateral view, with each apex exceeding apex of superior appendage, anterior half wide, tapering along its length, posterior half, long, slender, spine like with acute apex; in ventral view, robust, twice as long as inferior appendage, tapering along their length, converging, apex acute. Tergum X in lateral view quadratic with mesoanterad corner drawn out, equally long as high, in dorsal view, mesally fused with wide bridge, wide rounded lobe with two short, tooth-like protrusion, inner margin forming very shallow, wide V. Parameres starting at tergum X, in lateral view, short structure approx. four times longer than wide, with truncated apex barely exceeding the dorsal margin of superior appendage; in dorsal view, slender, hardly discernible structure. Superior appendages, in lateral view, irregular posterior half wider than anterior half, apex with claw-like process dorsally, directed ventrad, more than twice the length of tergum X; in dorsal view relatively long, slender, slightly converging towards apex, narrowing along its length, short, acute, claw-like apex sharply curving mesad. Inferior appendages, in lateral view, with dorsoanterad orientated dorsal branch, initially wider, then tapering towards apex, ventral branch with wide, club-shaped apex, ventral branch exceeding dorsal margin of sternal process, main structure slightly exceeding ventral margin of superior appendage; in ventral view narrow anteriorly, widening until mid-length, then straight, slightly narrowing, with ventral branch forming two slightly narrowing processes with rounded apex and tooth orientated mesad. Phallus, in lateral view much shorter than segment X, tubular, almost straight; in ventral view tubular with anterior triangular lateral lobes.

##### Additional information.

This species was referred to as “sp. 44” in Espeland and Johanson (2010a).

#### Agmina viklundi sp. nov.

Taxon classificationAnimaliaTrichopteraEcnomidae

003233B0-E07A-5457-9FC2-0D032CDA5FC0

http://zoobank.org/02F5DAA3-FDE6-4376-87C5-61BF25805FBC

Figs 130–134

##### Diagnosis.

In ventral view *A. viklundi* sp. nov. is similar to *A. complexa* sp. nov. in the long, broad and slightly diverging sternal processes. In lateral view, *A. viklundi* sp. nov. is easily distinguished from *A. complexa* sp. nov. by the convex ventral margin of the sternal processes, which is straight in *A. complexa* sp. nov. Furthermore, in lateral view the superior appendages in *A. viklundi* sp. nov. are wide and rounded posteriorly, while in *A. complexa* sp. nov. these are strongly narrowing apically.

**Figures 130–134.**
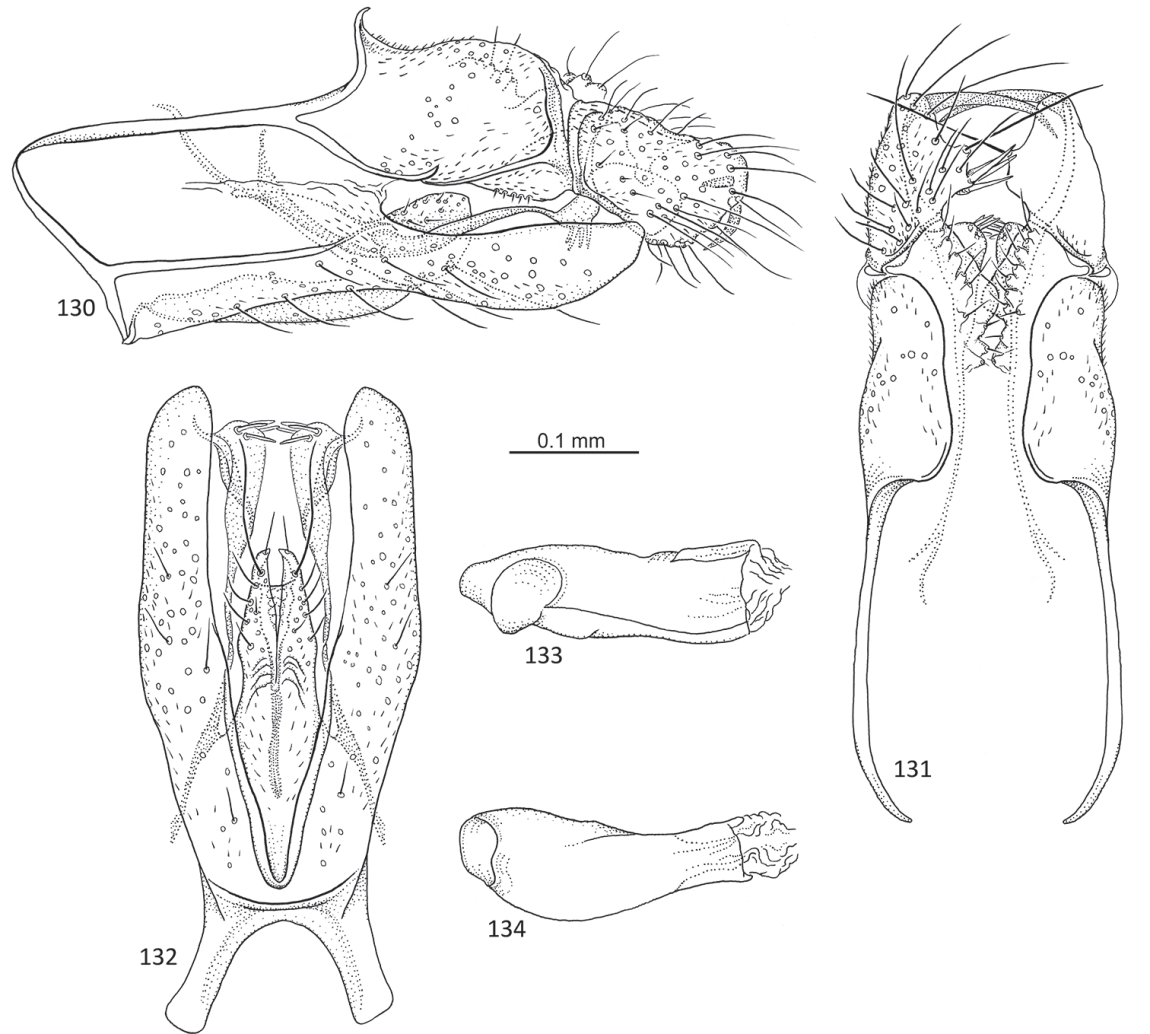
*Agmina viklundi* sp. nov. male holotype **130** genitalia, left lateral view **131** genitalia, dorsal view **132** genitalia, ventral view **133** phallus, lateral view **134** phallus, ventral view.

##### Etymology.

*Viklundi*, named for one of the collectors of the holotype, Mr. Bert Viklund.

##### Material examined.

***Holotype***: New Caledonia – **Province Sud** • ♂; Mt. Panié, Riv. Padyéém; 20°34.122'S, 164°48.147'E; 400 m; 22–28.xi.2001; Malaise trap; 22–28.xi.2001; loc#146 (16-2001); leg. KA Johanson, T Pape & B Viklund; MNHN.

##### Measurements.

Fore wing length 3.5 mm (*N* = 1). Total length of genitalia: 0.6 mm.

##### Description.

***Genitalia***: In lateral view, segment IX triangular anteriorly, apex located medially; in ventral view anteriorly U-shaped. Sternal processes, lateral view, with knife-shaped with anterior half slightly narrowing, posterior end slightly wider, apex rounded; in ventral view, robust, long, of equal width along their length, apex rounded, anterior half diverging, posterior half parallel. Tergum X mug-shaped, in lateral view longer than high; in dorsal view, mesally well-separated, much longer than wide, rounded apex. Parameres in lateral view long, slender, curving upwards, small dorsal lobe at mid-length, apex club-shaped with megasetae directed ventrad, then drawn out into long acuminate process orientated dorsad, barely reaching superior appendage; in dorsal view, separated, initially converging, second half diverging widely, apex club-shaped with megasetae directed mesad. Superior appendages, in lateral view, not exceeding the length of tergum X, irregularly quadrilateral with rounded apex, claw-like process mesally on posterior margin, directed anteromesad; in dorsal view short, stout, rectangular process with large megasetae orientated mesad at mid-length of inner margin, rounded apex with long, curving, spine-like process orientated mesad. Inferior appendages, in lateral view, forming a single rounded, club-shaped structure, gently curving upwards; in ventral view slender, with slightly undulating convex lateral margins, two slightly converging, posterior processes slightly narrowing along their length, with apex directed posterolaterad. Phallus, in lateral view much shorter than segment IX, tubular, straight; in ventral view slightly wider anteriorly, tubular.

##### Additional information.

This species was referred to as “sp. 38” in Espeland and Johanson (2010a).

#### Agmina lata sp. nov.

Taxon classificationAnimaliaTrichopteraEcnomidae

338FC216-0353-5A6B-8F93-B820136B8B0D

http://zoobank.org/CFC62D0F-AF06-46DE-94DB-3BA8F2647E35

Figs 135–139

##### Diagnosis.

*Agmina lata* sp. nov. is characterised by having long posteriorly orientated sternal processes that are almost parallel-sided along their length. Also *A. dathioensis* sp. nov., *A. rostrata* sp. nov. and *A. rougensis* sp. nov. have long and parallel-sided sternal processes in lateral view, but in *A. dathioensis* sp. nov. and *A. rougensis* sp. nov. the sternal processes are orientated ventrally, not posteriorly. It is distinguished from *A. rostrata* sp. nov. by the absence of heavily sclerotised parameres inside the superior appendages.

**Figures 135–139.**
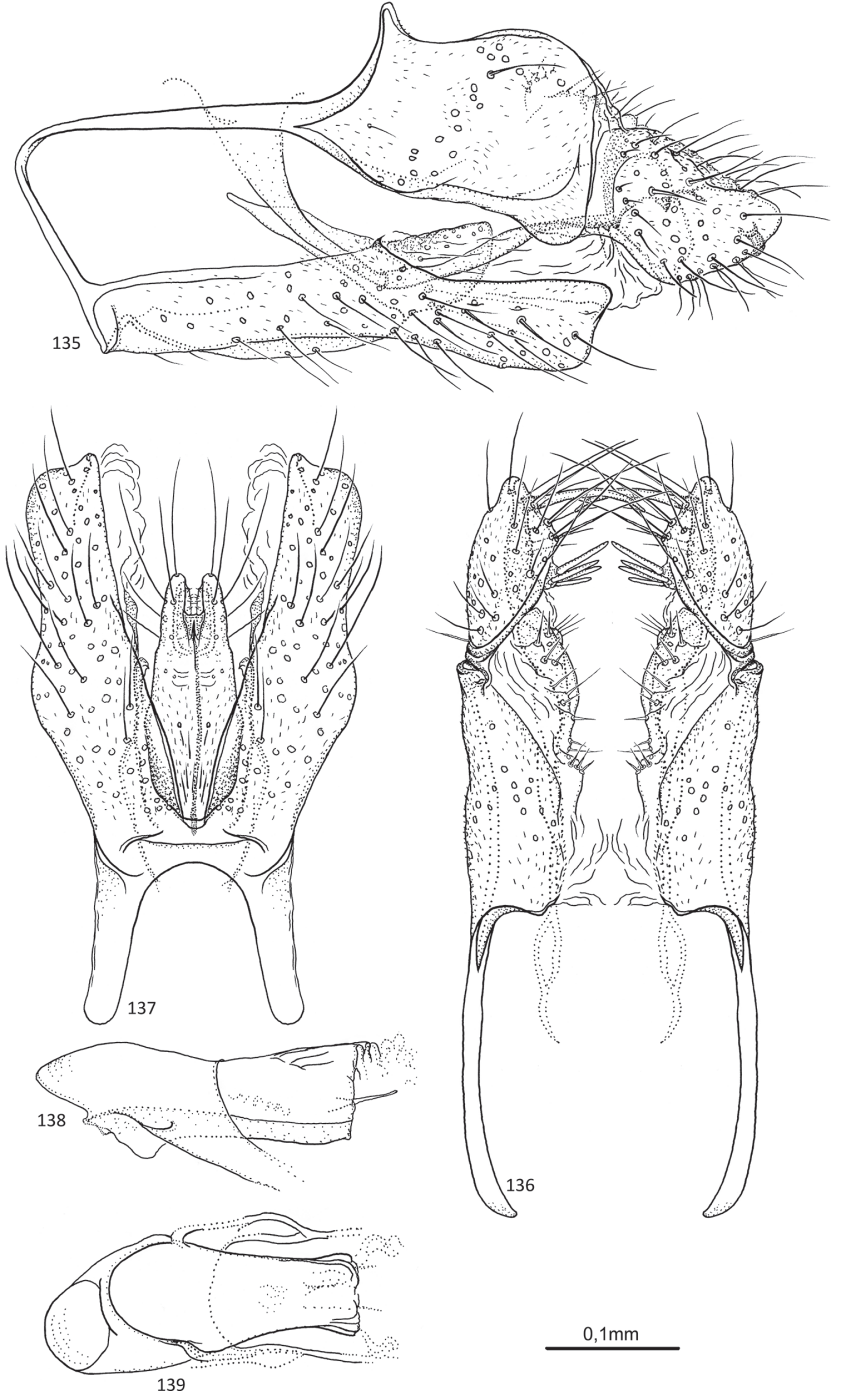
*Agmina lata* sp. nov. male holotype **135** genitalia, left lateral view **136** genitalia, dorsal view **137** genitalia, ventral view **138** phallus, lateral view **139** phallus, ventral view.

##### Etymology.

*Lata*, from Latin, meaning wide. Referring to the wide sternal process in lateral view.

##### Material examined.

***Holotype***: New Caledonia – **Province Sud** • ♂; Réserve spéciale de faune de la haute Yaté, along road on southern part of Marais de la Rivière Blanche, stream draining to Marais de la Rivière Blanche, 2.25 km SW Pont Pérignon; 180 m; 6–16.xi.2003; Malaise trap; loc#010a; leg. KA Johanson; MNHN.

##### Measurements.

Fore wing length 4.0 mm (*N* = 1). Total length of genitalia: 0.6 mm.

##### Description.

***Genitalia***: In lateral view, segment IX rounded triangular anteriorly, apex located medially; in ventral view anteriorly U-shaped. Sternal processes, lateral view, with long, rectangular, of equal width throughout their length, dorsal margin widely triangular at mid-length, apex truncated; in ventral view, robust, slightly diverging, inner margin straight, outer margin widest before mid-length, apex truncated, slightly pointed mesally. Tergum X large, longer than wide, dorsal and ventral margins sigmoid, anterior margin concave; in dorsal view, mesally separate, longer than wide, posterior half rectangular, anterior part triangular. Parameres in lateral view long, slender, wider anteriorly, curving upwards toward apex forming looped structure not exceeding any margins of the superior appendage; in dorsal view, separated, anterior 2/3 parallel, diverging at posterior 1/3, then largely parallel, apex elongated club-shaped. Superior appendages, in lateral view, triangular, shorter than tergum X, mesal spine close to apex; in dorsal view short, triangular with convex lateral margin, apex rounded, long, thin, curved, spine-like process posteriorly on inner margin, directed mesad. Inferior appendages, in lateral view, forming a single knife-blade-shaped structure, with straight dorsal margin, convex ventral margin with shallow notch on anterior half; in ventral view slender, rounded, triangular at base, widest just before mid-length, then slightly tapering towards apex with two parallel posterior processes with rounded apex. Phallus, in lateral view much shorter than segment IX, tubular; in ventral view wider anteriorly.

##### Additional information.

This species was referred to as “sp. 5” in Espeland and Johanson (2010a).

#### Agmina falx sp. nov.

Taxon classificationAnimaliaTrichopteraEcnomidae

4564EA12-A7E9-5237-8A49-F567162B45D0

http://zoobank.org/53D59901-6E6F-43FB-A715-6CE02233F1F6

Figs 140–145

##### Diagnosis.

This species is easily distinguished from other *Agmina* species in lateral view by the sickle shaped sternal processes, each being slightly curved ventrally and with a sharp apex. It also has a large, claw-like structure apically in the superior appendages and pointed megasetae at the apex of the parameres.

**Figures 140–145.**
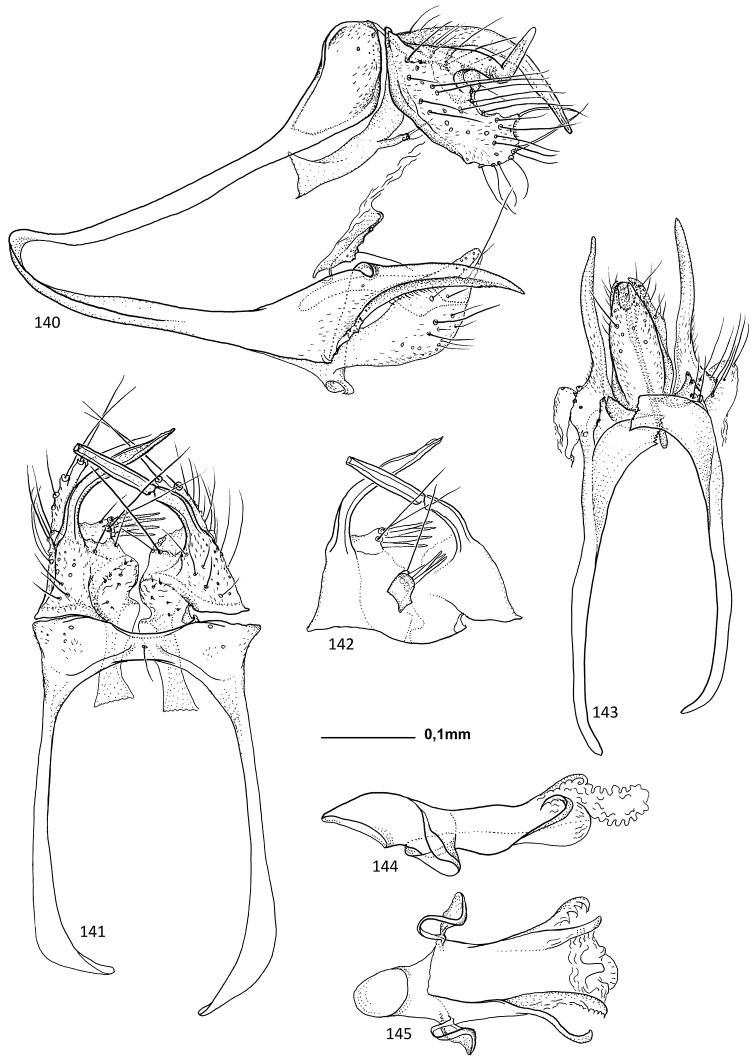
*Agmina falx* sp. nov. male holotype **140** genitalia, left lateral view **141** genitalia, dorsal view **142** superior appendage, underside in dorsal view **143** genitalia, ventral view **144** phallus, lateral view **145** phallus, ventral view.

##### Etymology.

From Latin *falx* (noun, feminine), meaning sickle. Named for the sickle-shaped sternal process in lateral view.

##### Material examined.

***Holotype***: New Caledonia – **Province Sud** • ♂; 2.8 km ENE Bopope, at site where Rivière Kövé Tamè enters Rivière Oua Mendiou, 100 m S RPN2 Koné-Poindimié; 20°54.455'S, 165°06.300'E; 78 m; 14.i.2003; light trap; loc#119; leg. KA Johanson; MNHN.

##### Measurements.

Fore wing length 3.1 mm (*N* = 1). Total length of genitalia: 0.6 mm.

##### Description.

***Genitalia***: In lateral view, segment IX rounded triangular with slightly upturned apex anteriorly, apex located ventrally; in ventral view anteriorly U-shaped. Sternal processes, lateral view, with sickle-shaped, curving downwards posteriorly, on dorsal margin rounded notch at mid-length; in ventral view, slender, parallel, spine-like with acute apex. Tergum X small, oblong with drawn out, ventroanterad corner, wider than long; in dorsal view, mesally fused, short, not much wider than mesal bridge, posterior margins slightly convex. Parameres originating before tergum X, in lateral view tubular, slightly wider anteriorly, then of equal width along its length, thin, tubular, blunt, process on ventral margin before mid-length; in dorsal view, anterior half tubular, nearly parallel, posterior part folding in on itself, apex truncated, mesally with long, straight, megasetae directed mesad. Superior appendages, in lateral view, curving with rounded, slightly dentate apex, dorsally with long, downwards-curving, spine-like process, exceeding main appendage; in dorsal view irregular, equally wide as high with almost straight posterolateral process, with dentate lateral margin, above this with posteromesad curving acute, spine-like process. Inferior appendages, in lateral view, forming single rounded, rhomboid process with rounded apex; in ventral view short, slender with convex margin, apex with two thin spine-like posterior processes with acute apex. Phallus, in lateral view much shorter than segment IX, tubular; in ventral view wider posteriorly, with looped lateral lopes anteriorly.

##### Additional information.

This species was referred to as “sp. 13” in Espeland and Johanson (2010a).

#### Agmina guttata sp. nov.

Taxon classificationAnimaliaTrichopteraEcnomidae

6C4B7109-3F43-52B7-B925-4309DCDC3140

http://zoobank.org/CAB0DB4A-B613-479E-A484-79BA495DF684

Figs 146–150

##### Diagnosis.

In ventral view, the inferior appendages of *Agmina guttata* sp. nov. form a narrow plate with a posteroapical incision laterally flanged by a row of small setae, similar to those of *A. complexa* sp. nov., *A. amieuensis* sp. nov., and *A. spina* sp. nov. *Agmina guttata* sp. nov. also have very short sternal processes in lateral view, as also present in *A. amieuensis* sp. nov. and *A. spina* sp. nov. *Agmina guttata* sp. nov. is distinguished from the similar species by the shape of the sternal process in ventral view, which is oval, almost drop-shaped.

**Figures 146–150.**
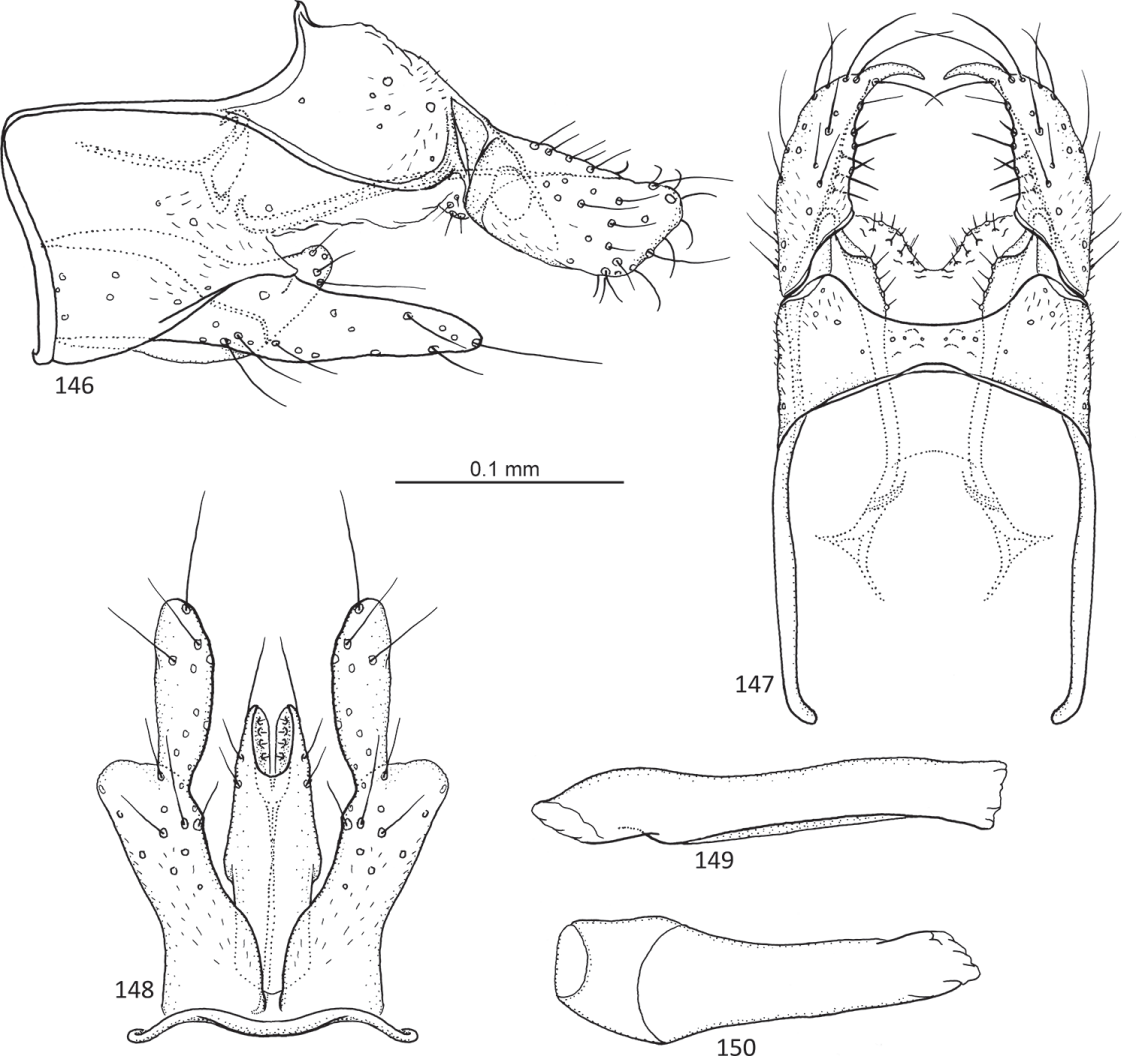
*Agmina guttata* sp. nov. male holotype **146** genitalia, left lateral view **147** genitalia, dorsal view **148** genitalia, ventral view **149** phallus, lateral view **150** phallus, ventral view.

##### Etymology.

*Guttata*, from Latin, meaning drop-shaped. Referring to the shape of the sternal process in ventral view.

##### Material examined.

***Holotype***: New Caledonia – **Province Sud** • ♂; Haute Yaté fauna reserve, 1760 m S bridge Pont Perignon, 50 m upstream bridge over stream; 22.14954S, 166.701211E; 180 m; 14.xii.2003–13.i.2004; Malaise trap; loc#081; leg. KA Johanson; MNHN.

***Paratypes***: New Caledonia – **Province Sud** • 2 ♂; Réserve spéciale de faune de la haute Yaté, along road on southern part of Marais de la Rivière Blanche, stream draining to Marais de la Rivière Blanche, 2.25 km SW Pont Pérignon; 180 m; 6–16.xi.2003; Malaise trap; loc#010a; leg. KA Johanson; NHRS; • 1 ♂; Rivière Ouanéoue, at bride crossing road to Koghi Mountains, ca. 1.5 km from road RT1 Nouméa-Dumbea; 22°10.861'S, 166°29.531'E; 11.xi.2003; light trap; loc#024a; leg. KA Johanson; NHRS; • 1 ♂; Réserve spéciale de faune de la haute Yaté, along road on southern part of Marais de la Rivière Blanche, stream draining to Marais de la Rivière Blanche, 1.35 km S Pont Pérignon; 22°08.496'S, 166°42.152'E; 180 m; 6–16.xi.2003; Malaise trap; loc#009a; leg. KA Johanson; NHRS; • 1 ♂; Réserve spéciale de faune de la haute Yaté, along road on southern part of Marais de la Rivière Blanche, stream draining to Marais de la Rivière Blanche, 3.7 km SW Pont Pérignon; 22°09.327'S, 166°40.841'E; 180 m; 6–16.xi.2003; Malaise trap; loc#013; leg. KA Johanson; NHRS; • 1 ♂; W slope Mt. Ningua, Kwé Néco Stream, at Camp Jacob, 3.7 km WNW summit of Mt. Ningua, on Boulo-Thio Road, ca. 50 m upstream road; 21°43.613'S, 166°06.567'E; 150 m; 29.xi-12.xii.2003; Malaise trap; loc#054; leg. KA Johanson; NHRS; **Province Nord** • 2 ♂; Mt. Panié, Riv. Padyéém; 400 m; 22–28.xi.2001; Malaise trap; 22–28.xi.2001; 20°34.122'S, 164°48.147'E; loc#146 (16-2001); leg. KA Johanson, T Pape & B Viklund; NHRS; • 1 ♂; Wé Caot Stream, draining NNE side of Mt. Panié, 0.9 km NW Cascade de Tao; 20°33.311'S, 164°48.064'E; 18.xii.2003; light trap; loc#084; leg. KA Johanson; NHRS.

##### Measurements.

Fore wing length 2.4–3.8 mm (*N* = 11). Total length of genitalia: 0.3 mm.

##### Description.

***Genitalia***: In lateral view, segment IX almost rectangular, apex located dorsally; in ventral view anteriorly slightly undulating, almost straight. Sternal processes, lateral view, with straight, gently tapering along their length, apex semi-acute; in ventral view, anterior half robust, diverging, of equal width throughout their length, abruptly narrowing slightly posteriorly of mid-length, posterior part club-shaped with rounded apex. Tergum X semi-rectangular with anterior margin concave and posterior margin convex; in dorsal view, mesally fused, forming rounded lobe with shallow, wide notch at apex. Parameres originating before tergum X, in lateral view long, slender, initially trifurcated, then slender, tubular directed posterodorsad, forming complex looped structure at apex; in dorsal view, separated, initially converging, then diverging, straight, slightly widening along their length, thin, whip-like apex curving anterolaterad. Superior appendages, in lateral view, rounded, rectangular with truncated apex, as long as tergum X; in dorsal view parallel, curving mesally towards apex, anterior end triangular, lateral margin convex, mesal margin straight, claw-like mesoanterad directed process at apex. Inferior appendages, in lateral view, forming single slightly narrowing process with rounded apex exceeding lateral margin of sternal process; in ventral view slender, anterior end widely triangular, lateral margins parallel anteriorly, slightly converging posteriorly, apex with two spine-like, thin posterior processes. Phallus, in lateral view as long as segment IX, tubular, straight; in ventral view wider anteriorly, tubular.

##### Additional information.

This species was referred to as “sp. 31” in Espeland and Johanson (2010a).

#### Agmina amieuensis sp. nov.

Taxon classificationAnimaliaTrichopteraEcnomidae

C6158FE2-FC18-5D8E-9269-FD9E78406BB8

http://zoobank.org/017B80BA-5FCD-4F1F-A885-37971CA3AD99

Figs 151–155

##### Diagnosis.

In ventral view, the inferior appendages of *Agmina amieuensis* sp. nov. form a narrow plate with a posteroapical incision laterally flanged by a row of small setae, similar to those of *A. complexa* sp. nov., *A. guttata* sp. nov., and *A. spina* sp. nov. *Agmina amieuensis* sp. nov. also have very short sternal processes in lateral view, as also present in *A. guttata* sp. nov. and *A. spina* sp. nov. *Agmina amieuensis* sp. nov. is distinguished by the shape of the sternal process in ventral view, which is oval, almost drop-shaped.

**Figures 151–155.**
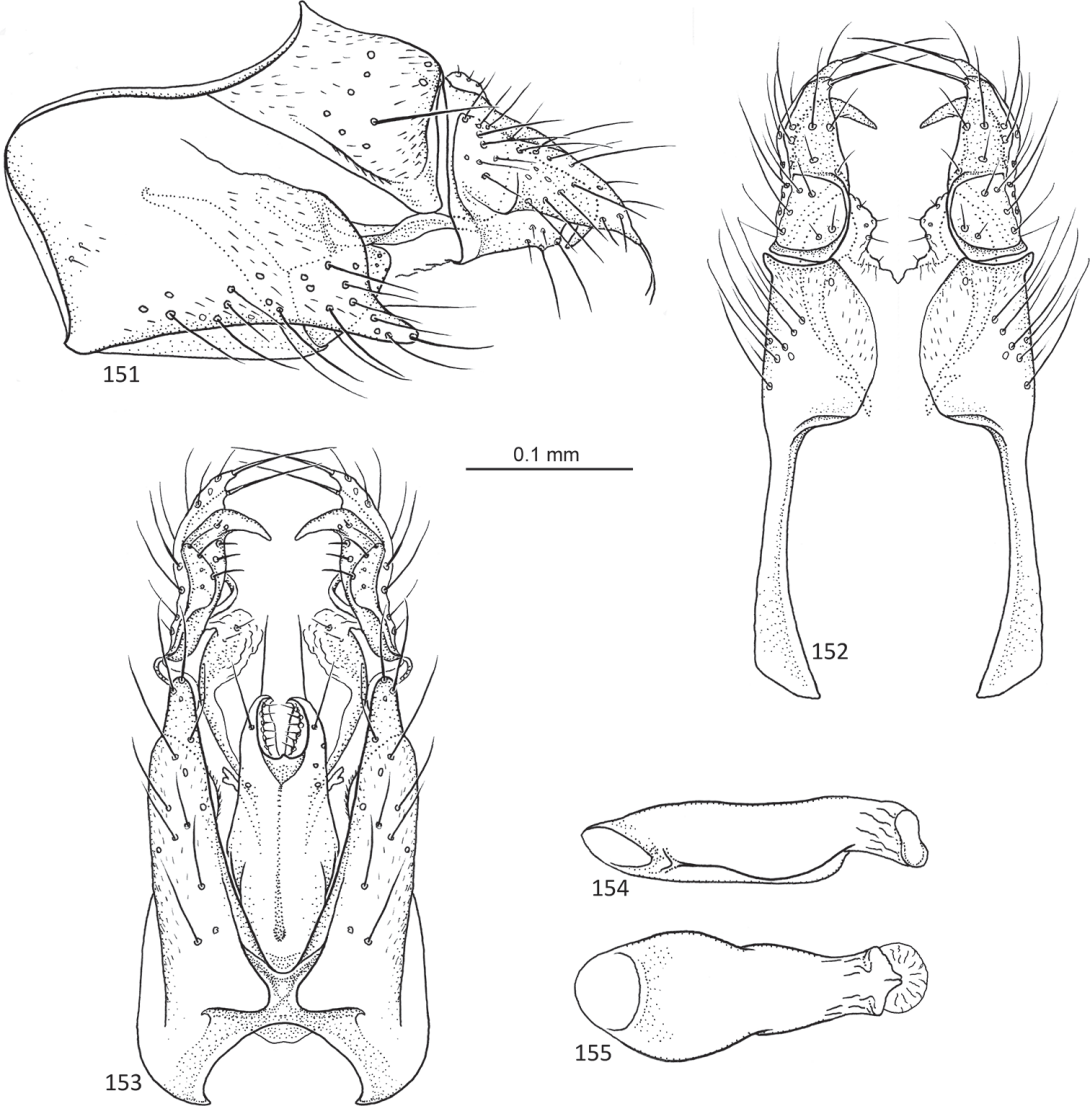
*Agmina amieuensis* sp. nov. male holotype **151** genitalia, left lateral view **152** genitalia, dorsal view **153** genitalia, ventral view **154** phallus, lateral view **155** phallus, ventral view.

##### Etymology.

*Amieuensis*, named for Réserve Spéciale de faune du Col d’Amieu et Table Unio, the type locality of the species.

##### Material examined.

***Holotype***: New Caledonia – **Province Sud** • ♂; Réserve Spéciale de faune du Col d’Amieu et Table Unio, branch of Fa Tööiri Stream; 21°34.844'S, 165°49.677'E; loc#155 (25-2001); Malaise trap; 30.xi-5.xii.2001; leg. KA Johanson, T Pape & B Viklund; MNHN.

***Paratypes***: New Caledonia – **Province Nord** • 1 ♂; Mt. Panié, Riv. Padyéém; 400 m; 22–28.xi.2001; Malaise trap; 22–28.xi.2001; 20°34.122'S, 164°48.147'E; loc#146 (16-2001); leg. KA Johanson, T Pape & B Viklund; NHRS; **Province Sud** • 1 ♂; Plateau de Dogny, source of Dogny River, ca, 100 S of loc 046 and nearly 1.0 km SE summit of Platou; 21.62095S, 165.88072E; 917 m; 25.xi-16.xii.2003; Malaise trap; loc#047; leg. KA Johanson; NHRS; • 1 ♂; Monts des Koghis, ca 300 m S Koghi Restaurant; 22.18288S, 166.50245E; 427 m; 2–16.xi.2003; Malaise trap; loc#003; leg. KA Johanson; NHRS; • 1 ♂; Monts des Koghis, ca 300 m S Koghi Restaurant; 22.18288S, 166.50167E; 417 m; 2–16.xi.2003; Malaise trap; loc#004; leg. KA Johanson; NHRS; • 2 ♂; Monts Kwa Ne Mwa, on road between Nouméa and Yaté, 2.0 km E Pic Mouirange; 22°12.356'S, 166°40.798'E; 220 m; 7–16.xi.2003; Malaise trap; loc#014; leg. KA Johanson; NHRS.

##### Measurements.

Fore wing length 2.8–3.7 mm (*N* = 7). Total length of genitalia: 0.4 mm.

##### Description.

***Genitalia***: In lateral view, segment IX widely bell-shaped, apex located medially; in ventral view anteriorly oval. Sternal processes, lateral view, with broad structure with semi-acute apex located ventrally, ventral margin slightly concave; in ventral view, slightly diverging, tapering along their length, apex rounded. Tergum X trapezoid with concave anterior margin; in dorsal view, mesally separate, longer than wide, inner margin convex, posterior margin slightly concave. Parameres originating at tergum X, in lateral view initially slender, widening to a folded, twisting, sheet-like structure at apex; in dorsal view, slender, gently widening along their length, initially diverging, posterior half converging, apex rounded, club-shaped. Superior appendages, in lateral view, triangular with dorsal margin convex, apex acute directed posteromesad, spine-like mesal process curving downwards with apex directed anteromesad; in dorsal view longer than wide, outer margin sigmoid, inner margin convex, two apices directed postero mesad, mesal process directed anteromesad posteriorly of both apices. Inferior appendages, in lateral view, with bifurcated anterior half, ventral process running parallel with, and exceeding ventral margin of sternal process, posterior part forming a single narrowing lobe with rounded apex exceeding posterior margin of sternal process; in ventral view posterior half forming lobe-like structure narrowing towards rounded apex. Phallus, in lateral view almost as long as segment IX, tubular, straight; in ventral view wider anteriorly, tubular.

##### Additional information.

This species was referred to as “sp. 18” in Espeland and Johanson (2010a).

#### Agmina spina sp. nov.

Taxon classificationAnimaliaTrichopteraEcnomidae

FE930D21-6CBD-5B68-BF3F-142D00BE836E

http://zoobank.org/442398D4-AA9C-4DB0-BA9E-C3D6454D5290

Figs 156–160

##### Diagnosis.

In ventral view, the inferior appendages of *Agmina spina* sp. nov. form a narrow plate with a posteroapical incision laterally flanged by a row of small setae, similar to that of *A. complexa* sp. nov., *A. guttata* sp. nov., and *A. amieuensis* sp. nov. *Agmina spina* sp. nov. also have very short sternal processes in lateral view, as also present in *A. guttata* sp. nov. and *A. amieuensis* sp. nov. *Agmina spina* sp. nov. is distinguished from the other species by the narrow and tapering superior appendages in lateral view.

**Figures 156–160.**
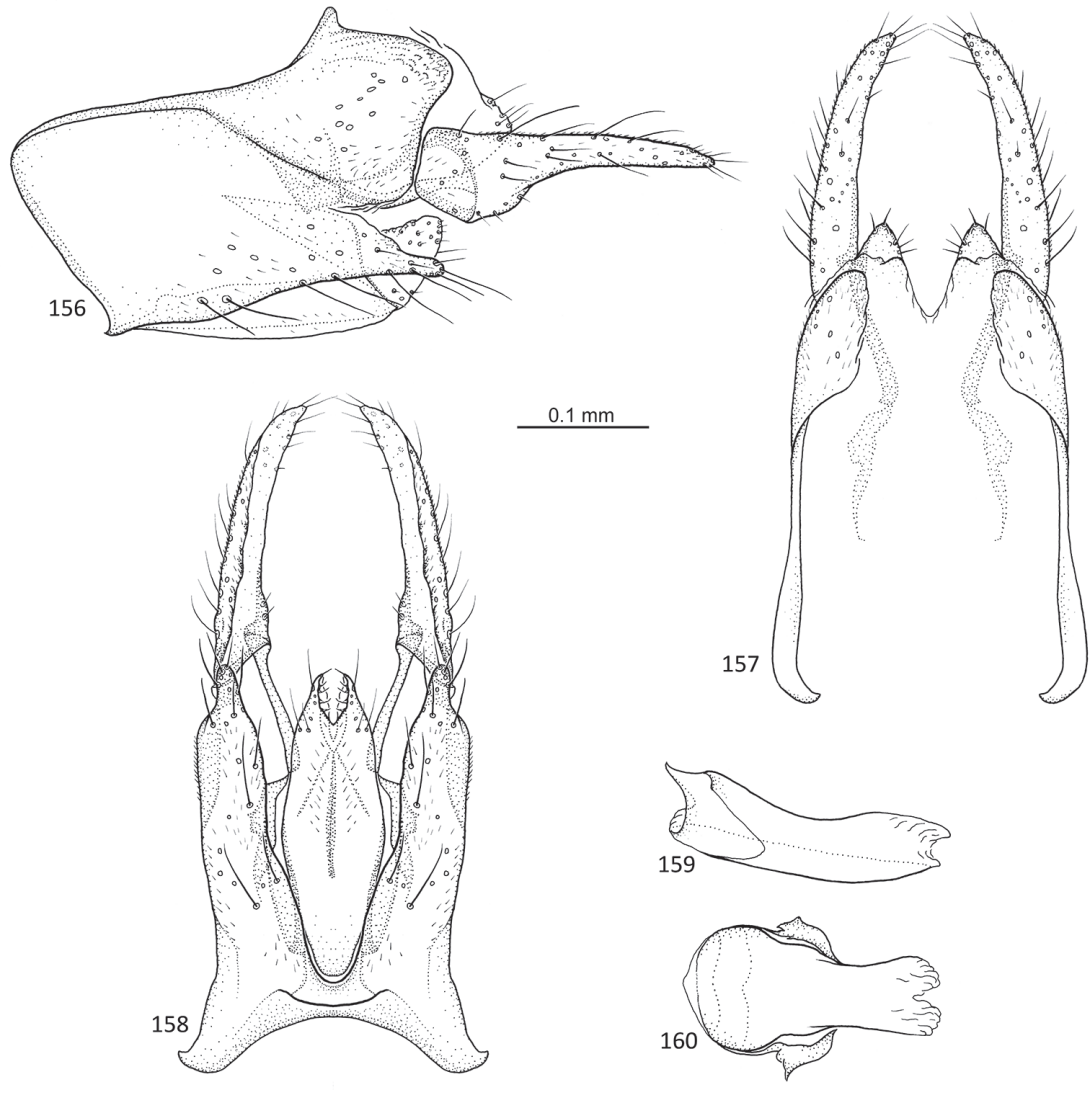
*Agmina spina* sp. nov. male holotype **156** genitalia, left lateral view **157** genitalia, dorsal view **158** genitalia, ventral view **159** phallus, lateral view **160** phallus, ventral view.

##### Etymology.

From Latin *spina* (noun, feminine), meaning spine. Named for the spine-like shape of the superior appendage.

##### Material examined.

***Holotype***: New Caledonia – **Province Sud** • ♂; Plateau de Dogny; 21°37.000'S, 165°52.500'E; 846 m; loc#145 (15-2001); Malaise trap; 18–21.xi.2001; leg. KA Johanson, T Pape & B Viklund; MNHN.

##### Measurements.

Fore wing length 4.1 mm (*N* = 1). Total length of genitalia: 0.5 mm.

##### Description.

***Genitalia***: In lateral view, segment IX widely bell-shaped, apex located medially; in ventral view anteriorly oval. Sternal processes, lateral view, with tapering along their length, ventral margin almost straight, apex slender, rounded; in ventral view, slightly diverging, of nearly equal width throughout their length, abruptly narrowing towards rounded apex. Tergum X almost quadratic with concave anterior and posterior margin, drawn-out anteroventral corner; in dorsal view, widely mesally separated, twice as long as wide, outer margins convex, inner margins straight, but slightly undulating. Parameres originating at tergum X, in lateral view forming complex structure with looped apex; in dorsal view, slender, anterior part slightly converging, posterior part diverging, apex not readily visible. Superior appendages, in lateral view, exceeding length of tergum X, wider at base, then slender, spine-like, straight, with acute apex; in dorsal view long, slender, slightly curving, converging towards acute apex. Inferior appendages, in lateral view, with bifurcated anterior half, ventral process straight, exceeding ventral margin of sternal process, posterior part forming a single narrowing lobe with rounded apex directed posteromesad, exceeding posterior margin of sternal process; in ventral view slender with convex margin, two acute, thin, posterior processes at apex. Phallus, in lateral view not exceeding length of segment IX, tubular, slightly curving; in ventral view wider anteriorly, with irregular lateral processes at mid-length.

##### Additional information.

This species was referred to as “sp. 40” in Espeland and Johanson (2010a).

#### Agmina complexa sp. nov.

Taxon classificationAnimaliaTrichopteraEcnomidae

F2CBF558-96A9-528A-BAE0-0DBE601CBB30

http://zoobank.org/C8FC9FA5-E342-4FA7-9C98-F45945CE312A

Figs 161–165

##### Diagnosis.

In ventral view, the inferior appendages of *Agmina complexa* sp. nov. form a narrow plate with a posteroapical incision laterally flanged by a row of small setae, similar to those of *A. spina* sp. nov., *A. guttata* sp. nov., and *A. amieuensis* sp. nov. *Agmina complexa* sp. nov. are easily separated from the above similar species by the much longer and almost oval sternal process in lateral view, and the superior appendages are strongly modified and form a pair of very large, anteriorly curving hooks. *Agmina amplexa* sp. nov. also have strongly modified superior appendages but *A. amplexa* sp. nov. has three pairs of more or less mesally orientated hooks instead of a single pair of hooks that are directed anterad in *A. complexa* sp. nov., as seen in dorsal view.

**Figures 161–165.**
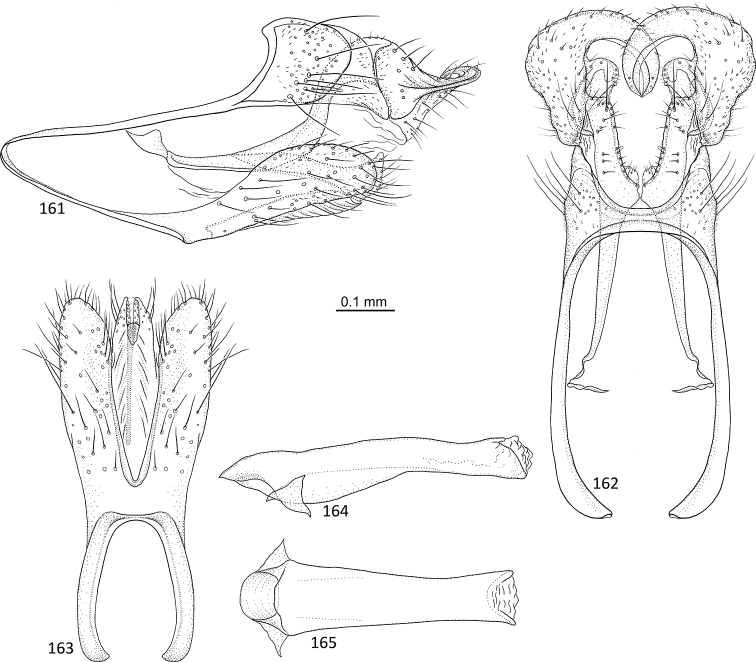
*Agmina complexa* sp. nov. male holotype **161** genitalia, left lateral view **162** genitalia, dorsal view **163** genitalia, ventral view **164** phallus, lateral view **165** phallus, ventral view.

##### Etymology.

Named for the complex shape of the superior appendage.

##### Material examined.

***Holotype***: New Caledonia – **Province Nord** • ♂; Mt. Panié, stream at camp; 20.58139S, 164.76444E; 1310 m; 9.xii.2003; Malaise trap; loc#074; leg. KA Johanson; MNHN.

***Paratype***: New Caledonia – **Province Nord** • 1 ♂; Mt. Panié, stream at camp; 20.58167S, 164.76472E; 1311 m; 9.xii.2003; Malaise trap; loc#073; leg. KA Johanson; NHRS.

##### Measurements.

Fore wing length 3.5–4.3 mm (*N* = 2). Total length of genitalia: 0.8 mm.

##### Description.

***Genitalia***: In lateral view, segment IX narrowly triangular, apex located medially; in ventral view anteriorly elongated, U-shaped. Sternal processes, lateral view, with club-shaped, dorsal margin convex, ventral margin straight, apex rounded; in ventral view, robust, slightly diverging, equally wide along their length, apex truncated. Tergum X irregular quadrilateral with concave anterior margin, convex posterior margin, lateral margins almost straight; as wide as high; in dorsal view, mesally fused, triangular with acute apex. Parameres slender, starting before tergum X, in lateral view sigmoid, widening at apex with concave posterior margin; in dorsal view, separated, long, slender, straight, widening along their length, posterior half slightly wider anteriorly, then equally wide along their length, posteriorly abruptly curving lateroanterad, apex hook-shaped directed anterad. Superior appendages, in lateral view, initially triangular, greatly narrowing forming ridged, greatly curved, narrow process with acute apex directed mesad; in dorsal view forming large hook-like structures, with apices greatly curving, directed anteromesad, crossing mesally. Inferior appendages, in lateral view, with posterad orientated single branch, straight with truncate apex with dorsad directed tooth dorsally, ventral margin and apex exceeding margin of sternal process; in ventral view very slender, lateral margins convex, acute, narrow posterior processes at apex. Phallus, in lateral view tubular, straight; in ventral view slightly wider anteriorly, with triangular lateral lobes, tubular.

##### Additional information.

This species was referred to as “sp. 22” in Espeland and Johanson (2010a).

### Species group 5, *dognyensis*-group

Included species in this group are: *Agmina dognyensis* sp. nov., *A. mana* sp. nov., *A. anterohamata* sp. nov., *A. joycei* Ward & Schefter, 2000, and *A. curvatacua* sp. nov. This is a strongly supported monophyletic group based on DNA data (Espeland and Johanson 2010a), but no morphological characters are unique for the group.

#### Agmina dognyensis sp. nov.

Taxon classificationAnimaliaTrichopteraEcnomidae

27F6CD6F-E30B-586C-ADFF-4491F9D6DCEC

http://zoobank.org/B8A3EC2D-F3B5-49D8-A209-E558EB58BB66

Figs 166–170

##### Diagnosis.

*Agmina dognyensis* sp. nov. is unique in the combination of having a long, narrow, almost straight superior appendage in lateral view together with a narrow and uniformly tapering sternal processes having a dorsad bend at mid-length. Being armed with a series of megasetae on the posterior part of both parameres is also unique for this species.

**Figures 166–170.**
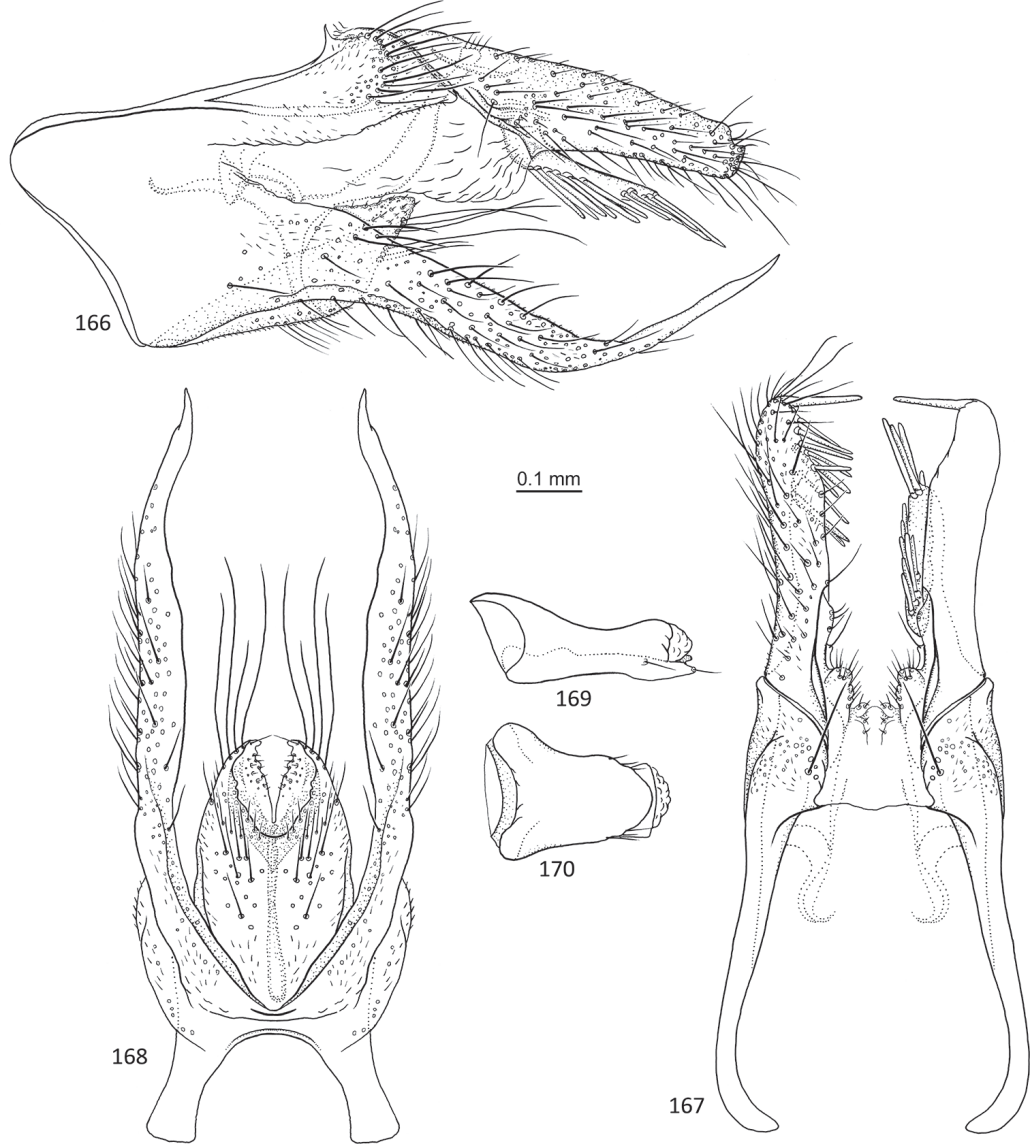
*Agmina dognyensis* sp. nov. male holotype **166** genitalia, left lateral view **167** genitalia, dorsal view **168** genitalia, ventral view **169** phallus, lateral view **170** phallus, ventral view.

##### Etymology.

*Dognyensis*, named for Plateau de Dogny, the type locality of the species.

##### Material examined.

***Holotype***: New Caledonia – **Province Sud** • ♂; Plateau de Dogny, source of Dogny River, ca. 900 m SE summit of Plateau de Dogny; 21.61917S, 165.88072E; 919 m; 25.xi-16.xii.2003; Malaise trap; loc#046; leg. KA Johanson; MNHN.

***Paratype***: New Caledonia – **Province Sud** • 1 ♂; Plateau de Dogny, source of Dogny River, ca. 1.2km SE summit of Platou, ca. 200 m from waterfall; 21.62067S, 165.88290E; 915 m; 25.xi-16.xii.2003; Malaise trap; loc#048; leg. KA Johanson; NHRS.

##### Measurements.

Fore wing length 4.1–6.0 mm (*N* = 2). Total length of genitalia: 1.2 mm.

##### Description.

***Genitalia***: Total length 1.2 mm. In lateral view, segment IX low, long, rounded anteriorly, apex located slightly above mid-height of genitalia; in ventral view, anterior incision widely and deeply U-shaped. Sternal processes, lateral view, with very long, basally wide, uniformly narrowing and slightly Z-shaped, apex long, smooth, pointed posterodorsally; in ventral view, undulating and posteriorly orientated along their length, parallel-sided except narrowing at apex. Tergum X long, narrowly triangular, with convex posterior and concave dorsal margin with dorsal process at mid-length; in dorsal view, mesally separate, mesal margin convex. Parameres weakly developed; anterior part slender in lateral view, starting well below and before tergum X, short anterior part orientated posteriorly before slightly curving dorsally below mid-length of tergum X; dividing into short dorsal branch fused with dorsobasal part of superior appendage, and robust ventral branch exceeding ventral part of superior appendage and with long row of long, posteriorly orientated megasetae; in dorsal view, separated and S-shaped at base, almost invisible along tergum X and superior appendage, except ventral branch and its mesally orientated megasetae. Superior appendages, in lateral view, simple, slender, with parallel, almost straight dorsal and ventral margins; apex with small right-angled incision; in dorsal view narrow, weakly diverging from mid-length; apex rounded. Inferior appendages, in lateral view, with wide, posterodorsal corner produced into short, narrow process; ends near basis of superior appendages; in ventral view short, almost oval shield with both posterodorsal and posteroventral corners separated mesally by U-shaped ventral and V-shaped incision. Phallus, in lateral view straight, approx. as long as tergum X, posteroventrally slightly produced posteriorly; in ventral view basis wide, gradually narrowing towards rounded apex.

##### Additional information.

This species was referred to as “sp. 37” in Espeland and Johanson (2010a).

#### Agmina mana sp. nov.

Taxon classificationAnimaliaTrichopteraEcnomidae

49E21281-62B1-5A3A-A029-4842A2108ACB

http://zoobank.org/7B1B9DE5-840F-4CB2-8609-11A396666D22

Figs 171–174

##### Diagnosis.

*Agmina mana* sp. nov. is unique in the combination of having a long, narrow, and bifurcated superior appendage in lateral view together with a narrow and uniformly tapering sternal processes having a uniform dorsal curving along their length. Being armed with numerous megasetae on both dorsal and ventral branches of the superior appendage is also unique for this species.

**Figures 171–174.**
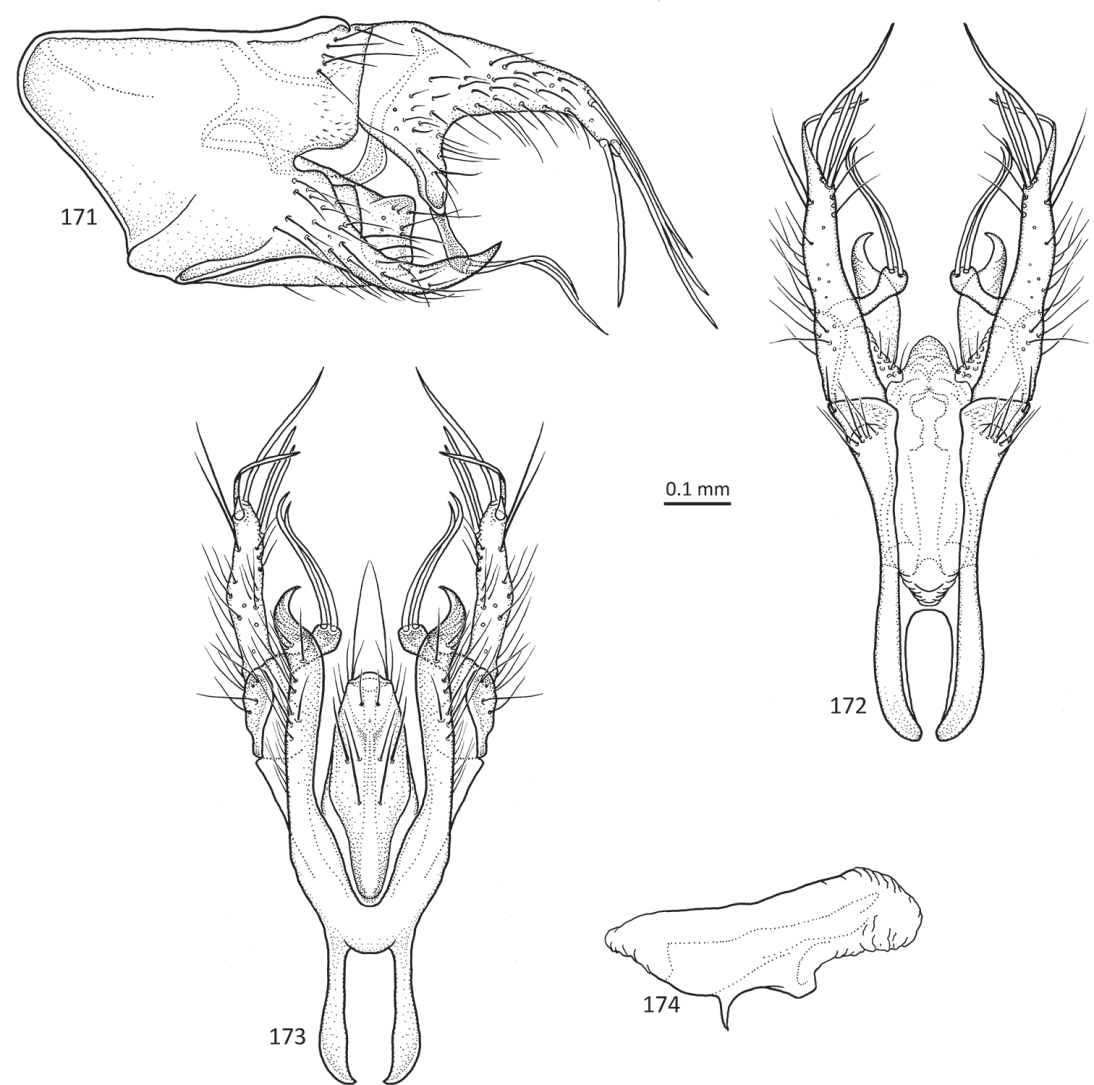
*Agmina mana* sp. nov. male holotype **171** genitalia, left lateral view **172** genitalia, dorsal view **173** genitalia, ventral view **174** phallus, lateral view.

##### Etymology.

From Latin *manus* (noun, masculine) meaning hand. Named for the superior appendages shaped like an open human hand in mesal view.

##### Material examined.

***Holotype***: New Caledonia – **Province Nord** • ♂; Mt. Panié, Riv. Padyéém; 20°34.122'S, 164°48.147'E; loc#146 (16-2001); 400 m; Malaise trap; 22–28.xi.2001; leg. KA Johanson, T Pape & B Viklund; MNHN.

##### Measurements.

Fore wing length 5.1 mm (*N* = 1). Total length of genitalia: 0.9 mm.

##### Description.

***Genitalia***: Total length 0.9 mm. In lateral view, segment IX low, long, rounded anteriorly, apex located dorsally in genitalia; in ventral view anterior incision narrowly deeply rectangularly shaped. Sternal processes, lateral view, with basally wide, sharply narrowing into dorsally curving, slender process, apex pointed; in ventral view, parallel-sided along their length before curving slightly laterally at four-fifth their lengths, apex pointing mesally. Tergum X small, triangular, with straight posterior and dorsal margin; in dorsal view, mesally separate, mesal margin not produces mesally. Parameres weakly developed; anterior part slender in lateral view, starting well below and before tergum X, short anterior part orientated anteriorly before looping posteriorly and with basal half running along dorsal margin of superior appendages, bending dorsally at mid-length, distal half slightly undulating dorsally, not exceeding superior appendages posteriorly; in dorsal view, fused at base, before separated and orientated posteriorly; Superior appendages, in lateral view, long, shaped like an open human hand in mesal view; divided into dorsal, posteriorly orientated branch and ventrally orientated branch near basis and orientated downwards; dorsal branch with several very long, ventrally orientated megasetae; ventral branch with approx. two long megasetae orientated posteriorly; in dorsal view narrow, weakly undulating and tapering posteriorly, slightly diverging along their length; megasetae on dorsal branch confined to mesal surface; ventral branches orientated posteromesally, almost dilated and with long megasetae orientated posteriorly. Inferior appendages, in lateral view, with wide, truncated posteriorly; ends well before apex of sternal processes; in ventral view long, narrow basally, slightly widening from 1/3 but narrowing gradually towards rounded apex. Phallus, in lateral view slightly longer than superior appendage, almost straight; in ventral view almost equally wide along its length.

##### Additional information.

This species was referred to as “sp. 36” in Espeland and Johanson (2010a).

#### Agmina anterohamata sp. nov.

Taxon classificationAnimaliaTrichopteraEcnomidae

E35DDE3C-F904-56FA-B222-3D429698261A

http://zoobank.org/754BE67D-11D2-4063-8AEC-D5DA9F25F047

Figs 175–179

##### Diagnosis.

*Agmina anterohamata* sp. nov. is unique in the combination of having a long, narrow, and bifurcated superior appendage in lateral view together with a narrow and uniformly tapering sternal processes having a uniform dorsal curving along their length. Being armed with numerous megasetae on both dorsal and ventral branches of the superior appendage is also unique for this species.

**Figures 175–179.**
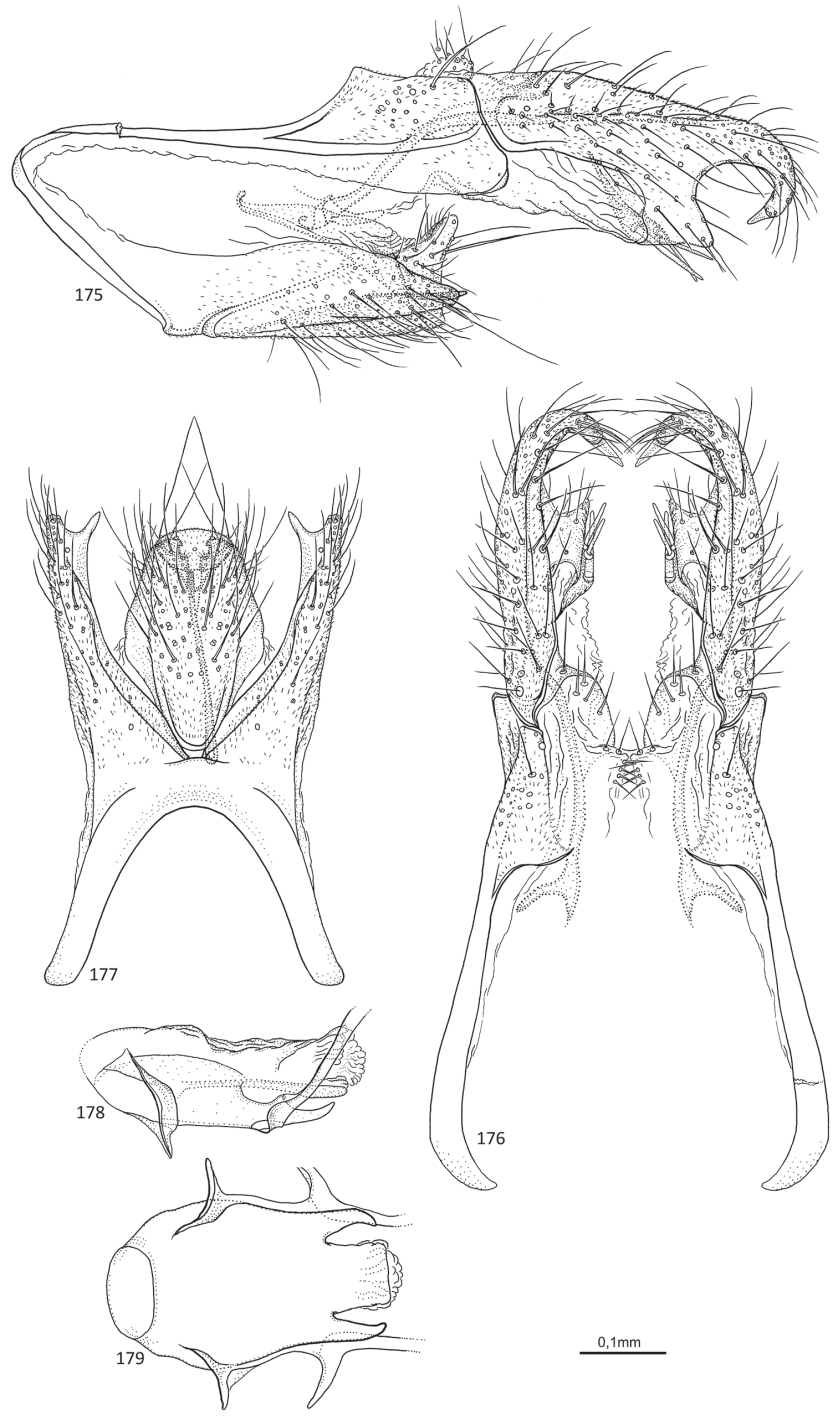
*Agmina anterohamata* sp. nov. male holotype **175** genitalia, left lateral view **176** genitalia, dorsal view **177** genitalia, ventral view **178** phallus, lateral view **179** phallus, ventral view.

##### Etymology.

*Antero* and *hamata*, from Latin, meaning anterior and hook-shaped. Referring to the forwardly curved hook at the posterior end of the superior appendages.

##### Material examined.

***Holotype***: New Caledonia – **Province Sud** • ♂; Réserve spéciale de faune de la haute Yaté, along road on southern part of Marais de la Rivière Blanche, stream draining to Marais de la Rivière Blanche, 2.25 km SW Pont Pérignon, 180 m, 6–16.xi.2003, Malaise trap, loc#010a; leg. KA Johanson; MNHN.

***Paratype***: New Caledonia – **Province Sud** • 1 ♂; Réserve spéciale de faune de la haute Yaté, along road on southern part of Marais de la Rivière Blanche, stream draining to Marais de la Rivière Blanche, 1.35 km S Pont Pérignon; 22°08.496'S, 166°42.152'E; 180 m; 6–16.xi.2003; Malaise trap; loc 009a; leg. KA Johanson; NHRS.

##### Type locality.

New Caledonia, Province Sud, Réserve spéciale de faune de la haute Yaté.

##### Measurements.

Fore wing length 4.5–4.9 mm (*N* = 2). Total length of genitalia: 0.9 mm.

##### Description.

***Genitalia***: Total length 0.9 mm. In lateral view, segment IX low, long, rounded anteriorly, apex located dorsally in genitalia; in ventral view anterior incision widely and deeply U-shaped. Sternal processes, lateral view, with slightly arrow-head shaped, narrowing from mid-length, posterior part not produced posteriorly; in ventral view, slightly narrowing till mid-length, parallel-sided from mid-length and slightly diverging along their length, each with bifurcated apex. Tergum X trapezoid with sharply pointing anteriorly and rounded pointing posteriorly; dorsal margin straight; in dorsal view, mesally separate, mesal margin indistinctly triangular. Parameres anterior part slender in lateral view, starting well below and before tergum X, short anterior part orientated posteriorly, central part orientated posterodorsally, distal part divided into branch orientated posteroventrally and dorsal branch orientated posteriorly, not exceeding superior appendages posteriorly; in dorsal view, separated along their length, bifurcated at basis, orientated posterad, few stout setae present on dorsomesal faces immediately after end of tergum X; few long megasetae present in row on plate-like mesal process; distal part completely fused with superior appendages. Superior appendages, in lateral view, long, with posterodorsal corner strongly produced into strong anteroventrally orientated hook; posteroventral corner slightly produced posterad; dorsal margin almost straight; in dorsal view slender and almost parallel-sided except narrowing towards apex; apex curving mesally and anteriorly. Inferior appendages, in lateral view, with short, finger-like dorsal branch orientated posterodorsally, posteroventral corner right-angled, without branch; in ventral view forming a nearly oval shield almost completely hiding dorsal branches; dorsal branches separated by narrow, shallow incision. Phallus, in lateral view approx. as long as inferior appendages, straight, with pair of posteriorly orientated spines situated on posteroventral margin; in ventral view wide along its length.

##### Additional information.

This species was referred to as “sp. 3” in Espeland and Johanson (2010a).

#### Agmina curvatacua sp. nov.

Taxon classificationAnimaliaTrichopteraEcnomidae

56DBC75C-F34A-5D6C-89A7-EF28E6353A31

http://zoobank.org/9DA8F862-84DA-4F36-BE63-DDD97B1046AE

Figs 180–184

##### Diagnosis.

*Agmina curvatacua* sp. nov. is unique among the *Agmina* species in that the pair of parameres form long, needle-shaped processed exceeding the posterior margin of the superior appendages; the basis of the inside of the superior appendages have the parameres with a cluster of posteromesad megasetae; and in lateral view, the sternal process tapers posteriorly and is curved ventrally before apex.

**Figures 180–184.**
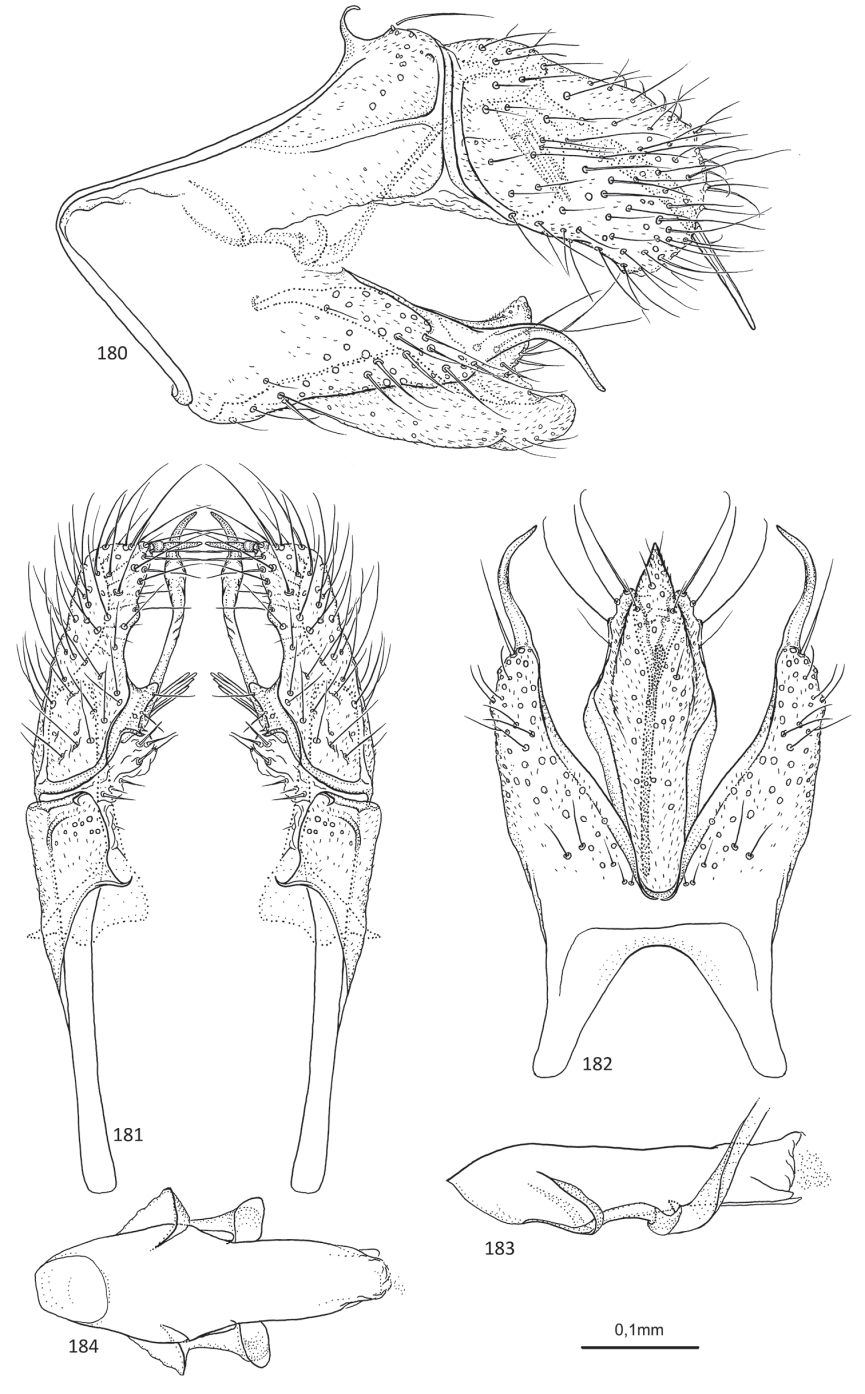
*Agmina curvatacua* sp. nov. male holotype **180** genitalia, left lateral view **181** genitalia, dorsal view **182** genitalia, ventral view **183** phallus, lateral view **184** phallus, ventral view.

##### Etymology.

*Curvata* and *acua*, from Latin, curved and needle-shaped. Referring to the curved needle-shaped posterior part of the sternal processes.

##### Material examined.

***Holotype***: New Caledonia – **Province Sud** • ♂; Dumbea River, Branche Sud; 22°08.344'S, 166°30.147'E; 42 m; 3.xi.2003; light trap; loc#006; leg. KA Johanson; MNHN.

***Paratype***: New Caledonia – **Province Sud** • 1 ♂; Dothio River, 10 m E bridge at Atè, 6.2 km WNW Thio; 21°35.288'S, 166°09.070'E; 13 m; 29.xi.2003; light trap; loc#057; leg. KA Johanson; NHRS.

##### Type locality.

New Caledonia, Province Sud, Dumbea River.

##### Measurements.

Fore wing length 2.7–3.7 mm (*N* = 2). Total length of genitalia: 0.6 mm.

##### Description.

***Genitalia***: In lateral view, segment IX evenly triangular anteriorly, apex located little below midheight of genitalia; in ventral view anterior incision widely and deeply V-shaped. Sternal processes, lateral view, with almost rectangular with straight dorsal and ventral margins, posterior margin convex; in ventral view, slightly narrowing and diverging along their length, with rounded, posteriorly directed subapical region, apex narrow and curving mesally. Tergum X in lateral view rounded triangular posteriorly, dorsal margin with dorsally orientated process approx. at mid-length; longer than high; in dorsal view, mesally separate, mesal margin almost sigmoid. Parameres anterior part slender in lateral view, starting well below and before tergum X, anterior part orientated posteriorly, central part orientated posterodorsally, distal part orientated posteroventrally and exceeding superior appendages posteriorly; in dorsal view, separated along their length, broad at basis, orientated posterad, few stout setae present on dorsomesal faces immediately after end of tergum X; long megasetae present in row on small plate-like mesal process; distal part separated from superior appendages and almost tangential before curving laterally before mesally pointing end. Superior appendages, in lateral view, almost rectangular with straight dorsal and ventral margins, posterior margin convex; in dorsal view forming parallelogram-shaped lobes with almost straight lateral and undulating mesal margins. Inferior appendages, in lateral view, with short, thick dorsal and ventral branches, dorsal branch curving slightly upward into triangular apex, ventral orientated posteriorly into rounded apex; ventral branch almost reaching as far posteriorly as sternal process; in ventral view with ventral branches forming narrowly diamond-shaped plate, almost arrow-like with rounded anterior and pointed posterior ends; dorsal branches divided by narrow, V-shaped incision above ventral branches. Phallus, in lateral view straight, approx. as long as width of genitalia; in ventral view widest immediately before mid-length, distal part almost parallel-sided.

##### Additional information.

This species was referred to as “sp. 2” in Espeland and Johanson (2010a).

### Species group 6, *bimaculata*-group

Included species in this group are: *Agmina bimaculata* Ward & Schefter, 2000, *A. recurvata* sp. nov., *A. taoensis* sp. nov., *A. triangulata* sp. nov. and *A. acula* Ward & Schefter, 2000.

The species in the *Agmina bimaculata* species group all have inferior appendages that are more or less strongly expanded posteriorly and curving dorsally in lateral view.

#### Agmina recurvata sp. nov.

Taxon classificationAnimaliaTrichopteraEcnomidae

0881387A-64CE-5309-A726-FC65501F4EE7

http://zoobank.org/BD5A7180-710A-472D-B0FA-F557B398639C

Figs 185–189

##### Diagnosis.

*Agmina recurvata* sp. nov. is unique among *Agmina* species in having left and right inferior appendages longitudinally separated along their length, each with a small mesally orientated process, and a very long posterior branch that is strongly curving postero-dorsally before meeting basis of the superior appendages. *Agmina taoensis* sp. nov. also has long inferior appendages that are curving dorsally, but these are fused into a plate, as seen in ventral view. Furthermore, it lacks the mesally orientated processes, and the posterior branch is much shorter and not so strongly curved dorsally. *Agmina cerritula* sp. nov. and *A. monstrosa* sp. nov. have similar inferior appendages in lateral view, but in these species the long processes are united into a single central process, not paired and lateral as in *A. recurvata* sp. nov.

**Figures 185–189.**
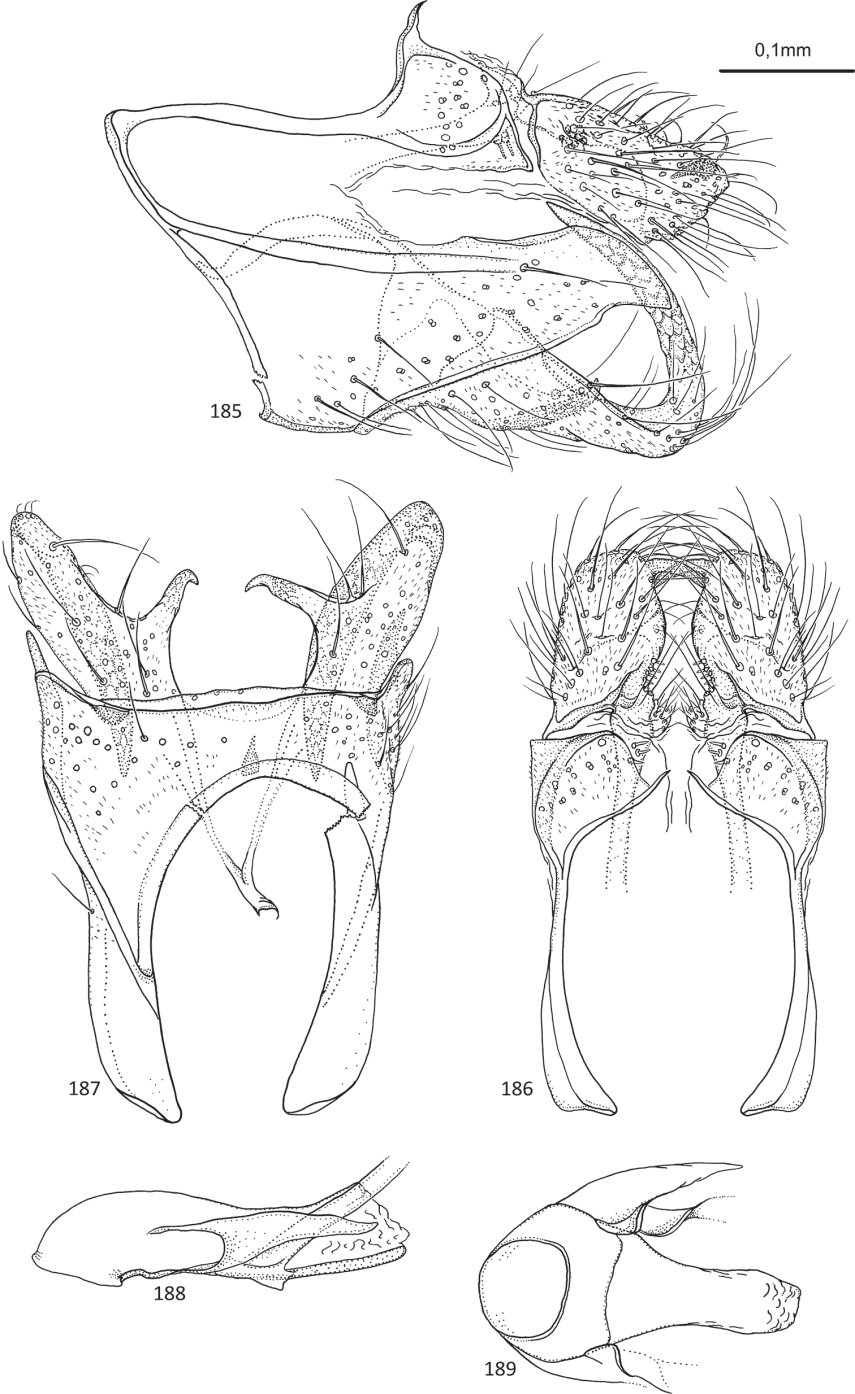
*Agmina recurvata* sp. nov. male holotype **185** genitalia, left lateral view **186** genitalia, dorsal view **187** genitalia, ventral view **188** phallus, lateral view **189** phallus, ventral view.

##### Etymology.

*Recurvata*, referring to the re-curved shape of the inferior appendages.

##### Material examined.

***Holotype***: New Caledonia – **Province Sud** • ♂; Rivière des Lacs, 1.1 km NW Lac en Huit, 4.9 km NW summit of Pic du Grand Kaori: 22°15.195'S, 166°52.178'E; 10.xii.2003; light trap; loc#078; leg. KA Johanson; MNHN.

***Paratype***: New Caledonia – **Province Sud** • 1 ♂; Rivière des Lacs, above waterfall at Chutes de Madeleine; 22°13.930'S, 166°51.633'E; 243 m; 23.xi.2003; light trap; loc#042; leg. KA Johanson; NHRS.

##### Measurements.

Fore wing length 2.1–3.6 mm (*N* = 2). Total length of genitalia: 0.5 mm.

##### Description.

***Genitalia***: In lateral view, segment IX sharply triangular anteriorly, apex located dorsally; in ventral view anterior incision widely and deeply U-shaped. Sternal processes, lateral view, with dilated posteriorly, dorsal and ventral margins almost straight; in ventral view, not clearly visible as it is hidden below inferior appendages. Tergum X in lateral view almost trapezoid, with dorsally orientated process at anterior corner; approx. as long as high; in dorsal view, mesally located close to each other but separate, anteromesal and posteromesal margins almost straight. Parameres anterior part slender, starting below and before anterior end of tergum X, almost straight till mid-height of posterior margin of tergum X, bending slightly ventrally and running into mid-part of superior appendage; in dorsal view, separated along their length, orientated posterad, equally narrow at basal half, few stout setae present on dorsal faces before end of tergum X; mesal lobe with setae present near basal part of superior appendage, fusing with superior appendage with long row of short megasetae. Superior appendages, in lateral view, dilated posteriorly, dorsal and ventral margins almost straight; in dorsal view forming oval lobes with almost straight lateral and convex mesal margins. Inferior appendages, in lateral view, with basal part narrowing posteriorly into very long and almost scale-like dorsally looped distal part; scale-like distal part orientated dorsally and lack setae; in ventral view separate along their lengths, not forming central plate, narrowly oval and divergent, posterior slander part orientated posteromesally. Phallus, in lateral view weakly sigmoid, approx. as long as inferior appendages; in ventral view vase shaped, with basal part almost double as wide as posterior part.

##### Additional information.

This species was referred to as “sp. 28” in Espeland and Johanson (2010a).

#### Agmina taoensis sp. nov.

Taxon classificationAnimaliaTrichopteraEcnomidae

E87E9BA3-2D5B-5B1F-9D78-87186D335973

http://zoobank.org/6FCF6DBE-DCA0-4566-AF39-99C50D410BAE

Figs 190–194

##### Diagnosis.

*Agmina taoensis* sp. nov. resembles *A. scopula* sp. nov. in several genitalic characters, particularly the shape of the inferior and superior appendages. *Agmina taoensis* sp. nov. is distinguished from *A. scopula* sp. nov. by the much shorter sternal processes that are apically rounded in ventral view, and the parameres lack clusters of stout mesally and posteriorly orientated setae.

**Figures 190–194.**
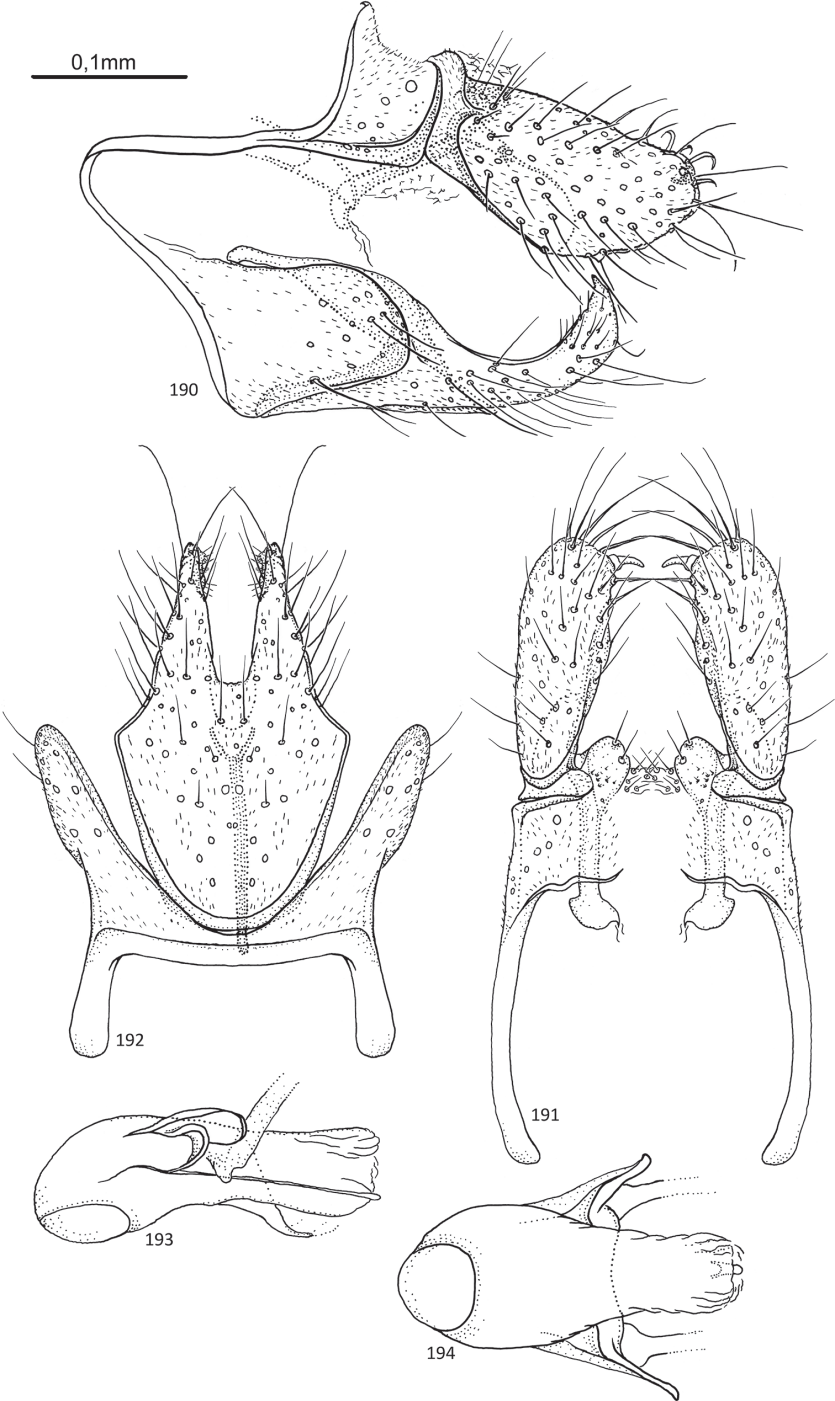
*Agmina taoensis* sp. nov. male holotype **190** genitalia, left lateral view **191** genitalia, dorsal view **192** genitalia, ventral view **193** phallus, lateral view **194** phallus, ventral view.

##### Etymology.

*Taoensis*, referring to Cascade de Tao, the type locality of the species.

##### Material examined.

***Holotype***: New Caledonia – **Province Nord** • ♂; Wé Caot Stream, draining NNE side of Mt. Panié, 0.9 km NW Cascade de Tao; 20°33.311'S, 164°48.064'E; 18.xii.2003; light trap; loc#084; leg. KA Johanson; MNHN.

##### Measurements.

Fore wing length 3.2 mm (*N* = 1). Total length of genitalia: 0.4 mm.

##### Description.

***Genitalia***: In lateral view, segment IX rounded triangular, apex located at mid-height of genitalia; in ventral view anterior incision widely and shallowly rectangularly shaped. Sternal processes, lateral view, with half circular, with almost straight dorsal margin and strongly convex ventral margin; in ventral view, almost equally wide along their length, with rounded apex, straight and diverging along their length. Tergum X in lateral view almost rectangular, with dorsally orientated process at anterior corner; slightly shorter than high; in dorsal view, mesally separate, mesal margin almost straight. Parameres slender, starting below anterior end of tergum X, almost straight till mid-height of basis of superior appendage, bending slightly ventrally before curving into posteroventral margin; in dorsal view, separated along their length, orientated posterad, equally narrow along their length, mesal lobe with setae present before superior appendage, fusing with superior appendage without long row of short megasetae. Superior appendages, in lateral view, half circular, with almost straight dorsal margin and strongly convex ventral margin; in dorsal view slightly widening along their length, each with rounded apex. Inferior appendages, in lateral view, with basal part almost rectangular, expanded into a prominent dorsally curving process being pointed apically; in ventral view almost diamond-shaped with sharply triangular lateral corners; with deep and narrow posterior incision, posterior branches orientated posteriorly and slightly diverging. Phallus, in lateral view slightly curving posteriorly at basis before sub-straight posterior main part; in ventral view vase shaped, with basal part almost double as wide as posterior part.

##### Additional information.

This species was referred to as “sp. 6” in Espeland and Johanson (2010a).

#### Agmina triangulata sp. nov.

Taxon classificationAnimaliaTrichopteraEcnomidae

36D28792-61FF-5991-98C9-C912F0D0D8FC

http://zoobank.org/3780CF2B-093B-4BA4-8267-B42EAF488911

Figs 195–200

##### Diagnosis.

*Agmina triangulata* sp. nov. resembles *A. comata* Ward, 2003 in the genitalia, particularly, in lateral view, the triangular shape of the superior appendages, and the presence of a cluster of mesally orientated setae on the median face of the superior appendages; the curving sternal processes and inferior appendages. *Agmina triangulata* sp. nov. is distinguished from *A. comata* in that the superior appendages are slightly more strongly produced posteriorly, the wider sternal process in lateral view, the much shorter dorsally curving branch of the inferior appendage in lateral view, and the more widely round shape of the inferior appendage in ventral view.

**Figures 195–200.**
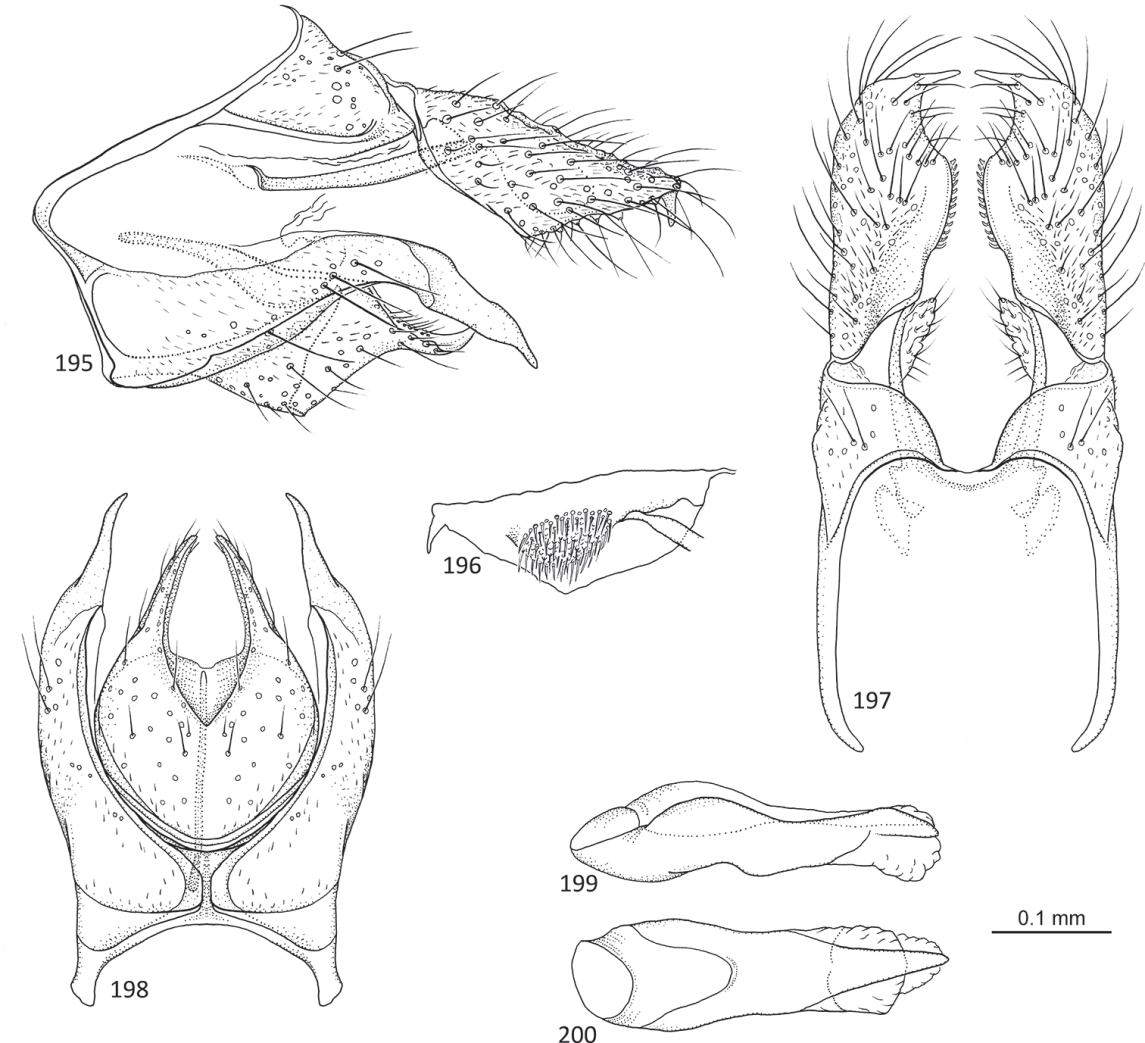
*Agmina triangulata* sp. nov. male holotype **195** genitalia, left lateral view **196** superior appendage, left lateral view of mesal surface **197** genitalia, dorsal view **198** genitalia, ventral view **199** phallus, lateral view **200** phallus, ventral view.

##### Etymology.

*Triangulata*, from Latin, meaning triangular. Referring to the shape of the superior appendages in lateral view.

##### Material examined.

***Holotype***: New Caledonia – **Province Sud** • ♂; Dumbea River, Branche Nord, 2.2 km SE summit of Mt. Piditéré; 22°07.503'S, 166°29.899'E; 25 m; 21.i.2004; light trap; loc#124; leg. KA Johanson & C Pöllabauer; MNHN.

##### Measurements.

Fore wing length 3.9 mm (*N* = 1). Total length of genitalia: 0.6 mm.

##### Description.

***Genitalia***: In lateral view, segment IX almost triangular, apex located at mid-height of genitalia; in ventral view widely oval. Sternal processes, lateral view, with sharply triangular, longer than high; in ventral view, slender, with almost uniformly concave inner margin, apex slightly curving mesally. Tergum X triangular in lateral view, slightly longer than high; in dorsal view, almost tangential mesally at narrow apices, posteriorly convex and anteriorly concave margins; Parameres slender, starting below anterior end of tergum X, almost straight till mid-height of basis of superior appendage, bending strongly ventrally before fading into posteroventral margin; in dorsal view, separated along their length, orientated posterad, slightly narrowing, with mesal lobe with small setae present before superior appendage, fusing with superior appendage and with long row of short megasetae. Superior appendages, in lateral view, sharply triangular, longer than high; in dorsal view basally narrow, expanding mesally at mid-length; apex pointing mesally; lateral margins nearly straight. Inferior appendages, in lateral view, with basal part almost rectangular, posteroventral margin parallel with posteroventral margin of sternal process, posterior corner expanded into finger-like, dorsally curved process not reaching as far posterior as apex of sternal process; in ventral view large, oval, with deep central posterior V-shaped incision; lateral processes orientated posterad and slightly mesally curving. Phallus, in lateral view sub-straight, slightly longer than superior appendage; in ventral view almost uniformly wide along its length.

##### Additional information.

This species was referred to as “sp. 14” in Espeland and Johanson (2010a).

### Species group 7, *bleuensis*-group

Included species in this group are: *Agmina bleuensis* sp. nov., *A. hastata* Ward & Schefter, 2000, *A. hamata* Ward & Schefter, 2000, *A. hirta* Ward & Schefter, 2000, *A. touhoensis* sp. nov., *A. wardi* sp. nov., *A. vuegi* Ward & Schefter, 2000, and *A. kara* Ward & Schefter, 2000.

The species in the *Agmina bleuensis* species group are characteristic in having very long and slender sternal processes that are curving ventrally towards the apex in lateral view.

#### Agmina bleuensis sp. nov.

Taxon classificationAnimaliaTrichopteraEcnomidae

754927FB-C41E-5302-9605-8AF5AEA7621D

http://zoobank.org/BB45EA8B-C546-4A4F-91C5-299D585CF8B2

Figs 201–205

##### Diagnosis.

*Agmina bleuensis* sp. nov. resembles most other species in the species group, particularly *A. touhoensis* sp. nov. and *A. hamata* due to the wide separation of the dorsal and ventral branches of the inferior appendages in lateral view. *Agmina bleuensis* sp. nov. is distinguished from the two other species by the thicker ventral branch of inferior appendages. In addition, the sternal processes of *A. bleuensis* sp. nov. is much shorter than in *A. touhoensis* sp. nov. and *A. hamata*.

**Figures 201–205.**
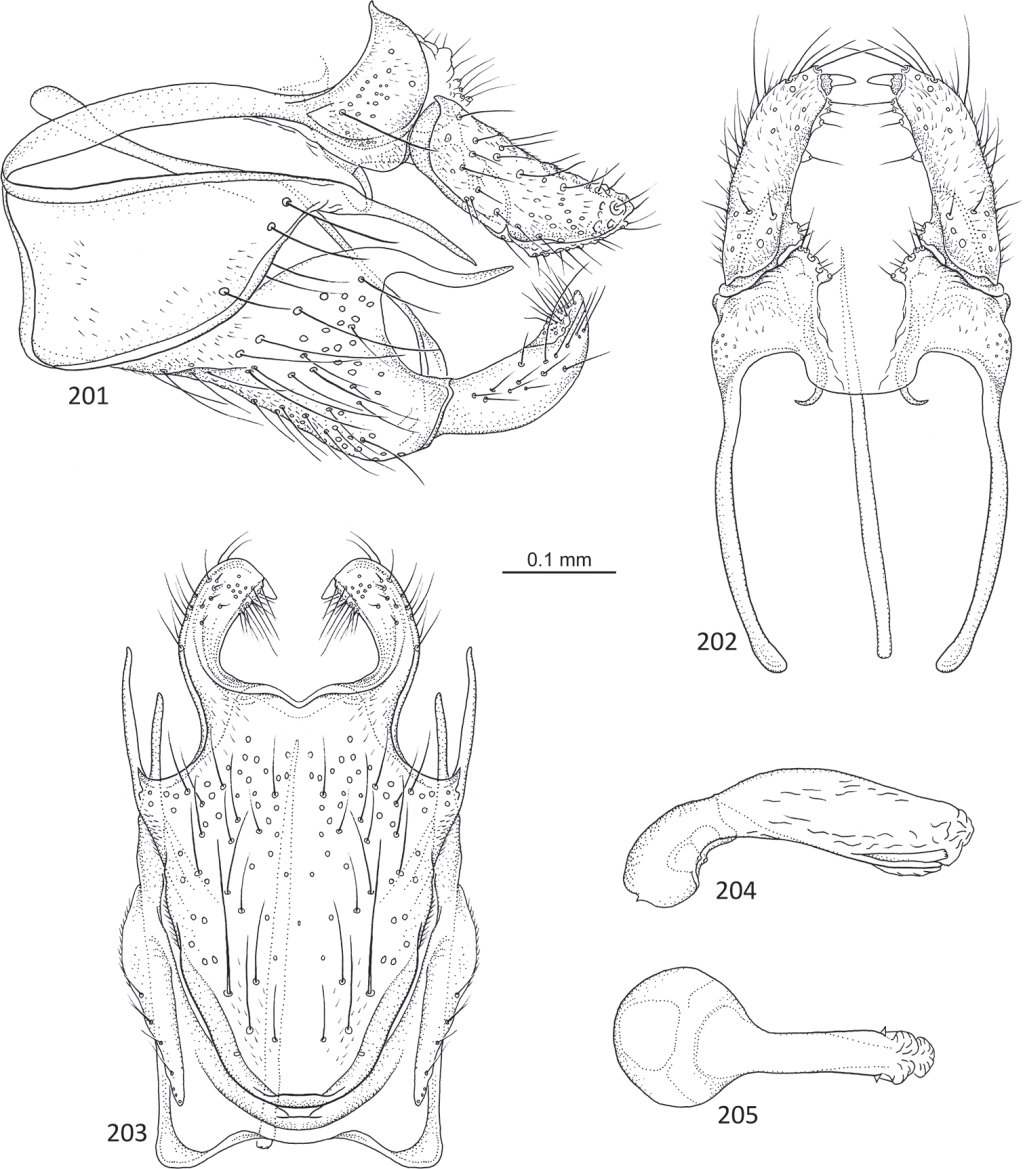
*Agmina bleuensis* sp. nov. male holotype **201** genitalia, left lateral view **202** genitalia, dorsal view **203** genitalia, ventral view **204** phallus, lateral view **205** phallus, ventral view.

##### Etymology.

*bleuensis*, derived from Rivière Bleue, the type locality of the species.

##### Material examined.

***Holotype***: New Caledonia – **Province Sud** • ♂; Rivière Bleue, 2.7 km SSW of summit of Montagne Bleue; 22°05.705'S, 166°38.225'E; loc 139a (loc 4-2001); Malaise trap; 13–16.xi.2001; leg. KA Johanson, T Pape & B Viklund; MNHN.

***Paratypes***: New Caledonia – **Province Sud** • 2 ♂; Parc territorial de la Rivière Bleue, Riviere Bleue, 22°05.826'S, 166°38.293'E, loc#127, light trap 6–7.x.2006; leg. KA Johanson & M Espeland; NHRS; • 1 ♂; stream crossing Nouméa-Yaté road immediately W of turnoff to Rivière Bleue Reserve; 22°10.191'S, 166°44.474'E; 162 m; 5.x.2006; light trap; loc#040b; leg. KA Johanson & M Espeland; NHRS; • 1 ♂; stream draining to Rivière des Pirogues 850 m E summit of Mont Imbaah, 5.5 km E Lucky Creek in Plum; 22°16.837'S, 166°42.195'E; 31 m; 1.xii.2003; light trap, loc#060; leg. KA Johanson; NHRS.

##### Measurements.

Fore wing length 3.6–4.9 mm (*N* = 5). Total length of genitalia: 0.5 mm.

##### Description.

***Genitalia***: In lateral view, segment IX almost constituting of sternal process only; anterior apex located at mid-height of genitalia; in ventral view anteriorly without incision. Sternal processes, lateral view, with small, slender, approx. twice as long as high, with slightly concave dorsal margin, ventral margin convex; in ventral view, slender, orientated posteriorly along their length; apex pointed. Tergum X sharply tapering dorsally, higher than long; in dorsal view, mesally separate, each with irregular mesal margin. Parameres weakly developed, with small, narrow, ventral branch before superior appendages, continues into dorsal part of superior appendage before looping downwards into ventral margin of superior appendage; in dorsal view, slender, curving mesally and ending at mid-length of superior appendages; each with wart-like structure at mid-length with few short setae. Superior appendages, in lateral view, small, slender, approx. twice as long as high, with slightly concave dorsal margin, ventral margin convex; in dorsal view parallel-sided and slightly curved mesally along their length, rounded apex with mesal tooth. Inferior appendages, in lateral view, with broad basis and two long posteriorly orientated branches; dorsal branch slightly curving upwards, ending near mid-length of superior appendage, apex needle shaped; ventral branch uniformly thick, curving slightly outwards along its length; in ventral view with shield-shaped basis; dorsal branches located laterally of ventral branches, almost straight and orientated posterad along their length; ventral branches broad and curving mesally along their length, apex on each side well separated mesally; apex widely triangular; setae present on most parts. Phallus, in lateral view almost as long as segment IX, curving posterad at 1/4; in ventral view vase-shaped, with wide rounded basis and narrow posterior two-thirds.

##### Additional information.

This species was referred to as “sp. 49” in Espeland and Johanson (2010a).

#### Agmina touhoensis sp. nov.

Taxon classificationAnimaliaTrichopteraEcnomidae

1D8BEACA-520E-5DC2-840E-400FAB5C220E

http://zoobank.org/F8EBA7E0-7450-49C0-85B4-96EA74EA5B15

Figs 206–211

##### Diagnosis.

*Agmina touhoensis* sp. nov. resembles most other species in the species group, particularly *A. bleuensis* sp. nov. and *A. hamata* due to the wide separation of the dorsal and ventral branches of the inferior appendages in lateral view. *Agmina touhoensis* sp. nov. is distinguished from *A. bleuensis* sp. nov. by the much longer sternal processes, and a dorsal branch of the inferior appendages that are almost as long as the ventral branches. It is distinguished from *A. hamata* by the wider superior appendages in lateral view, and the dorsal branch of inferior appendages that is almost straight instead of curving ventrally.

**Figures 206–211.**
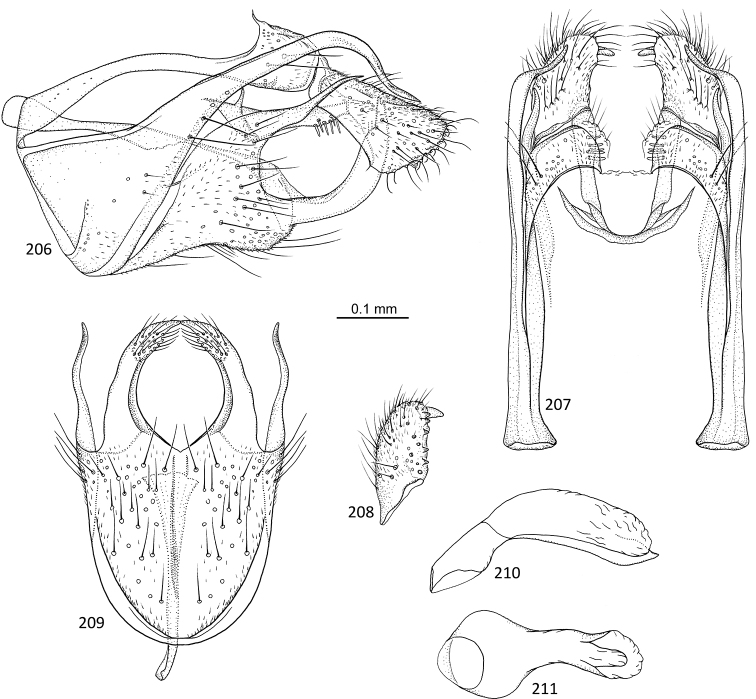
*Agmina touhoensis* sp. nov. male holotype **206** genitalia, left lateral view **207** right superior appendage, ventral face in dorsal view **208** genitalia, dorsal view **209** genitalia, ventral view **210** phallus, lateral view **211** phallus, ventral view.

##### Etymology.

*Touhoensis*, derived from Touho, the type locality of the species.

##### Material examined.

***Holotype***: New Caledonia – **Province Nord** • ♂; Ponandou Tiôgé River at Kögi, 3.9 km SSW Touho; 20°49.043'S, 165°13.551'E; 25 m; 26.xii.2003; light trap; loc#100; leg. KA Johanson; MNHN.

##### Measurements.

Fore wing length 4.6 mm (*N* = 1). Total length of genitalia: 0.6 mm.

##### Description.

***Genitalia***: In lateral view, segment IX almost constituting of sternal process only; anterior apex located immediately below mid-height of genitalia; in ventral view anteriorly without incision. In lateral view, sternal processes with small, almost straight dorsal margin, ventral margin pointing into triangular; in ventral view, slender, orientated posteriorly before strongly curving mesally before apex. Tergum X almost rectangular, with pointed anterodorsal corner; in dorsal view, mesally well separate, each forming narrow plate curving inwards, with slightly convex inner margin. Parameres robust, tubular, starting below tergum X and ending at basal 1/3 of superior appendages, hidden behind upper branch of inferior appendages; in dorsal view, originating from transverse bow-like basis; orientated posteriorly, almost parallel, row of short megasetae present immediately after mid-length. Superior appendages, in lateral view, small, with almost straight dorsal margin, ventral margin pointing into triangular; in dorsal view bean-shaped, orientated posterad along their length, except slightly mesally orientated, rounded corners. Inferior appendages, in lateral view, with broad basis and two long posteriorly orientated branches; dorsal branch slightly undulating, ending near basis of superior appendages, apex needle shaped; ventral branch uniformly thick, curving outwards along its length; in ventral view with shield-shaped basis; dorsal branches located laterally of ventral branches, undulating and orientated posterad along their length; ventral branches broad and curving mesally along their length and almost tangential apically; apex narrowly triangular; setae confined to distal two-thirds. Phallus, in lateral view approx. half as long as segment IX, curving downwards at mid-length; in ventral view vase-shaped, with wide rounded basis and narrow posterior half.

##### Additional information.

This species was referred to as “sp. 23” in Espeland and Johanson (2010a).

#### Agmina wardi sp. nov.

Taxon classificationAnimaliaTrichopteraEcnomidae

D7E440BE-797A-52A8-A785-42DC624889D8

http://zoobank.org/D7D76454-B06E-4848-AAEA-246BE56E3DB6

Figs 212–216

##### Diagnosis.

The long and dorsally curving inferior appendages of *A. wardi* sp. nov. makes it resembling *A. recurvata* sp. nov., *A. cerritula* sp. nov. and *A. monstrosa* sp. nov. It is distinguished from the two latter by having paired processes of inferior appendages instead of a simple process; and from *A. recurvata* sp. nov. by the posteriorly narrowing sternal processes that end in a thick, straight apical megaseta that is orientated posteroventrally.

**Figures 212–216.**
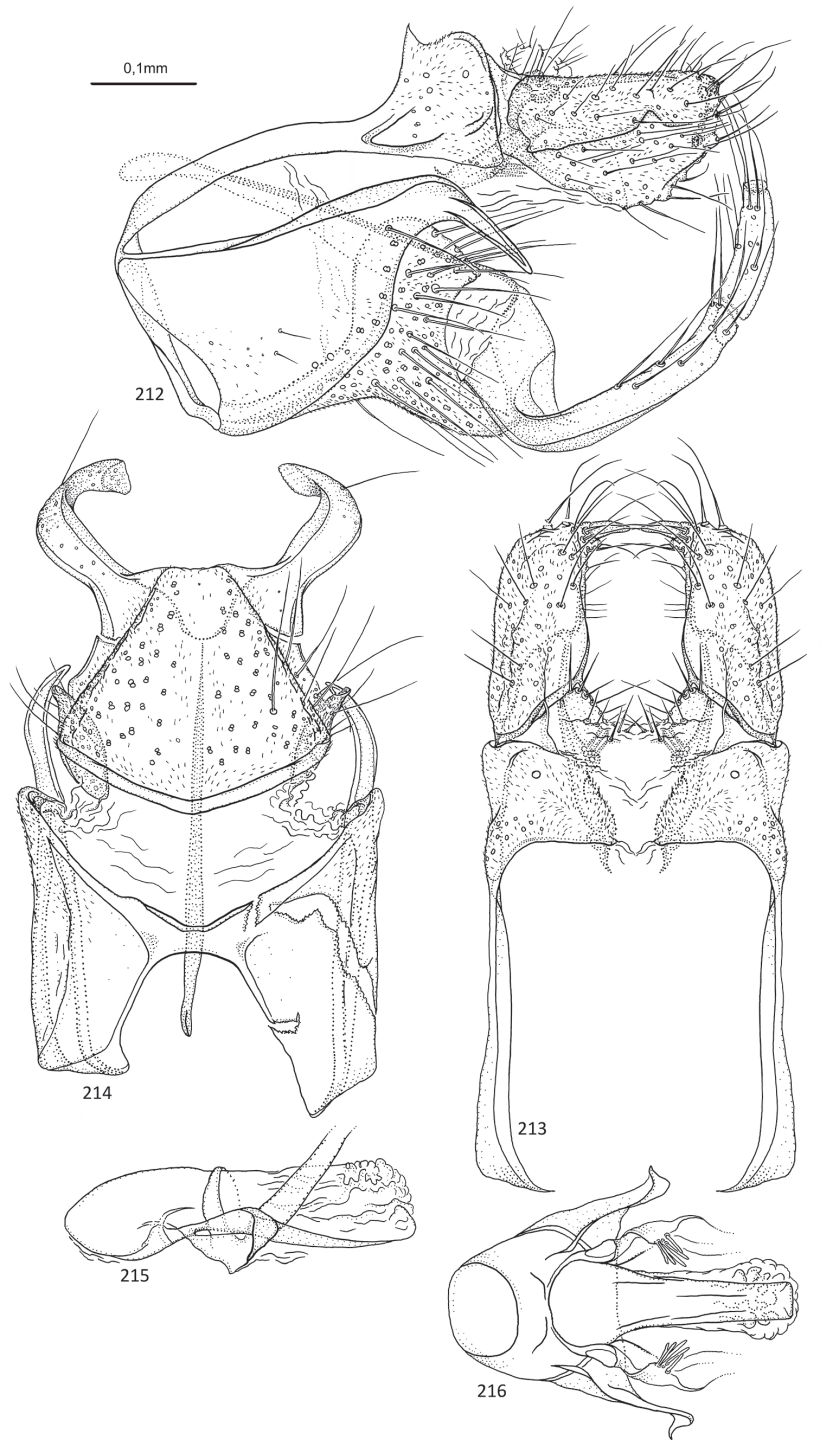
*Agmina wardi* sp. nov. male holotype **212** genitalia, left lateral view **213** genitalia, dorsal view **214** genitalia, ventral view **215** phallus, lateral view **216** phallus, ventral view.

##### Etymology.

*Wardi*, named for John B. Ward, for his contribution to understanding the diversity of New Caledonian *Agmina* diversity.

##### Material examined.

***Holotype***: New Caledonia – **Province Sud** • ♂; Rivière des Lacs, 1.1 km NW Lac en Huit, 4.9 km NW summit of Pic du Grand Kaori; 22°15.195'S, 166°52.178'E; 10.xii.2003; light trap; loc#078; leg. KA Johanson; MNHN.

***Paratypes***: New Caledonia – **Province Sud** • 1 ♂; Creek Pernod, 7 m downstream bridge at Route du Carénage on Lac Yaté-Prony road; 22°10.862'S, 166°50.565'E; 162 m; 10.xii.2003; light trap; loc#076; leg. KA Johanson; NHRS; • 1 ♂; Rivière des Lacs, above waterfall at Chutes de Madeleine; 22°13.930'S, 166°51.633'E; 243 m; 23.xi.2003; light trap; loc#042; leg. KA Johanson; NHRS.

##### Measurements.

Fore wing length 3.8–4.0 mm (*N* = 3). Total length of genitalia: 0.6 mm.

##### Description.

***Genitalia***: In lateral view, segment IX almost constituting of sternal process only; anterior apex located immediately below mid-height of genitalia; in ventral view anteriorly with widely U-shaped incision. Sternal processes, lateral view, with large, half circular with almost straight dorsal margin, without spines; in ventral view, slender and curving mesally from mid-length. Tergum X almost rectangular with posterodorsal rounded corner and anterodorsal pointed corner, in lateral view higher than long; in dorsal view, mesally situated closely but separate, each forming trapezoid lobes with straight inner margin. Parameres starting from lower anterior part of tergum X, narrow, bifurcating into dorsal and ventral branches basally in superior appendage; in dorsal view, forming pair of narrowing rays, each with small megasetae in short row located midway on mesal margin. Superior appendages, in lateral view, large, half circular with almost straight dorsal margin, without spines; in dorsal view uniformly wide along their length, parallelogram-shaped; apex strongly inwardly pointed. Inferior appendages, in lateral view, with broad basal part covered by setae separated from L-shaped mid-part that is smooth basally and with setae distally; apparently with a distal joint as long as height of tergum X; in ventral view with widely triangular basal part and truncate posterior corner; central part orientated laterally before looping mesally at mid-length; apex almost rounded. Phallus, in lateral view slightly shorter than length of segment IX, straight; in ventral view vase-shaped, with wide basis and narrow posterior half.

##### Additional information.

This species was referred to as “sp. 39” in Espeland and Johanson (2010a).

### Species group 8, *padi*-group

Included species in this group are: *Agmina padi* Ward & Schefter, 2000, *A. parallela* sp. nov., *A. christinae* sp. nov., *A. rhara* Ward & Schefter, 2000, *A. parie* Ward & Schefter, 2000, *A. brevis* sp. nov., *A. ninguana* sp. nov., *A. diriwi* Ward & Schefter, 2000, *A. scopula* sp. nov., and *A. comata* Ward, 2003. No particular morphological characteristics are observed for this species group.

#### Agmina parallela sp. nov.

Taxon classificationAnimaliaTrichopteraEcnomidae

9E53CECB-0673-5CB4-9FBF-239FBA01BB82

http://zoobank.org/581632F3-2640-4D13-ACD9-41B8676FFE24

Figs 217–221

##### Diagnosis.

*Agmina parallela* sp. nov. unique among *Agmina* species in the superior appendage that in lateral view is almost as large as segment IX and segment X combined, and is rounded club-shaped ventrally. In addition, the inferior appendages form a long ventral plate that is almost parallel-sided along its length.

**Figures 217–221.**
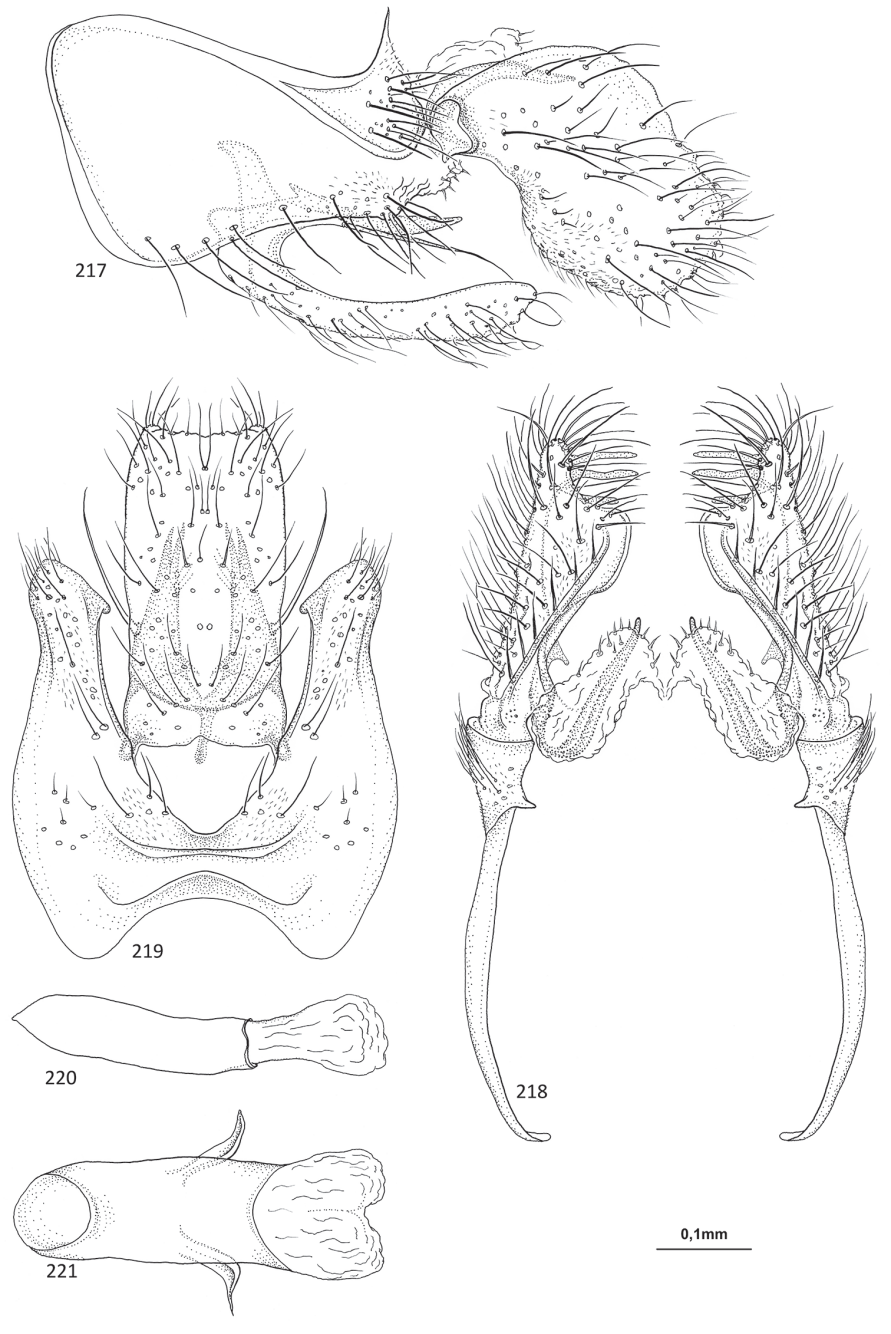
*Agmina parallela* sp. nov. male holotype **217** genitalia, left lateral view **218** genitalia, dorsal view **219** genitalia, ventral view **220** phallus, lateral view **221** phallus, ventral view.

##### Etymology.

*Parallela*, derived from parallel, referring to the inferior appendages having almost parallel-sided lateral margins in ventral view.

##### Material examined.

***Holotype***: New Caledonia – **Province Nord** • ♂; Ponandou Tiôgé River at Kögi, 3.9 km SSW Touho; 20°49.043'S, 165°13.551'E; 25 m; 26.xii.2003; light trap; loc#100; leg. KA Johanson; MNHN.

##### Measurements.

Fore wing length 4.5 mm (*N* = 1). Total length of genitalia: 0.7 mm.

##### Description.

***Genitalia***: In lateral view, segment IX widely rounded anteriorly, almost trapezoid, apex located dorsally; in ventral view anteriorly with widely and shallow U-shaped incision. Sternal processes, lateral view, with very large, downwardly club-shaped, posterior and anterior margins almost parallel, apex widely rounded; in ventral view, absent. Tergum X deeply concave dorsally, posteriorly expanded dorsad into pointed triangular, in lateral view approx. as long as high; in dorsal view, forming small plates widely separated mesally. Parameres dorsally membranous, ventrally forming strongly sclerotised spines reaching to half-length of superior appendages; in lateral view slightly curving posteriorly; in dorsal view, separate and re-curved as basis, needle-shaped, almost straight after basis and pointing mesally. Superior appendages, in lateral view, very large, downwardly club-shaped, posterior and anterior margins almost parallel, apex widely rounded; in dorsal view narrow at basis, widening into mesal plates at mid-length, small mesally orientated tooth present at basis; row of long apical megasetae situated on inner surface and orientated mesally. Inferior appendages, in lateral view, with posterad orientated long dorsal branch with pointed apex; dorsal branch widely separated from ventral branch; ventral branch running parallel with dorsal branch, approx. double the width of dorsal branch and gently curving dorsally along its length; apex narrowly rounded; in ventral view rectangular plate-like ventral branch hiding dorsal branches, dorsal branches orientated posteriorly, each uniformly narrowing into acute apex. Phallus, in lateral view as long as segment IX, slender and slightly curving upwards; in ventral view equally wide along its length, double as wide as high.

##### Additional information.

This species was referred to as “sp. 51” in Espeland and Johanson (2010a).

#### Agmina christinae sp. nov.

Taxon classificationAnimaliaTrichopteraEcnomidae

8684E4AF-3534-58DB-AF1B-1AD6DA62E57D

http://zoobank.org/92AF7BBE-4187-439F-BE5B-EAC1AFF9321D

Figs 222–226

##### Diagnosis.

*Agmina christinae* sp. nov. has inferior appendages that form a ventral plate with a broad basis and a long central process, which resemble the plate of *A. piscaria* sp. nov., but the plate of *A. christinae* sp. nov. is broader than that of *A. piscaria* sp. nov. It also resembles *A. rhara* by the presence of megasetae dorsally of the basis of the superior appendages. *Agmina christinae* sp. nov. is easily distinguished from *A. rhara* by having two branched inferior appendages in lateral view instead of three-branched inferior appendages, and the superior appendages of *A. christinae* sp. nov. are oval while those of *A. rhara* are long and narrow.

**Figures 222–226.**
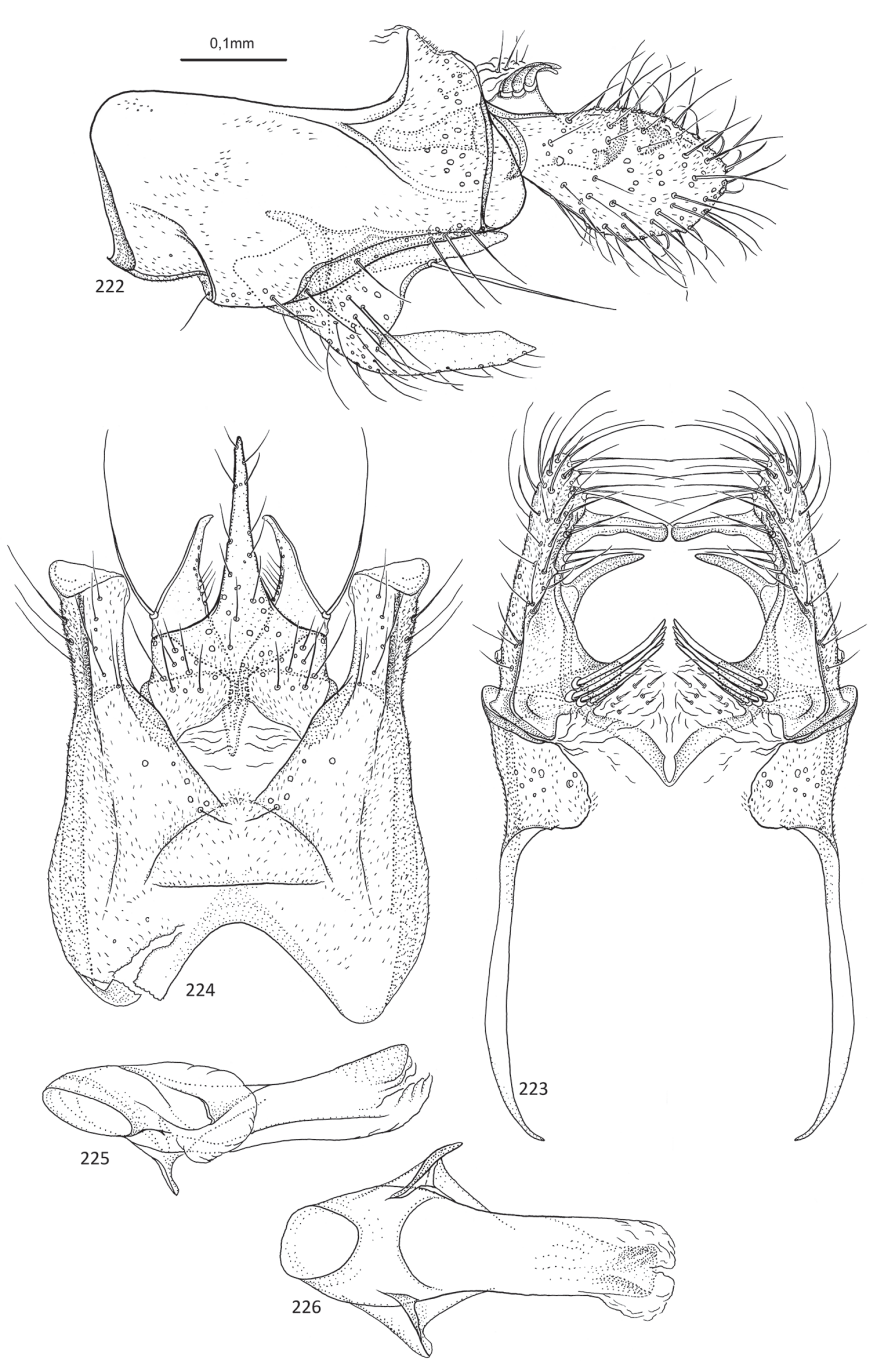
*Agmina christinae* sp. nov. male holotype **222** genitalia, left lateral view **223** genitalia, dorsal view **224** genitalia, ventral view **225** phallus, lateral view **226** phallus, ventral view.

##### Etymology.

*Christinae*, named for one of the collectors of this species, Dr. Christine Pöllabauer.

##### Material examined.

***Holotype***: New Caledonia – **Province Nord** • ♂; stream in Creek de Bambou, 5 m N road RT7 Ouégoa-Koumac; 20°27.863'S, 164°19.784'E; 58 m; 19.xii.2003; Malaise trap; loc#087; leg. KA Johanson; MNHN.

***Paratypes***: New Caledonia – **Province Nord** • 1 ♂; same data as holotype, except NHRS; • 1 ♂; stream in Creek de Bambou, ca. 20 m upstream bridge on road RT7 Ouégoa-Koumac; 20°27.715'S, 164°20.978'E; 105 m; 19.xii.2003; Malaise trap; loc#086; leg. KA Johanson; NHRS.

##### Measurements.

Fore wing length 3.8–4.5 mm (*N* = 3). Total length of genitalia: 0.8 mm.

##### Description.

***Genitalia***: In lateral view, segment IX almost truncate anteriorly, apex located dorsally; in ventral view with anterior incision widely and deeply V-shaped. Sternal processes, lateral view, with large, oval, somewhat stalked basally, almost symmetrical dorsal and ventral sides; in ventral view, wide basally, narrowing to midway, almost parallel-sided along distal half; orientated posteriorly. Tergum X strongly concave before pointing dorsally, in lateral view slightly higher than long; in dorsal view, mesally separate, each forming short lobes with rounded margins. Parameres starting from anterior part of tergum X, posteriorly with small membranous dorsal part and sclerotised ventral part, ventral part with cluster of megasetae visible above base of superior appendages on mid-length of inner margin, a hook-like curved spine situated apically on each paramere and situated on the inside of superior appendages; in dorsal view, anterior parts fused into a V-shaped suture, basal megasetae almost meeting mesally, distal hooks curving inwards along their length, situated immediately anteriorly of large mesal processes. Superior appendages, in lateral view, large, oval, somewhat stalked basally, almost symmetrical dorsal and ventral sides; in dorsal view slightly widening, straight, and converging along their length, with long, mesal process before apex. Inferior appendages, in lateral view, with two posteriorly orientated almost parallel branches in lateral view, dorsal branch with a long and thick posteriorly orientated seta on ventral margin, apex acute; ventral branch as long as dorsal branch and with pointed apex; in ventral view, ventral branch with almost square basal half and single-rayed, narrow, tapering distal half; dorsal branches forming a pair of narrow triangular processes curving mesally at apex. Phallus, in lateral view slightly shorter than segment IX, slender and slightly curving upwards; in ventral view with triangular lateral processes on anterior half, distal half approx. equally wide.

##### Additional information.

This species was referred to as “sp. 1” in Espeland and Johanson (2010a).

#### Agmina brevis sp. nov.

Taxon classificationAnimaliaTrichopteraEcnomidae

0EF5958C-DA23-55E2-9488-C9160529E3DF

http://zoobank.org/BC71FC71-C448-41DC-A883-E417B6F94755

Figs 227–231

##### Diagnosis.

*Agmina brevis* sp. nov. is unique in having inferior appendages which, in lateral view, have equally short dorsal and ventral branches separated by a U-shaped cleft, and the superior appendage is approx. as high as long in lateral view.

**Figures 227–231.**
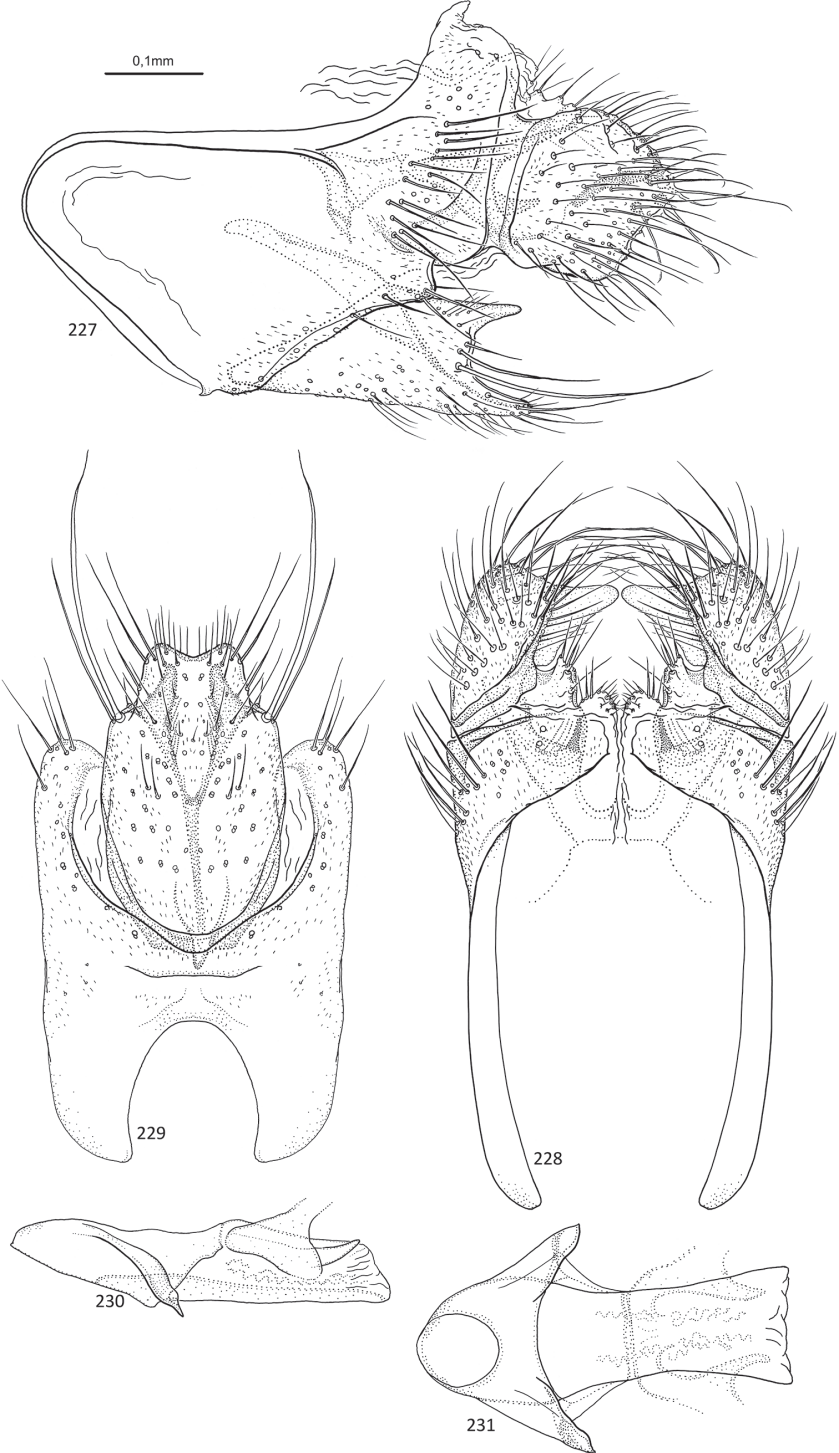
*Agmina brevis* sp. nov. male holotype **227** genitalia, left lateral view **228** genitalia, dorsal view **229** genitalia, ventral view **230** phallus, lateral view **231** phallus, ventral view.

##### Etymology.

*Brevis*, from Latin, meaning short. Referring to the shape of the superior appendage in lateral view, being shorter than high.

##### Material examined.

***Holotype***: New Caledonia – **Province Nord** • ♂; Réserve spéciale de faune de l’Aoupinié, ca 25 km S Poindimié; 21°09.775'S, 165°19.017'E; loc#148 (18-2001); Malaise trap; 24–28.xi.2001; leg. KA Johanson, T Pape & B Viklund; MNHN.

***Paratype***: New Caledonia – **Province Nord** • 1 ♂; Réserve spéciale de faune de l’Aoupinié, ca 25 km S Poindimié; 21°09.369'S, 165°19.209'E; loc#149 (19-2001); Malaise trap; 24–28.xi.2001; leg. KA Johanson, T Pape & B Viklund; NHRS.

##### Measurements.

Fore wing length 4.8–5.0 mm (*N* = 2). Total length of genitalia: 0.7 mm.

##### Description.

***Genitalia***: In lateral view, segment IX widely rounded anteriorly, apex located dorsally; in ventral view with anterior incision narrowly U-shaped, almost semi-oval. Sternal processes, lateral view, with shorter than high, slightly trapezoid; in ventral view, not clearly set off from rest of segment IX. Tergum X higher than long, convex dorsally and with small tapering dorsal process, in dorsal view, separate but almost tangential mesally, with rounded inner margins. Parameres robust, starting inside basis of tergum X, dividing before superior appendages into long, narrow and pointed dorsal branch and short, pointing ventral branch, both branches ending before mid-length of superior appendage; in dorsal view, strongly fused and indistinguishable from superior appendage. Superior appendages, in lateral view, shorter than high, slightly trapezoid; in dorsal view almost parallelogram-shaped, with near parallel lateral and mesal margins; strong triangular mesally orientated tooth situated near apex. Inferior appendages, in lateral view, with almost parallel dorsal and ventral margins, posteriorly divided into short narrow triangular dorsal and almost equally short similarly shaped ventral branches widely separated by U-shaped cleft; in ventral view large, longer than wide, with convex lateral margins, narrowing at three-fourth its length; apex with central cleft; dorsal branches visible through the ventral branch, forming pair of well-separated triangular processes. Phallus, in lateral view, slightly shorter than segment IX, uniformly slender along its length, straight; in ventral view with triangular lateral processes at basal 1/3; distal two-thirds with slightly concave lateral margins.

##### Additional information.

This species was referred to as “sp. 33” in Espeland and Johanson (2010a).

#### Agmina ninguana sp. nov.

Taxon classificationAnimaliaTrichopteraEcnomidae

37BED74C-DB90-5620-839B-54FA0090256A

http://zoobank.org/3D819D7E-ECEA-4F70-9E1B-E4BFE1059C50

Figs 232–236

##### Diagnosis.

*Agmina ninguana* sp. nov. is very similar to both *A. scopula* sp. nov. and *A. hexacantha* in having very broad inferior appendages in lateral view, each with a dorsally curved ventral branch. They also have a group of mesally orientated megasetae in the central part of the mesal face of the superior appendages. *Agmina ninguana* sp. nov. is distinguished from *A. hexacantha* by the broader and shorter sternal processes in lateral view and from *A. scopula* sp. nov. by the straighter dorsal margin of the superior appendages and much narrower sternal process.

**Figures 232–236.**
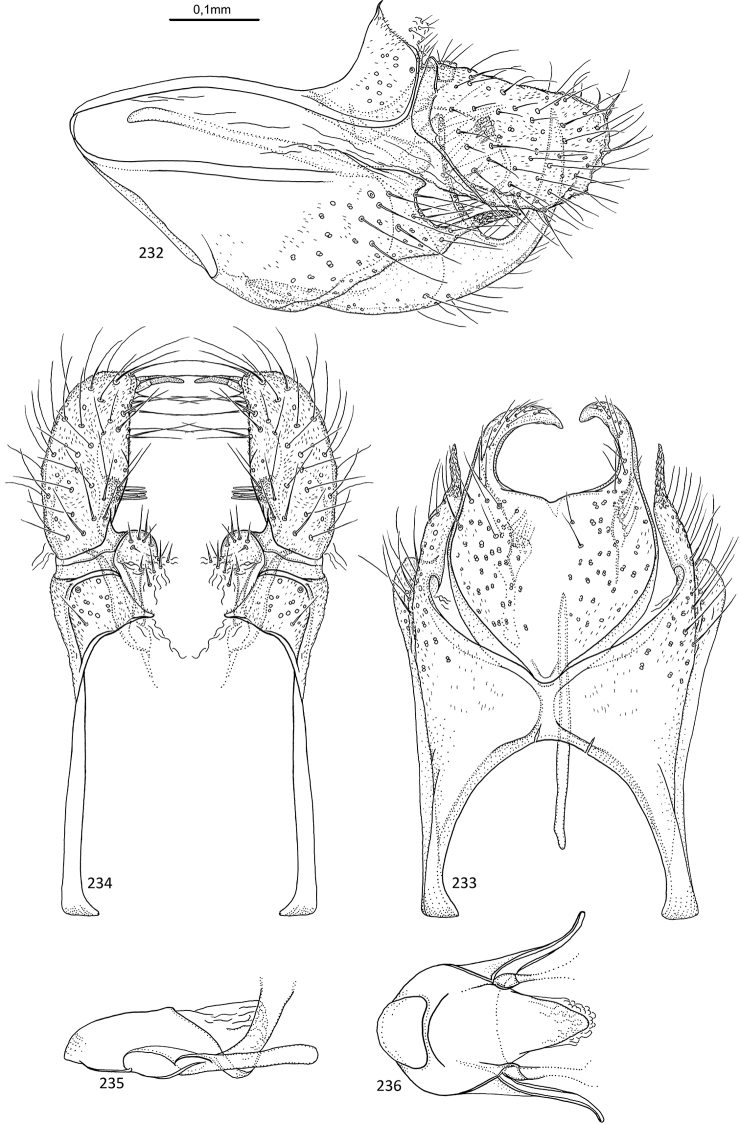
*Agmina ninguana* sp. nov. male holotype **232** genitalia, left lateral view **233** genitalia, dorsal view **234** genitalia, ventral view **235** phallus, lateral view **236** phallus, ventral view.

##### Etymology.

*Ninguana*, derived from Mont Ningua, the type locality of the species.

##### Material examined.

***Holotype***: New Caledonia – **Province Sud** • ♂; western part of Mt. Ningua, Kwé Néco Stream, 3.9 km W summit of Mt. Ningua, on Boulo-Thio Road, ca. 50 m upstream road; 21°44.359'S, 166°06.009'E; 117 m; 20.xi.2003–12.xii.2003; Malaise trap; loc#035; leg. KA Johanson; MNHN.

***Paratypes***: New Caledonia – **Province Sud** • 1 ♂; Dothio River, 10 m E bridge at Atè, 6.2 km WNW Thio; 21°35.288'S, 166°09.070'E; 13 m; 29.xi.2003; light trap; loc#057; leg. KA Johanson; NHRS; • 1 ♂; W slope Mt. Ningua, Kwé Néco Stream, at Camp Jacob, 3.7 km WNW summit of Mt. Ningua, on Boulo-Thio Road, ca. 50 m upstream road; 21°43.613'S, 166°06.567'E; 150 m; 29.xi-12.xii.2003; Malaise trap; loc#054; leg. KA Johanson; NHRS.

##### Measurements.

Fore wing length 3.4–4.5 mm (*N* = 3). Total length of genitalia: 0.6 mm.

##### Description.

***Genitalia***: In lateral view, segment IX narrowly rounded anteriorly, apex located dorsally; in ventral view anterior incision widely U-shaped. Sternal processes, lateral view, with large, almost half-spherical with straight dorsal margin; in ventral view, slender, basal part slightly orientated mesally, bent posteriorly at mid-length. Tergum X sharply pointing dorsally, higher than long in lateral view; in dorsal view, mesally separate, each forming short lobes with almost straight inner margins. Parameres weakly developed, starting at basis of tergum X; in lateral view, bending upwards and meeting superior appendage basally at dorsal margin; in dorsal view, almost invisible, with group of stout, strictly mesally orientated megasetae. Superior appendages, in lateral view, large, almost half-spherical with straight dorsal margin; in dorsal view, narrowly parallel-sided along their length, smoothly convex lateral margins; apex with stout, finger-like spine pointing mesally. Inferior appendages, in lateral view, with ventral margin uniformly convex along its length; dorsal branch orientated strictly upwards, sharply triangular, with very long posterior margin ending in well separated ventral branch; ventral branch curving upwards along its length, with acute apex; in ventral view, with large central plate being approx. as long as wide, with convex lateral margins and triangular anteriorly; posteriorly with pair of long, widely separated processes curving mesally along their length. Phallus, in lateral view, much shorter than segment IX, straight; in ventral view, triangular with rounded anterior margin and pointed posteriorly.

##### Additional information.

This species was referred to as “sp. 25” in Espeland and Johanson (2010a).

#### Agmina scopula sp. nov.

Taxon classificationAnimaliaTrichopteraEcnomidae

D3752517-FE96-53D0-9467-962E73A278FB

http://zoobank.org/C380D645-B166-473B-9893-646C9BF5676B

Figs 237–241

##### Diagnosis.

In lateral view of the genitalia, *A. scopula* sp. nov. is similar to both *A. ninguana* sp. nov. and *A. hexacantha* in the very broad inferior appendages, each with a dorsally curved ventral branch, and presence of a group of mesally orientated megasetae in the central part of the mesal face of the superior appendages. *Agmina scopula* sp. nov. is distinguished from both *A. ninguana* sp. nov. and *A. hexacantha* by the clearly broader sternal processes in lateral view, more strongly curve of the ventral branch of the inferior appendages, and the more convex margin of the superior appendage.

**Figures 237–241.**
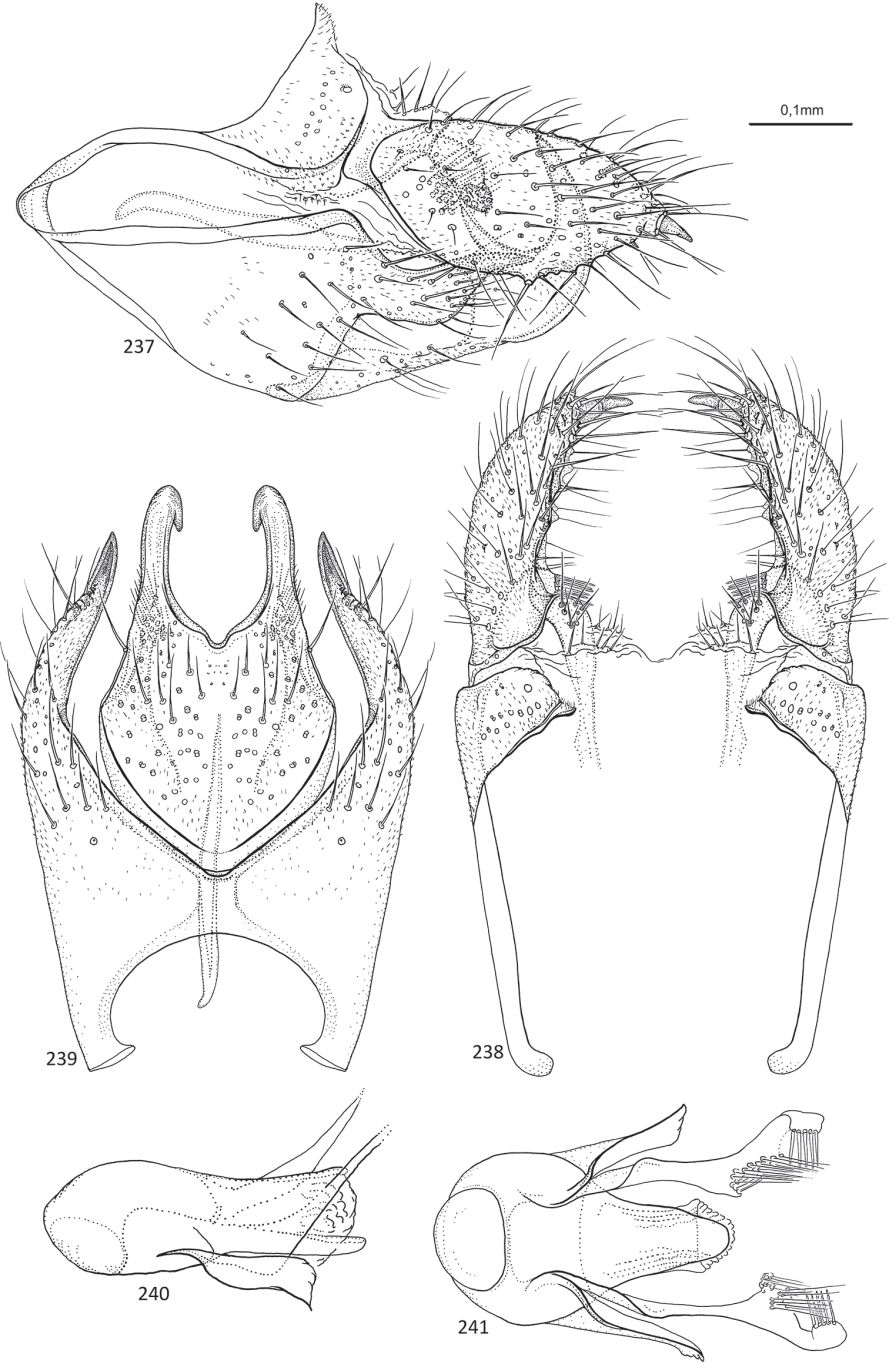
*Agmina scopula* sp. nov. male holotype **237** genitalia, left lateral view **238** genitalia, dorsal view **239** genitalia, ventral view **240** phallus, lateral view **241** phallus, ventral view.

##### Etymology.

From Latin *scopula* (noun, feminine), meaning brush. Referring to the mesally orientated setae at the inner margin of each paramere.

##### Material examined.

***Holotype***: New Caledonia – **Province Sud** • ♂; Sarramea, stream Xwé Wya, ca 0.9 km NE Hotel Evasion 130; 21°38.081'S, 165°51.735'E; loc#131a (10-2001); light trap; 19.xi.2001; leg. KA Johanson, T Pape & B Viklund; MNHN.

##### Measurements.

Fore wing length 5.0 mm (*N* = 1). Total length of genitalia: 0.7 mm.

##### Description.

***Genitalia***: In lateral view, segment IX narrowly rounded anteriorly, apex located at mid-height of genitalia; in ventral view, anterior incision widely U-shaped, with slightly mesally orientated anterior corners. Sternal processes, lateral view, with large, oval, slightly deeper convex ventrally than dorsally; mesal processes exceeds apex; in ventral view, basally wide, tapering and curving inwards along their length. Tergum X sharply pointing dorsally, higher than long in lateral view; in dorsal view, mesally separate, each forming short lobes with mesally pointing anterior corner. Parameres weakly developed, starting at lower basis of tergum X; in lateral view, bending upwards and with downwardly curving rounded apex; in dorsal view, almost invisible, with group of stout, antero-mesally orientated megasetae. Superior appendages, in lateral view, large, oval, slightly deeper convex ventrally than dorsally; mesal processes exceeds apex; in dorsal view, narrowly parallel-sided and curving inwards along their length, smoothly convex lateral margins; apex with stout, finger-like spine pointing mesally. Inferior appendages, in lateral view, with basal half of ventral margin almost straight, distal half uniformly convex; dorsal branch orientated strictly upwards, widely triangular, with concave posterior margin ending in well separated ventral branch; ventral branch wide at base, tapering and curving upwards along its length, with acute apex; in ventral view, with large central plate being approx. as long as wide, with angled lateral margins and triangular anteriorly; posteriorly with pair of long, widely separated processes orientated posteriorly along their length. Phallus, in lateral view approx. as long as segment IX, straight; in ventral view, triangular with rounded anterior margin and pointed posteriorly.

##### Additional information.

This species was referred to as “sp. 9” in Espeland and Johanson (2010a).

### New species records

The following records are herewith recorded as new. The material is deposited at NHRS.

***Agmina acula* Ward, 2003**

New Caledonia – **Province Sud** • 1 ♂; Dumbea River, Branche Nord, 2.2 km SE summit of Mt. Piditéré; 22°07.503'S, 166°29.899'E; 25 m; 21.i.2004; light trap; loc#124a; leg. KA Johanson & C Pöllabauer; • 1 ♂; Dumbea River, Branche Sud; 22°08.344'S, 166°30.147'E; 42 m; 3.xi.2003; light trap; loc#006; leg. KA Johanson.

***Agmina artarima* Ward & Schefter, 2000**

New Caledonia – **Province Sud** • 1 ♂; Couvelée River at Haute Couvelée, 2.8 km SV summit of Mt. Piditéré, 3.5 km (air) NNE Dumbéa; 22°07.488'S, 166°28.034'E; 27 m; 28.xi.2003; light trap; loc#051; leg. KA Johanson; • 1 ♂; on road between Nouméa and Yaté, 1.0 km NW Pont des Japonais; 22°11.421'S, 166°42.840'E; 114 m; 22.xi-4.xii.2003; Malaisetrap; loc#038; leg. KA Johanson; • 1 ♂; Dumbea River, Branche Sud; 22°08.344'S, 166°30.147'E; 42 m; 3.xi.2003; light trap; loc#006; leg. KA Johanson.

***Agmina berada* Ward & Schefter, 2000**

New Caledonia – **Province Sud** • 1 ♂; Plaine des Gaïacs, Rivière Rouge, 14.2 km NW summit of Mt. Rouge, 50 m upstream road RT1 Nouméa-Koné; 20°31.573'S, 164°46.690'E; 23 m; 2.i.2004; light trap; loc#104; leg. KA Johanson.

***Agmina bimaculata* Ward & Schefter, 2000**

New Caledonia – **Province Sud** • 1 ♂; Couvelée River at Haute Couvelée, 2.8 km SV summit of Mt. Piditéré, 3.5 km (air) NNE Dumbéa; 22°07.488'S, 166°28.034'E; 27 m; 28.xi.2003; light trap; loc#051; leg. KA Johanson; • 1 ♂; Dumbea River, Branche Sud; 22°08.344'S, 166°30.147'E; 42 m; 3.xi.2003; light trap; loc#006; leg. KA Johanson; • 1 ♂; Tontouta River, 4.8 km WSW summit of Mt. Vulcain, 21°55.258'S, 166°19.895'E, 41 m, 15.xii.2003, light trap, loc#083; leg. KA Johanson.

***Agmina cheirella* Ward, 2003**

New Caledonia – **Province Sud** • 1 ♂; Dumbea River, Branche Sud; 22°08.344'S, 166°30.147'E; 42 m; 3.xi.2003; light trap; loc#006; leg. KA Johanson;

***Agmina comata* Ward, 2003**

New Caledonia – **Province Sud** • 1 ♂; Mt. Dzumac, source stream of Ouinne River, downstream crosspoint to mountain track; 22°02.330'S, 166°28.605'E; 796 m; 3.xii.2003; light trap; loc#062; leg. KA Johanson.

***Agmina diriwi* Ward & Schefter, 2000**

New Caledonia – **Province Sud** • 2 ♂; Monts Kwa Ne Mwa, along Nouméa-Yaté road, 2.0 km E Pic Mouirange, 20 m upstream road; 22°12.356'S, 166°40.798'E; 220 m; 15–16.i.2004; light trap; loc#120; leg. KA Johanson; • 1 ♂; stream crossing way to Sanatorium 2.3 km E St. Laurent, ca. 30 m downstream bridge; 22°04.484'S, 166°19.900'E; 15.xi.2003; light trap; loc#028; leg. KA Johanson;

***Agmina hamata* Ward & Schefter, 2000**

New Caledonia – **Province Sud** • 1 ♂; Plateau de Dogny; 846 m; 21°37.000'S, 165°52.500'E; loc#145 (15-2001); Malaise trap; 18–21.xi.2001; leg. KA Johanson, T Pape & B Viklund; • 1 ♂; Haute Yaté fauna reserve, 1760 m S bridge Pont Perignon, 50 m upstream bridge over stream; 22.14954S, 166.701211E; 180 m; 14.xii.2003–13.i.2004; Malaise trap; loc#081; leg. KA Johanson.

***Agmina hastata* Ward & Schefter, 2000**

New Caledonia – **Province Sud** • 1 ♂; Dumbea River, Branche Nord, 2.2 km SE summit of Mt. Piditéré; 22°07.503'S, 166°29.899'E; 25 m; 21.i.2004; light trap; loc#124a; leg. KA Johanson & C Pöllabauer; • 2 ♂; stream crossing Nouméa-Yaté road immediately W of turnoff to Rivière Bleue Reserve; 22°10.191'S, 166°44.474'E; 162 m; 22.xi-4.xii.2003; Malaise trap; loc#040; leg. KA Johanson; • 1 ♂; Dumbea River, Branche Sud; 22°08.344'S, 166°30.147'E; 42 m; 3.xi.2003; light trap; loc#006; leg. KA Johanson.

***Agmina hirta* Ward & Schefter, 2000**

New Caledonia – **Province Sud** • 1 ♂; Col d’Amieu, Fo Waau Stream, at Pont Ouaou; 21°35.559'S, 165°48.311'E; 317 m; 10.i.2004; light trap; loc#116; leg. KA Johanson.

***Agmina jepiva* Ward & Schefter, 2000**

New Caledonia – **Province Nord** • 1 ♂; stream in Creek de Bambou, 5 m N road RT7 Ouégoa-Koumac; 20°27.863'S, 164°19.784'E; 58 m; 19.xii.2003; Malaise trap; loc#087; leg. KA Johanson.

***Agmina joycei* Ward & Schefter, 2000**

New Caledonia – **Province Sud** • 1 ♂; Dumbea River, Branche Nord, 2.2 km SE summit of Mt. Piditéré; 22°07.503'S, 166°29.899'E; 25 m; 21.i.2004; light trap; loc#124a; leg. KA Johanson & C Pöllabauer; • 1 ♂; Dumbea River, Branche Sud; 22°08.344'S, 166°30.147'E; 42 m; 3.xi.2003; light trap; loc#006; leg. KA Johanson;

***Agmina kapiwa* Ward & Schefter, 2000**

New Caledonia – **Province Sud** • 2 ♂; Réserve spéciale de faune de la haute Yaté, along road on southern part of Marais de la Rivière Blanche, stream draining to Marais de la Rivière Blanche, 2.25 km SW Pont Pérignon, 180 m, 6–16.xi.2003, Malaise trap, loc#010a; leg. KA Johanson; • 1 ♂; stream crossing Nouméa-Yaté road, 1.5 km S Yaté Dam, ca. 200 m upstream the road; 22°09.931'S, 166°52.535'E; 197 m; 22.xi-17.xii.2003; Malaise trap; loc#041; leg. KA Johanson.

***Agmina kara* Ward & Schefter, 2000**

New Caledonia – **Province Sud** • 1 ♂; Col d’Amieu, Fo Waau Stream, at Pont Ouaou; 21°35.559'S, 165°48.311'E; 317 m; 10.i.2004; light trap; loc#116; leg. KA Johanson; • 1 ♂; Tamoa River, 700 m S road RT1 between Nouméa and La Foa; 22°04.518'S, 166°16.592'E; 19.xi.2003; light trap; loc#033; leg. KA Johanson; • 1 ♂; Rivière des Lacs, above waterfall at Chutes de Madeleine; 22°13.930'S, 166°51.633'E; 243 m; 23.xi.2003; light trap; loc#042; leg. KA Johanson; • 1 ♂; Couvelée River at Haute Couvelée, 2.8 km SV summit of Mt. Piditéré, 3.5 km (air) NNE Dumbéa; 22°07.488'S, 166°28.034'E; 27 m; 28.xi.2003; light trap; loc#051; leg. KA Johanson.

***Agmina mariae* Ward & Schefter, 2000**

New Caledonia – **Province Sud** • 1 ♂; Mt. Dzumac, source stream of Ouinne River, near crossing point to mountain track; 22°02.439'S, 166°28.646'E; 805 m; 18.xi-4.xii.2003; Malaise trap; loc#029; leg. KA Johanson; • 1 ♂; stream crossing way to Sanatorium 2.3 km E St. Laurent, ca. 30 m downstream bridge; 22°04.484'S, 166°19.900'E; 15.xi.2003; light trap; loc#028; leg. KA Johanson; • 1 ♂; Haute Yaté fauna reserve, 1760 m S bridge Pont Perignon, 50 m upstream bridge over stream; 22.14954S, 166.701211E; 180 m; 14.xii.2003–13.i.2004; Malaise trap; loc#081; leg. KA Johanson; **Province Nord** • 1 ♂; Ponandou Tiôgé River at Kögi, 3.9 km SSW Touho; 20°49.043'S, 165°13.551'E; 25 m; 26.xii.2003; light trap; loc#100; leg. KA Johanson; • 1 ♂; Mt. Panié, stream at camp; 20.58139S, 164.76444E; 1310 m; 9.xii.2003; Malaise trap; loc#074; leg. KA Johanson.

***Agmina nodosa* Ward, 2003**

New Caledonia – **Province Sud** • 1 ♂; Haute Yaté fauna reserve, 1760 m S bridge Pont Perignon, 50 m upstream bridge over stream; 22.14954S, 166.701211E; 180 m; 14.xii.2003–13.i.2004; Malaise trap; loc#081; leg. KA Johanson; • 1 ♂; Rivière des Lacs, 1.1 km NW Lac en Huit, 4.9 km NW summit of Pic du Grand Kaori; loc#078; 22°15.195'S, 166°52.178'E; 10.xii.2003; light trap; leg. KA Johanson; • 1 ♂; Creek Pernod, 7 m downstream bridge at Route du Carénage on Lac Yaté-Prony road; 22°10.862'S, 166°50.565'E; 162 m; 10.xii.2003; light trap; loc#076; leg. KA Johanson; • 1 ♂; Mt. Dzumac, source stream of Ouinne River, downstream crossing point to mountain track; 22°01.997'S, 166°28.486'E; 795 m; over ca. 30 m waterfall; 18.xi-4.xii.2003; Malaise trap; loc#031; leg. KA Johanson.

***Agmina panda* Ward & Schefter, 2000**

New Caledonia – **Province Sud** • 1 ♂; Plateau de Dogny, source Dogny River, ca. 1.4 km SE summit of Platou, ca. 20 m upstream waterfall; 21.62054S, 165.88503E; 912 m; 25.xi-16.xii.2003; Malaise trap; loc#049; leg. KA Johanson; **Province Nord** • 1 ♂; Aoupinié Mt., Réserve spéciale de faune de l’Aoupinié, spring to side stream to Öröpömwati River; 21°09.032'S, 165°19.179'E; 441 m; 6–27.xii.2003; Malaise trap; loc#065; leg. KA Johanson.

***Agmina padi* Ward & Schefter, 2000**

New Caledonia – **Province Sud** • 1 ♂; Parc territorial de la Rivière Bleue, Riviere Bleue; 22°05.826'S, 166°38.293'E; loc#127; light trap; 6–7.x.2006; leg. KA Johanson & M Espeland; • 1 ♂; lower part Rivière des Pirogues, 800 m WNW summit of Mont Imbaah, 4.7 km E Lucky Creek in Plum; 22°18.559'S, 166°41.227'E; 1.3 m; 1.xii.2003; light trap; loc#059; leg. KA Johanson; • 1 ♂; Rivière des Lacs, at camp-site ca. 200 m from Route du Carénage between Lac Yaté and Prony, 800 m N summit of ancient mine Anne Madeleine; 22°13.295'S, 166°50.888'E; 223 m; 10.xii.2003; light trap; loc#077; leg. KA Johanson; • 1 ♂; Tontouta River, 4.8 km WSW summit of Mt. Vulcain; 21°55.258'S, 166°19.895'E; 41 m; 15.xii.2003; light trap; loc#083; leg. KA Johanson; • 1 ♂; Dumbea River, Branche Sud; 22°08.344'S, 166°30.147'E; 42 m; 3.xi.2003; light trap; loc#006; leg. KA Johanson.

***Agmina parie* Ward & Schefter, 2000**

New Caledonia – **Province Sud** • 1 ♂; Monts Kwa Ne Mwa, along Nouméa-Yaté road, 2.0 km E Pic Mouirange, 20 m upstream road; 22°12.356'S, 166°40.798'E; 220 m; 15–16.i.2004; light trap; loc#120; leg. KA Johanson; **Province Nord** • 1 ♂; Amoa River, ca 12 km W Poindimié; 20°58.092'S, 165°11.804'E; loc 150 (20-2001); light trap; 25–26.xi.2001; leg. KA Johanson, T Pape & B Viklund; • 1 ♂; Mt. Panié, Riv. Padyéém, 400 m, 20°34.122'S, 164°48.147'E, 22–28.xi.2001; Malaise trap; loc#146 (16-2001); leg. KA Johanson, T Pape & B Viklund.

***Agmina rhara* Ward & Schefter, 2000**

New Caledonia – **Province Nord** • 1 ♂; Ponandou Tiôgé River at Kögi, 3.9 km SSW Touho; 20°49.043'S, 165°13.551'E; 25 m; 26.xii.2003; light trap; loc#100; leg. KA Johanson; • 1 ♂; Réserve spéciale de faune de l’Aoupinié, ca 25 km S Poindimié, 21°08.940'S, 165°19.409'E, loc#147a (17-2001), Malaise trap; 24–28.xi.2001; leg. KA Johanson, T Pape & B Viklund.

***Agmina urugi* Ward & Schefter, 2000**

New Caledonia – **Province Sud** • 1 ♂; St. Vincent, Bongou Stream, at bridge on road to Tribu de Bangou, 700 m N RT1 Nouméa-Tontoutu road; 22°03.477'S, 166°15.718'E; 26.xi.2003; light trap; loc#050; leg. KA Johanson.

***Agmina vuegi* Ward & Schefter, 2000**

New Caledonia – **Province Sud** • 1 ♂; Xwé Pemöu Stream, 300 m N bridge over Dathio River at Atè, 6.2 km WNW of Thio; 21.58835S, 166.15117E; 13 m; 29.xi.2003; light trap; loc#056; leg. KA Johanson; • 1 ♂; 2.8 km ENE Bopope, at site where Rivière Kövé Tamè enters Rivière Oua Mendiou, 100 m S RPN2 Koné-Poindimié; 20°54.455'S, 165°06.300'E; 78 m; 14.i.2003; light trap; loc#119; leg. KA Johanson.

### Key to described *Agmina* species, males

In the key, *A. touhoensis* sp. nov. is keyed out twice due to variation in relative length between the inferior appendages and sternal processes due to viewing angle of the inferior appendages.

**Table:** 

1	Segment IX with each sternal process exceeding the inferior appendages posteriorly	**2**
–	Segment IX with each sternal process reaching approximately as far posteriorly or less far posteriorly than inferior appendages	**31**
2	In ventral view, plate of inferior appendage undivided longitudinally or without posteriorly orientated lateral processes (Figs 12, 49)	**3**
–	In ventral view, plate of inferior appendage with incision on posterior margin (Figs 127, 168) or with posteriorly orientated lateral processes (Fig. 43)	**12**
3	In ventral view, plate of inferior appendage with prominent apical process much narrower than rest of plate (Fig. 61)	**4**
–	In ventral view, plate of inferior appendage without prominent apical process (Figs 12, 143, 173)	**7**
4	In lateral view, plate of inferior appendage with pair of long posterodorsal branches (Figs 89, 94); paraprocts without cluster of megasetae	**5**
–	In lateral view, plate of inferior appendage without posterodorsal branches (Figs 46, 64); paraprocts with posterior cluster of megasetae (Figs 48, 60)	**6**
5	In lateral view, superior appendages slightly shorter that tergum X (Fig. 69); inferior appendages with posterodorsal branches narrow and slightly curving dorsally (Fig. 69)	***A. rectangulata* sp. nov.**
–	In lateral view, superior appendages slightly longer that tergum X (Fig. 94); inferior appendages with posterodorsal branches broad and almost straight (Fig. 94)	***A. chela* sp. nov.**
6	Paraprocts very long and exceeding superior appendages posteriorly (Figs 46, 49); sternal process slender and almost straight (Fig. 46)	***A. longispina* sp. nov.**
–	Paraprocts much shorter, not exceeding superior appendages posteriorly (Fig. 69); sternal process broad and boomerang-shaped (Fig. 58)	***A. longicordata* sp. nov.**
7	In lateral view, superior appendages divided into a dorsal and a ventral branch (Figs 140, 171)	**8**
–	In lateral view, superior appendages undivided (Figs 10, 69)	**9**
8	Superior appendages with very long megasetae on branches (Fig. 171); sternal process curving dorsally along its length (Fig. 171)	***A. mana* sp. nov.**
–	Superior appendages without long megasetae on branches (Fig. 140); sternal process curving ventrally along its length (Fig. 140)	***A. falx* sp. nov.**
9	In lateral view, superior appendages almost 2 times longer than tergum X; sternal process slightly curving dorsally along its length	***A. nodosa* Ward, 2003**
–	In lateral view, superior appendages as long as or shorter than tergum X; sternal process slightly curving ventrally or almost straight along its length	**10**
10	In ventral view, plate of inferior appendages strongly narrowing along its length (Fig. 12); in lateral view, sternal process bifurcated (Fig. 10)	***A. rocheta* sp. nov.**
–	In ventral view, plate of inferior appendages with lateral margins almost parallel-sided or diverging along its length (as in Figs 49, 71); in lateral view, sternal process not bifurcated (Fig. 69)	**11**
11	In lateral view, sternal process straight and thin; in ventral view, plate of inferior appendages with lateral margins almost parallel-sided along its length (as in Fig. 49)	***A. jepiva* Ward & Schefter, 2000**
–	In lateral view, sternal process curving ventrally and thick; in ventral view, plate of inferior appendages with lateral margins diverging posteriorly (Fig. 71)	***A. semicampanula* sp. nov.**
12	In ventral view, plate of inferior appendages with long lateral processes (as in Fig. 28)	**13**
–	In ventral view, plate of inferior appendages with short lateral processes (Figs 38, 43, 148)	**14**
13	In lateral view, inferior appendages divided into a dorsal and a ventral branch (Fig. 206)	***A. touhoensis* sp. nov.**
–	In lateral view, inferior appendages forming a single branch	**53**
14	In lateral view, superior appendages longer than tergum X (Figs 105, 115, 166)	**15**
–	In lateral view, superior appendages as long as or shorter than tergum X (Figs 64, 135)	**23**
15	In lateral view, sternal process pointing posteroventrally (Fig. 195)	**16**
–	In lateral view, sternal process pointing posterodorsally (Fig. 195) or posteriorly (Figs 105, 115)	**18**
16	In ventral view, plate of inferior appendages generally oval with deep posterior incision (Fig. 198); superior appendages with dense area of small setae on mesal face (Fig. 196)	***A. triangulata* sp. nov.**
–	In ventral view, plate of inferior appendages generally narrow with shallow or deep posterior incision (Figs 122, 127); superior appendages without dense area of small setae on mesal face	**17**
17	In lateral view, sternal processes almost parallel-sided along their length (Fig. 120)	***A. dathioensis* sp. nov.**
–	In lateral view, sternal processes strongly narrowing at mid-length (Fig. 125)	***A. rougensis* sp. nov.**
18	In lateral view, superior appendages bifurcating (Fig. 105); sternal processes almost straight (Fig. 105)	**19**
–	In lateral view, superior appendages not bifurcating (Figs 115, 166); sternal processes slightly sigmoid (Fig. 115, 166)	**21**
19	Superior appendages each with mesad part approx. as long as posterad part (Fig. 106)	***A. amplexa* sp. nov.**
–	Superior appendages each without long mesad part (Ward and Schefter 2000: fig. 46)	**20**
20	Large sclerotised paraproct present between sternal processes and superior appendages reaches as far posteriorly as superior appendages (Ward and Schefter 2000: fig. 41)	***A. kapiwa* Ward & Schefter, 2000**
–	Paraproct poorly developed, not seen between sternal processes and superior appendages (Ward and Schefter 2000: fig. 45)	***A. artarima* Ward & Schefter, 2000**
21	In lateral view, sternal processes broad, almost parallel-sided along their length, with truncate apex (Fig. 115)	***A. rostrata* sp. nov.**
–	In ventral view, sternal processes narrowing along their length, apex pointed (Fig. 166)	**22**
22	In lateral view, superior appendages slender, almost parallel-sided along their length (Fig. 166); paraproct with row of posteroventrally orientated megasetae (Fig. 166)	***A. dognyensis* sp. nov.**
–	In lateral view, superior appendages high, triangular (Fig. 115); paraproct without megasetae (Fig. 115)	***A. joycei* Ward & Schefter, 2000**
23	In lateral view, sternal process sigmoid, apically curving dorsally (Ward and Schefter 2000: fig. 39); superior appendages with long, slender ventrally orientated process (Ward and Schefter 2000: fig. 39)	***A. urugi* Ward & Schefter, 2000**
–	In lateral view, sternal process almost straight or weakly curving (Figs 36, 64, 74); superior appendages without long, slender ventrally orientated process (Fig. 41), but a hook-like spine can be present (Fig. 130)	**24**
24	In ventral view, plate of inferior appendages with posterad lateral processes broadly separated by U-shaped incision, with central process between lateral processes filling all or part of the space (Figs 38, 43, 55, 66)	**25**
–	In ventral view, plate of inferior appendages with posterad lateral processes narrowly separated by V-shaped incision, without central process between lateral processes (Figs 76, 132, 137, 148)	**28**
25	In lateral view, tergum X approx. as long as superior appendages (Figs 41, 64)	**26**
–	In lateral view, tergum X longer than superior appendages (Figs 36, 52)	**27**
26	In lateral view, sternal processes almost straight along their length (Fig. 41); inferior appendages hidden behind sternal processes (Fig. 41); in ventral view, plate of inferior appendages with narrow, spine-like central posterad process (Fig. 43)	***A. digitata* sp. nov.**
–	In lateral view, sternal processes slightly bent ventrally at mid-length (Fig. 64); inferior appendages visible below sternal processes (Fig. 64); in ventral view, plate of inferior appendages with very wide central posterad process (Fig. 66)	***A. campanula* sp. nov.**
27	Superior appendages apically with large, claw-like spine approx. as long as rest of superior appendages (Fig. 53)	***A. magnahamata* sp. nov.**
–	Superior appendages apically with small, slightly curved spine approx. 1/5 as long as rest of superior appendages (Fig. 37)	***A. circulata* sp. nov.**
28	In lateral view, tergum X approx. as long as superior appendages (Figs 130, 146)	**29**
–	In lateral view, tergum X longer than superior appendages (Figs 74, 135)	**30**
29	Superior appendages with apical spine almost as long as rest of the superior appendages (Fig. 131); in ventral view, sternal processes almost parallel-sided along their length (Fig. 132)	***A. viklundi* sp. nov.**
–	Superior appendages with apical spine approx. 1/3 the length of the rest of the superior appendages (Fig. 147); in ventral view, sternal processes divided into an almost parallel-sided basal half and a oval distal half (Fig. 148)	***A. guttata* sp. nov.**
30	In dorsal view, apical spines of superior appendages very thin, needle-like (Fig. 136); in lateral view inferior appendages surpassing posteriorly mid-length of sternal processes (Fig. 135)	***A. lata* sp. nov.**
–	In dorsal view, apical spines of superior appendages very broad, hook-like (Fig. 75); in lateral view inferior appendages not surpassing posteriorly mid-length of sternal processes (Fig. 74)	***A. cunicula* sp. nov.**
31	In lateral view, inferior appendages with base forming almost rectangular plate more than half the total height of the genitalia (Figs 232, 237)	**32**
–	In lateral view, inferior appendages with base being lower than half the total height of the genitalia (Figs 212, 227)	**35**
32	In lateral view, superior appendages with almost straight dorsal margin (Fig. 232)	**33**
–	In lateral view, superior appendages with convex dorsal margin (Fig. 237)	**34**
33	Sternal processes constituting two distinct, slender, needle-shaped rays (fig. 3 in Ward (2003))	***A. hexacantha* Ward, 2003**
–	Sternal processes constituting one distinct, slender ray (Fig. 232)	***A. ninguana* sp. nov.**
34	In lateral view, sternal process sigmoid (Fig. 237)	***A. scopula* sp. nov.**
–	In lateral view, sternal process straight (Ward and Schefter 2000: fig. 53)	***A. diriwi* Ward & Schefter, 2000**
35	In ventral view, plate of inferior appendages minute or absent (Figs 81, 187)	**36**
–	In ventral view, plate of inferior appendages well developed (Figs 7, 112, 209)	**38**
36	In lateral view, sternal processes with triangular apex (Fig. 185)	***A. recurvata* sp. nov.**
–	In lateral view, sternal processes with finger-like apex (Fig. 185)	**37**
37	In lateral view, sternal process with finger-like apex shorter than maximum thickness of inferior appendages (Fig. 79)	***A. cerritula* sp. nov.**
–	In lateral view, sternal process with finger-like apex more than 2 times longer than maximum thickness of inferior appendages (Fig. 84)	***A. monstrosa* sp. nov.**
38	In ventral view, plate of inferior appendages with posterolateral branches along ventral margin (Figs 3, 7, 214)	**39**
–	In ventral view, plate of inferior appendages lacking posterolateral branches along ventral margin (Figs 158, 163; Ward 2003: fig. 7)	**63**
39	In ventral view, plate of inferior appendages with posterolateral branches along ventral margin separated by wide and deep incision (Figs 3, 7, 192)	**40**
–	In ventral view, plate of inferior appendages with posterolateral branches along ventral margin separated by a narrow and short incision (Figs 112, 153; Ward 2003: fig. 5)	**59**
40	In ventral view, plate of inferior appendages with posterolateral branches along ventral margin running almost in parallel to each other (Figs 3, 7, 192)	**41**
–	In ventral view, plate of inferior appendages with posterolateral branches along ventral margin converging or diverging distally (Figs 3, 7, 192)	**45**
41	In lateral view, superior appendages ending in long, finger-like ventrally orientated process (fig. 32 in Ward and Schefter (2000))	***A. panda* Ward & Schefter, 2000**
–	In lateral view, superior appendages without long, finger-like ventrally orientated process (Figs 1, 190)	**42**
42	In ventral view, plate of inferior appendages with posterolateral branches along ventral margin tapering posteriorly (Fig. 192); mesal process of superior appendages orientated mesally (Fig. 191)	**43**
–	In ventral view, plate of inferior appendages with posterolateral branches along ventral margin almost parallel-sided posteriorly; mesal process of superior appendages orientated posteriorly (Figs 1, 2)	**44**
43	In lateral view, sternal process rounded posteriorly (Fig. 190)	***A. taoensis* sp. nov.**
–	In lateral view, sternal process pointed posteriorly (fig. 4 in Ward (2003))	***A. arator* Ward, 2003**
44	In dorsal view, posterior part of parameres with row of megasetae on mesal margin (Fig. 2)	***A. tuberosa* sp. nov.**
–	In dorsal view, posterior part of parameres without row of megasetae on mesal margin (Fig. 7)	***A. semiovale* sp. nov.**
45	In lateral view, inferior appendages each with a dorsal and a ventral branch, and the dorsal branch is more than half the length of the ventral branch (Figs 201, 206)	**46**
–	In lateral view, inferior appendages each with only one branch (Figs 20, 25, 212)	**54**
46	In lateral view, each inferior appendage with dorsal branch longer than ventral branch (Fig. 15)	**47**
–	In lateral view, each inferior appendage with dorsal branch as long as or shorter than ventral branch (Fig. 201)	**49**
47	In lateral view, superior appendages widening posteriorly (fig. 28 in Ward and Schefter (2000)); inferior appendages with ventral branch forming a single plate (fig. 28 in Ward and Schefter (2000))	***A. padi* Ward & Schefter, 2000**
–	In lateral view, superior appendages narrowing posteriorly (Fig. 15); inferior appendages with ventral branch forming a pair of processes (Fig. 17)	**48**
48	In lateral view, parameres with a long process expanding below basis if superior appendages (Fig. 15)	***A. tenuisa* sp. nov.**
–	In lateral view, parameres without long process below superior appendages	***A. vuegi* Ward & Schefter, 2000**
49	In lateral view, each inferior appendage with dorsal and ventral branches narrowly separated at bases (figs 17 and 20 in Ward and Schefter (2000))	**50**
–	In lateral view, each inferior appendage with dorsal and ventral branches widely separated at bases (Figs 201, 206)	**51**
50	In lateral view, sternal processes almost straight and orientated posteriorly (fig. 20 in Ward and Schefter (2000)); each inferior appendage with dorsal branch bending dorsally at mid-length (fig. 20 in Ward and Schefter (2000))	***A. hircina* Ward & Schefter, 2000**
–	In lateral view, sternal processes curving ventrally along their length (fig. 17 in Ward and Schefter (2000)); each inferior appendage with dorsal branch sub-straight (fig. 17 in Ward and Schefter (2000))	***A. hirta* Ward & Schefter, 2000**
51	In lateral view, sternal processes slightly curving ventrally along their length (Fig. 201); paraprocts without ventrally orientated megasetae under basis of superior appendages (Fig. 206)	***A. bleuensis* sp. nov.**
–	In lateral view, sternal processes sharply bending ventrally after mid-length (Fig. 206); paraprocts with ventrally orientated megasetae (Fig. 206)	**52**
52	In lateral view, dorsal branch of inferior appendages almost straight (Fig. 206)	***A. touhoensis* sp. nov.**
–	In lateral view, dorsal branch of inferior appendages strongly bent ventrally (Ward and Schefter 2000: fig. 14)	***A. hamata* Ward & Schefter, 2000**
53	In lateral view, sternal processes narrowing uniformly along their length (Ward and Schefter 2000: fig. 23)	***A. kara* Ward & Schefter, 2000**
–	In lateral view, sternal processes narrowing abruptly almost at their mid-length (Ward 2000: fig. 2, Ward and Schefter 2003: fig. 25)	**70**
54	In lateral view, sternal processes short, not reaching basis of superior appendages (Figs 20, 25)	**55**
–	In lateral view, sternal processes long, exceeding basis of superior appendages (Fig. 212)	**56**
55	In lateral view, superior appendages almost three times longer than high (Fig. 20); inferior appendages high along their length (Fig. 20); in ventral view, plate of inferior appendages with posterior incision shallower than half length of plate (Fig. 22)	***A. multidentata* sp. nov.**
–	In lateral view, superior appendages approx. two times longer than high (Fig. 25); inferior appendages high only at basis (Fig. 25); in ventral view, plate of inferior appendages with posterior incision deeper than half length of plate (Fig. 28)	***A. cornuta* sp. nov.**
56	In lateral view, sternal processes uniformly curving ventrally along their length (Fig. 50)	**57**
–	In lateral view, sternal processes bending in right angle and almost straight after bending point (Fig. 212)	**58**
57	In lateral view, sternal processes broad at basis (fig. 50 in Ward and Schefter (2000)); each inferior appendage with a mesal branch (Ward and Schefter 2000: fig. 51)	***A. bimaculata* Ward & Schefter, 2000**
–	In lateral view, sternal processes slender at basis (fig. 1 in Ward (2003)); each inferior appendage without a mesal branch (Ward 2003: fig. 1)	***A. comata* Ward, 2003**
58	In lateral view, sternal processes short, not exceeding basis of inferior appendages (Fig. 212); each inferior appendage angled dorsally after basis (Fig. 212); superior appendages with uniformly convex ventral margin (Fig. 212); in ventral view, plate of inferior appendages with laterally curving lateral processes (Fig. 214)	***A. wardi* sp. nov.**
–	In lateral view, sternal processes long, almost reaching apex of inferior appendages (fig. 11 in Ward and Schefter (2000)); each inferior appendage straight after basis (Ward & Schefter: fig. 11 2000); superior appendages with angled ventral margin (Ward and Schefter 2000: fig. 11); in ventral view, plate of inferior appendages with almost straight posteriorly orientated lateral processes (Ward and Schefter 2000: fig. 12)	***A. hastata* Ward & Schefter, 2000**
59	In lateral and dorsal view, superior appendages with a hook located apically (Figs 161, 162Ward and Schefter 2000: figs 34 and 35)	**60**
–	In lateral and dorsal view, superior appendages with a spine located sub-apically (Fig. 152) or spine and hook absent (Figs 111, 156)	**61**
60	Almost all the superior appendages constituting the hook (Fig. 162)	***A. complexa* sp. nov.**
–	Only distal part of the superior appendages constituting the hook (Ward and Schefter 2000: fig. 35)	***A. berada* Ward & Schefter, 2000**
61	In lateral view, superior appendage more than 3 times longer than high (Fig. 156)	***A. spina* sp. nov.**
–	In lateral view, superior appendage less than 3 times longer than high (Figs 110, 151)	**62**
62	In lateral view, superior appendage about 2 times longer than high (Ward 2003: fig. 5); in lateral view, inferior appendages with three finger-like posterior processes (Ward 2003: fig. 5)	***A. tridactyla* Ward, 2003**
–	In lateral view, superior appendage less than 1.5 times longer than high (Figs 110, 151); in lateral view, inferior appendages without finger-like posterior processes (Figs 110, 152)	**72**
63	In lateral view, sternal processes well developed (Figs 31, 180)	**64**
–	In lateral view, sternal processes minute or absent	**67**
64	In lateral view, sternal processes with posterior 1/3 uniformly slender and strongly curving ventrally (Fig. 180); paraproct exceeding superior appendages posteriorly (Fig. 180)	***A. curvatacua* sp. nov.**
–	In lateral view, sternal processes almost straight and uniformly narrowing apically (Figs 31, 175); paraproct not exceeding superior appendages posteriorly (Figs 31, 99, 175)	**65**
65	In lateral view, superior appendage shorter than tergum X (Fig. 31)	***A. sagittata* sp. nov.**
–	In lateral view, superior appendage longer than tergum X (Figs 99, 175)	**66**
66	In ventral view, plate of inferior appendages with long posterior central process (Fig. 102)	**71**
–	In ventral view, plate of inferior appendages without posterior central process (Fig. 177)	**74**
67	In lateral and dorsal view, parameres with group of megasetae above basis of superior appendages (Fig. 222; Ward and Schefter 2000: figs 56, 58)	**68**
–	Parameres lacking group of megasetae (Figs 99, 217, 227)	**69**
68	In lateral view, inferior appendages constituting a prominent dorsal and ventral branch (Fig. 222); superior appendages ovoid (Fig. 222); in ventral view, plate of inferior appendages with posterior central process longer than rest of the plate (Fig. 224)	***A. christinae* sp. nov.**
–	In lateral view, inferior appendages constituting two smaller dorsal branches and a prominent ventral branch (Ward and Schefter 2000: fig. 56); superior appendages almost rectangular (Ward and Schefter 2000: fig. 56); in ventral view, plate of inferior appendages with posterior central process shorter than rest of the plate (Ward and Schefter 2000: fig. 57)	***A. rhara* Ward & Schefter, 2000**
69	In lateral view, superior appendages club-shaped (Fig. 217); inferior appendages with dorsal and ventral branches deeply separated (Fig. 217); in ventral view, plate of inferior appendages with straight lateral margins (Fig. 219)	***A. parallela* sp. nov.**
–	In lateral view, superior appendages almost rectangular (Fig. 227); inferior appendages with dorsal and ventral branches narrowly separated (Fig. 227); in ventral view, plate of inferior appendages with convex lateral margins (Fig. 229)	***A. brevis* sp. nov.**
70	In lateral view, basal half of inferior appendages about 2 times wider than distal half (Ward and Schefter 2000: fig. 25)	***A. mariae* Ward & Schefter, 2000**
–	In lateral view, basal half of inferior appendages more than four times wider than distal half (Ward 2000: fig. 2)	***A. acula* Ward, 2003**
71	In ventral view, plate of inferior appendages with undivided posterior central process (Fig. 102)	***A. piscaria* sp. nov.**
–	In ventral view, plate of inferior appendages with deeply incised posterior central process (Ward and Schefter 2000: fig. 31)	***A. parie* Ward & Schefter, 2000**
72	Superior appendages each with single, spine-like mesal process curving mesally along its length (Fig. 152); parameres without megasetae	***A. amieuensis* sp. nov.**
–	Superior appendages each with weakly defined spine-like mesal process straight and orientated mesoposteriorly along its length (Fig. 111); posterior end of parameres with cluster of megasetae (Figs 110, 111)	***A. caraffa* sp. nov.**
73	In lateral view, superior appendages with dorsal branch wider than ventral branch (Fig. 105); inferior appendages situated dorsally of sternal processes (Fig. 105)	***A. amplexa* sp. nov.**
–	In lateral view, superior appendages with dorsal branch as narrow as ventral branch (fig. 56 in Ward (2003)); inferior appendages situated ventrally of sternal processes (Ward 2003: fig. 56)	***A. cheirella* Ward, 2003**
74	In lateral view, superior appendages with hook-shaped apex (Fig. 175)	***A. anterohamata* sp. nov.**
–	In lateral view, superior appendages with triangular apex (Ward 2003: fig. 7)	***A. pugnea* Ward, 2003**

## Discussion

The first Trichoptera from New Caledonia were described by Kimmins (1953), and including the species described here this number has now risen to 286, of which all but four species are endemic. The total diversity is expected to be even higher (Espeland and Johanson 2010a, Johanson and Wells 2019), and more work is needed to fully understand the biodiversity of the New Caledonian islands. Currently, very little is known about the early stages of New Caledonian species, and nothing at all is known about the early stages of members of the *Agmina* or *Caledomina* (Mary 2017). Like other members of the Ecnomidae, larvae are probably predatory and construct tubes of silk and fine sands, attached to various substrates. Associating adults and larvae using DNA barcoding (Johanson 2007) would be an important next step to increase the knowledge about *Agmina* and New Caledonian Trichoptera as a whole, and would make them available as valuable indicators for freshwater quality.

## Supplementary Material

XML Treatment for Agmina tuberosa

XML Treatment for Agmina semiovale

XML Treatment for Agmina rocheta

XML Treatment for Agmina tenuisa

XML Treatment for Agmina multidentata

XML Treatment for Agmina cornuta

XML Treatment for Agmina sagittata

XML Treatment for Agmina circulata

XML Treatment for Agmina digitata

XML Treatment for Agmina longispina

XML Treatment for Agmina magnahamata

XML Treatment for Agmina longicordata

XML Treatment for Agmina campanula

XML Treatment for Agmina semicampanula

XML Treatment for Agmina cunicula

XML Treatment for Agmina cerritula

XML Treatment for Agmina monstrosa

XML Treatment for Agmina rectangulata

XML Treatment for Agmina chela

XML Treatment for Agmina piscaria

XML Treatment for Agmina amplexa

XML Treatment for Agmina caraffa

XML Treatment for Agmina rostrata

XML Treatment for Agmina dathioensis

XML Treatment for Agmina rougensis

XML Treatment for Agmina viklundi

XML Treatment for Agmina lata

XML Treatment for Agmina falx

XML Treatment for Agmina guttata

XML Treatment for Agmina amieuensis

XML Treatment for Agmina spina

XML Treatment for Agmina complexa

XML Treatment for Agmina dognyensis

XML Treatment for Agmina mana

XML Treatment for Agmina anterohamata

XML Treatment for Agmina curvatacua

XML Treatment for Agmina recurvata

XML Treatment for Agmina taoensis

XML Treatment for Agmina triangulata

XML Treatment for Agmina bleuensis

XML Treatment for Agmina touhoensis

XML Treatment for Agmina wardi

XML Treatment for Agmina parallela

XML Treatment for Agmina christinae

XML Treatment for Agmina brevis

XML Treatment for Agmina ninguana

XML Treatment for Agmina scopula
